# Lisocabtagene maraleucel in follicular lymphoma: the phase 2 TRANSCEND FL study

**DOI:** 10.1038/s41591-024-02986-9

**Published:** 2024-06-03

**Authors:** Franck Morschhauser, Saurabh Dahiya, M. Lia Palomba, Alejandro Martin Garcia-Sancho, Juan Luis Reguera Ortega, John Kuruvilla, Ulrich Jäger, Guillaume Cartron, Koji Izutsu, Martin Dreyling, Brad Kahl, Hervé Ghesquieres, Kirit Ardeshna, Hideki Goto, Anna Maria Barbui, Jeremy S. Abramson, Peter Borchmann, Isabelle Fleury, Stephan Mielke, Alan Skarbnik, Sven de Vos, Manali Kamdar, Reem Karmali, Andreas Viardot, Thalia Farazi, Omotayo Fasan, James Lymp, Min Vedal, Rina Nishii, Ariel Avilion, Jessica Papuga, Jinender Kumar, Loretta J. Nastoupil

**Affiliations:** 1 Centre Hospitalier Universitaire de Lille, Groupe de Recherche sur les formes Injectables et les Technologies Associées, Lille, France; 2 Stanford University School of Medicine, Stanford, CA USA; 3 University of Maryland Greenebaum Comprehensive Cancer Center, Baltimore, MD USA; 4 Memorial Sloan Kettering Cancer Center, New York, NY USA; 5 Hospital Universitario de Salamanca, IBSAL, CIBERONC, Centro de Investigación del Cáncer-IBMCC (USAL-CSIC), Salamanca, Spain; 6 Hospital Virgen del Rocío, Instituto de Biomedicina de la Universidad de Sevilla, Seville, Spain; 7 Princess Margaret Cancer Centre, Toronto, Ontario Canada; 8 Medical University of Vienna, Vienna, Austria; 9 Montpellier University Hospital Center, UMR CNRS 5535, Montpellier, France; 10 National Cancer Center Hospital, Tokyo, Japan; 11 LMU University Hospital, München, Germany; 12 Washington University School of Medicine in St. Louis, St. Louis, MO USA; 13 Hôpital Lyon Sud, Lyon, France; 14 University College London Hospitals Biomedical Research Centre, London, UK; 15 Hokkaido University Hospital, Sapporo, Japan; 16 Azienda Socio Sanitaria Territoriale Papa Giovanni XXIII, Bergamo, Italy; 17 Massachusetts General Hospital Cancer Center, Harvard Medical School, Boston, MA USA; 18 Universität zu Köln, Köln, Germany; 19 Hôpital Maisonneuve – Rosemont, Montreal, Quebec Canada; 20 Karolinska Institutet and University Hospital, Karolinska Comprehensive Cancer Center, Karolinska ATMP Center, Stockholm, Sweden; 21 Novant Health Cancer Institute, Charlotte, NC USA; 22 UCLA Santa Monica Medical Centre, Santa Monica, CA USA; 23 University of Colorado Cancer Center, Aurora, CO USA; 24 Northwestern University Feinberg School of Medicine, Robert H. Lurie Comprehensive Cancer Center, Chicago, IL USA; 25 Department of Internal Medicine III, University Hospital, Ulm, Germany; 26 Bristol Myers Squibb, Brisbane, CA USA; 27 Bristol Myers Squibb, Princeton, NJ USA; 28 Bristol Myers Squibb, Seattle, WA USA; 29 Bristol Myers Squibb, Boudry, Switzerland; 30 The University of Texas MD Anderson Cancer Center, Houston, TX USA

**Keywords:** B-cell lymphoma, Medical research

## Abstract

An unmet need exists for patients with relapsed/refractory (R/R) follicular lymphoma (FL) and high-risk disease features, such as progression of disease within 24 months (POD24) from first-line immunochemotherapy or disease refractory to both CD20-targeting agent and alkylator (double refractory), due to no established standard of care and poor outcomes. Chimeric antigen receptor (CAR) T cell therapy is an option in R/R FL after two or more lines of prior systemic therapy, but there is no consensus on its optimal timing in the disease course of FL, and there are no data in second-line (2L) treatment of patients with high-risk features. Lisocabtagene maraleucel (liso-cel) is an autologous, CD19-directed, 4-1BB CAR T cell product. The phase 2 TRANSCEND FL study evaluated liso-cel in patients with R/R FL, including 2L patients who all had POD24 from diagnosis after treatment with anti-CD20 antibody and alkylator ≤6 months of FL diagnosis and/or met modified Groupe d’Etude des Lymphomes Folliculaires criteria. Primary/key secondary endpoints were independent review committee–assessed overall response rate (ORR)/complete response (CR) rate. At data cutoff, 130 patients had received liso-cel (median follow-up, 18.9 months). Primary/key secondary endpoints were met. In third-line or later FL (*n* = 101), ORR was 97% (95% confidence interval (CI): 91.6‒99.4), and CR rate was 94% (95% CI: 87.5‒97.8). In 2L FL (*n* = 23), ORR was 96% (95% CI: 78.1‒99.9); all responders achieved CR. Cytokine release syndrome occurred in 58% of patients (grade ≥3, 1%); neurological events occurred in 15% of patients (grade ≥3, 2%). Liso-cel demonstrated efficacy and safety in patients with R/R FL, including high-risk 2L FL. ClinicalTrials.gov identifier: NCT04245839.

## Abstract (web summary)

Results of the phase 2 trial of CD19 CAR T cell therapy lisocabtagene maraleucel in patients with relapsed or refractory follicular lymphoma, most of whom were treated in a third-line or later setting, show encouraging objective response rates in these high-risk patients.

## Main

Follicular lymphoma (FL) is the most common subtype of indolent non-Hodgkin lymphoma (iNHL), accounting for 12‒32% of NHLs in North America, Western Europe and Japan^1–4^. First-line treatment typically includes immunochemotherapy (for example, cyclophosphamide, doxorubicin, vincristine and prednisone + rituximab (R-CHOP) or obinutuzumab; bendamustine + rituximab or obinutuzumab)^5,6^. Introduction of rituximab resulted in better overall survival (OS) for patients with FL, with 10-year OS rates of approximately 80% (ref. ^7^). However, lymphoma remains the primary cause of death for 10% of patients^7^, and patients with progression of disease within 24 months (POD24) from first-line immunochemotherapy have inferior OS (5-year OS of 64%)^8^.

In second-line (2L) treatment for patients with relapsed/refractory (R/R) FL, physicians may consider chemotherapy, antibody monotherapy, immunotherapy or immunochemotherapy, particularly if long remission was achieved in the first-line setting^5,6^. In select cases (for example, early relapse or transformation), autologous stem cell transplantation may be considered^6^. However, for patients with high-risk disease features, such as POD24 or disease that is refractory to both a CD20-targeting agent and an alkylator (double refractory), outcomes with available therapies are inferior, and additional treatment options are needed^8,9^. Response rates decrease with each subsequent line of therapy, and there is no established standard of care^10^.

Chimeric antigen receptor (CAR) T cell therapies have shown efficacy in patients with R/R FL in the third-line or later (3L+) setting^11,12^. However, there is no consensus on the optimal timing of CAR T cell therapy in the disease course of FL, especially in 2L treatment of patients with high-risk disease. Lisocabtagene maraleucel (liso-cel), an autologous, CD19-directed, 4-1BB CAR T cell product composed of CD8^+^ and CD4^+^ CAR^+^ T cells, has previously shown deep and durable responses in patients with R/R large B cell lymphoma^13–16^. TRANSCEND FL is a phase 2, pivotal study to assess the efficacy and safety of liso-cel in adults with R/R iNHL. Here we report the primary analysis for patients in the R/R FL cohorts, including, to our knowledge, the first report of CAR T cell therapy in patients with 2L R/R FL.

## Results

### Patients and treatment

**Fig. 1:**
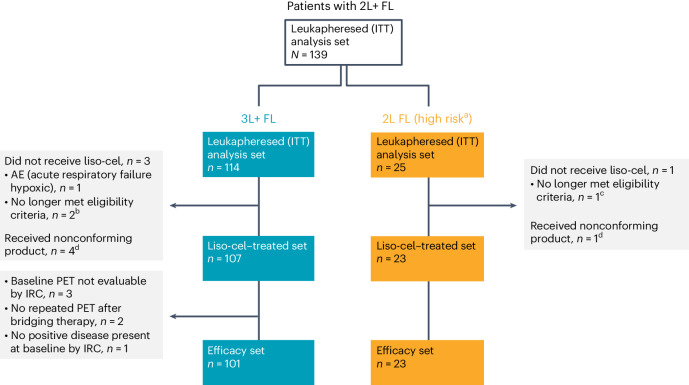
CONSORT diagram for patients with 3L+ FL and patients with high-risk 2L FL. ^a^The high-risk 2L FL cohort included patients with POD24 from diagnosis and/or who met mGELF criteria. ^b^One patient had history of transformed FL, and one patient had PET-negative disease at pre-treatment assessment. ^c^Reached CR after bridging therapy per investigator assessment and had PET-negative disease at pre-treatment assessment. ^d^Non-conforming product was defined as any product wherein one of the CD8 or CD4 cell components did not meet one of the requirements to be considered liso-cel but was considered appropriate for infusion.

From 14 July 2020 to 27 January 2023, 139 patients were enrolled and leukapheresed in the FL cohorts (2L or later (2L+), *n* = 139; 3L+, *n* = 114; 2L, *n* = 25) at 31 sites in North America, Europe and Japan. Liso-cel was successfully manufactured for 133 of 139 patients (96%). Four patients were not infused, including one with an adverse event (AE) of acute respiratory failure (enterovirus/rhinovirus pneumonia), one with transformed FL and two with positron emission tomography (PET)/computed tomography (CT)–negative disease at the pre-treatment assessment; five received non-conforming product (Fig. 1). Thus, 130 patients with 2L+ FL (2L, *n* = 23; 3L+, *n* = 107) received liso-cel (liso-cel–treated set) and 124 (2L, *n* = 23; 3L+, *n* = 101) were efficacy evaluable (EE) per independent review committee (IRC; efficacy set: all patients in the liso-cel–treated set who had PET/CT-positive disease per IRC before liso-cel administration; excluded patients who did not have a baseline assessment repeated after bridging therapy). In liso-cel–treated patients, median time from leukapheresis to liso-cel availability was 29 d (interquartile range (IQR): 25‒31), and time from leukapheresis to liso-cel infusion was 49 d (IQR: 41‒55) (Supplementary Table 1). At the data cutoff on 27 January 2023, median on-study follow-up was 18.9 months (range, 0.3‒28.2).

In the liso-cel–treated set, median age was 60 years (range, 23–80; 3L+, median 62 years; 2L, median 53 years); 86% had Ann Arbor stage III/IV disease (3L+, 89%; 2L, 74%); 53% had high-risk FL International Prognostic Index (FLIPI; 3L+, 57%; 2L, 35%); 45% had POD24 from diagnosis after treatment with an anti-CD20 antibody and an alkylating agent within the first 6 months of initial FL diagnosis (3L+, 43%; 2L, 52%); 56% met modified Groupe d’Etude des Lymphomes Folliculaires (mGELF) criteria (3L+, 53%; 2L, 70%); and 62% were double refractory (that is, refractory to both an anti-CD20 antibody and alkylating agent or to anti-CD20 maintenance, defined as patients whose disease did not respond or progressed during or up to 6 months after completing treatment with an anti-CD20 antibody and alkylating agent or maintenance treatment with an anti-CD20 antibody; 3L+, 64%; 2L, 48%) (Table 1 and Supplementary Table 2). Fifty-six percent of patients had POD24 from initiation of first-line systemic therapy with anti-CD20 antibody plus alkylator (3L+, 54%; 2L, 65%). The median time from diagnosis to liso-cel infusion was 4.7 years (range, 0.7‒35.3; 3L+, median 5.1 years; 2L, median 2.0 years). Additional patient-level disease characteristics are provided for patients with 2L FL in Supplementary Table 3. Bridging therapy for disease control during liso-cel manufacturing was used in 38% of patients (3L+, 41%; 2L, 22%) (Table 1 and Supplementary Table 2). Most bridging therapies were combination regimens, primarily rituximab plus gemcitabine and oxaliplatin (Supplementary Table 4).

**Table 1:** Demographic and baseline characteristics (liso-cel–treated set)^a^

	3L+ FL (*n* = 107)	2L FL (*n* = 23)	2L+ FL (*n* = 130)
Median age (range), years	62 (23‒80)	53 (34‒69)	60 (23‒80)
Male sex (biological attribute), *n* (%)	66 (62)	17 (74)	83 (64)
Primary race, *n* (%)			
Asian	10 (9)	2 (9)	12 (9)
Black or African American	3 (3)	1 (4)	4 (3)
White	60 (56)	9 (39)	69 (53)
Not collected or unknown^b^	34 (32)	11 (48)	45 (35)
ECOG PS at screening, *n* (%)			
0	65 (61)	17 (74)	82 (63)
1	42 (39)	6 (26)	48 (37)
FL subtype/grade at screening, *n* (%)			
Grade 1/2	81 (76)	17 (74)	98 (75)
Grade 3A	25 (23)	6 (26)	31 (24)
Unknown	1 (1)	0	1 (1)
Ann Arbor stage at screening, *n* (%)			
Stage I/II	12 (11)	6 (26)	18 (14)
Stage III	39 (36)	6 (26)	45 (35)
Stage IV	56 (52)	11 (48)	67 (52)
FLIPI at screening, *n* (%)			
Low risk (0‒1)	12 (11)	11 (48)	23 (18)
Intermediate risk (2)	34 (32)	4 (17)	38 (29)
High risk (3‒5)	61 (57)	8 (35)	69 (53)
SPD ≥ 50 cm^2^ before LDC per IRC, *n* (%)	22 (21)	3 (13)	25 (19)
LDH > ULN before LDC, *n* (%)	47 (44)	6 (26)	53 (41)
mGELF criteria met at time of most recent relapse, *n* (%)	57 (53)	16 (70)	73 (56)
Median prior lines of systemic therapy (range)	3 (2‒10)	1 (1‒1)	2 (1‒10)
Prior HSCT^c^, *n* (%)	33 (31)	0	33 (25)
Received prior rituximab and lenalidomide, *n* (%)	23 (21)	0	23 (18)
Prior bendamustine, *n* (%)			
No prior bendamustine	42 (39)	17 (74)	59 (45)
Prior bendamustine ≤6 months before leukapheresis	4 (4)	1 (4)	5 (4)
Prior bendamustine >6 months and ≤12 months before leukapheresis	4 (4)	2 (9)	6 (5)
Prior bendamustine >12 months before leukapheresis	57 (53)	3 (13)	60 (46)
Refractory to systemic therapy^d^, *n* (%)	38 (36)	3 (13)	41 (32)
PD while on the last LOT or ≤6 months of completing the last LOT, *n* (%)	69 (64)	15 (65)	84 (65)
POD24 from diagnosis^e^, *n* (%)	46 (43)	12 (52)	58 (45)
FL progression ≤24 months of first-line therapy with anti-CD20 antibody and alkylator, *n* (%)	58 (54)	15 (65)	73 (56)
Double refractory (anti-CD20 + alkylator)^f^, *n* (%)	69 (64)	11 (48)	80 (62)
Median time-to-event analyses (range)			
Diagnosis to first PD, years	2.0 (0.25‒16.5)	1.8 (0.5‒11.2)	2.0 (0.25‒16.5)
Initial treatment to first PD, years	1.5 (0.1‒8.8)	1.4 (0.3‒11.1)	1.5 (0.1‒11.1)
Completion of last LOT to SD or PD^g^, years	0.15 (0‒9.6)	0.3 (0‒8.8)	0.15 (0‒9.6)
Diagnosis to liso-cel infusion, years	5.1 (0.7‒35.3)	2.0 (0.8‒11.4)	4.7 (0.7‒35.3)
Most recent relapse to liso-cel infusion, years	0.4 (0‒3.2)	0.3 (0.1‒1.3)	0.3 (0‒3.2)
Received bridging therapy, *n* (%)	44 (41)	5 (22)	49 (38)

^a^Percentages may not add up to 100% due to rounding.

^b^Due to some European country regulations.

^c^All prior HSCT was autologous HSCT.

^d^Defined as best response of SD or PD after prior therapy. If not refractory, then relapsed, defined as relapse after an initial response of CR or PR to the prior therapy.

^e^Defined as progression of disease within 24 months of diagnosis after treatment with anti-CD20 antibody and alkylator within 6 months of initial FL diagnosis.

^f^Refractory to both an anti-CD20 antibody and alkylating agent (defined as patients whose disease did not respond or progressed during or up to 6 months after completing treatment with an anti-CD20 antibody and alkylating agent) or refractory to anti-CD20 maintenance (defined as patients whose disease did not respond or progressed during or up to 6 months after completing maintenance treatment with an anti-CD20 antibody).

^g^Calculated by taking the day of progression (or SD if missing progression data) and subtracting the day of the last prior regimen completion. For patients who progressed before completion of their last prior line of anti-cancer therapy, the start date of range was set to 0.

ECOG PS, Eastern Cooperative Oncology Group performance status; HSCT, hematopoietic stem cell transplantation; LDH, lactate dehydrogenase; LOT, line of therapy; PR, partial response; SD, stable disease; SPD, sum of the product of perpendicular diameters; ULN, upper limit of normal.

### Efficacy

**Fig. 2:**
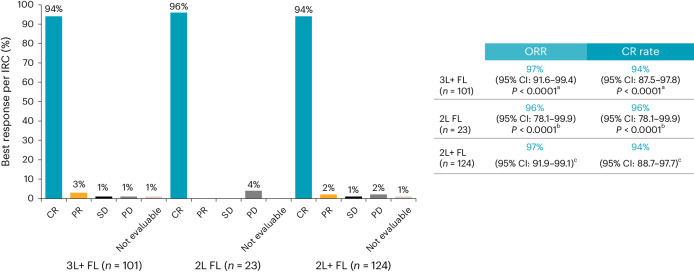
ORR by best overall response per IRC assessment. ^a^One-sided *P* value using exact binomial test (H_0_ of ORR ≤ 60%; H_0_ of CR rate ≤ 30%). ^b^One-sided *P* value using exact binomial test (H_0_ of ORR ≤ 50%; H_0_ of CR rate ≤ 19%). ^c^Not statistically tested (descriptive). H_0_, null hypothesis; PR, partial response; SD, stable disease.

**Fig. 3:**
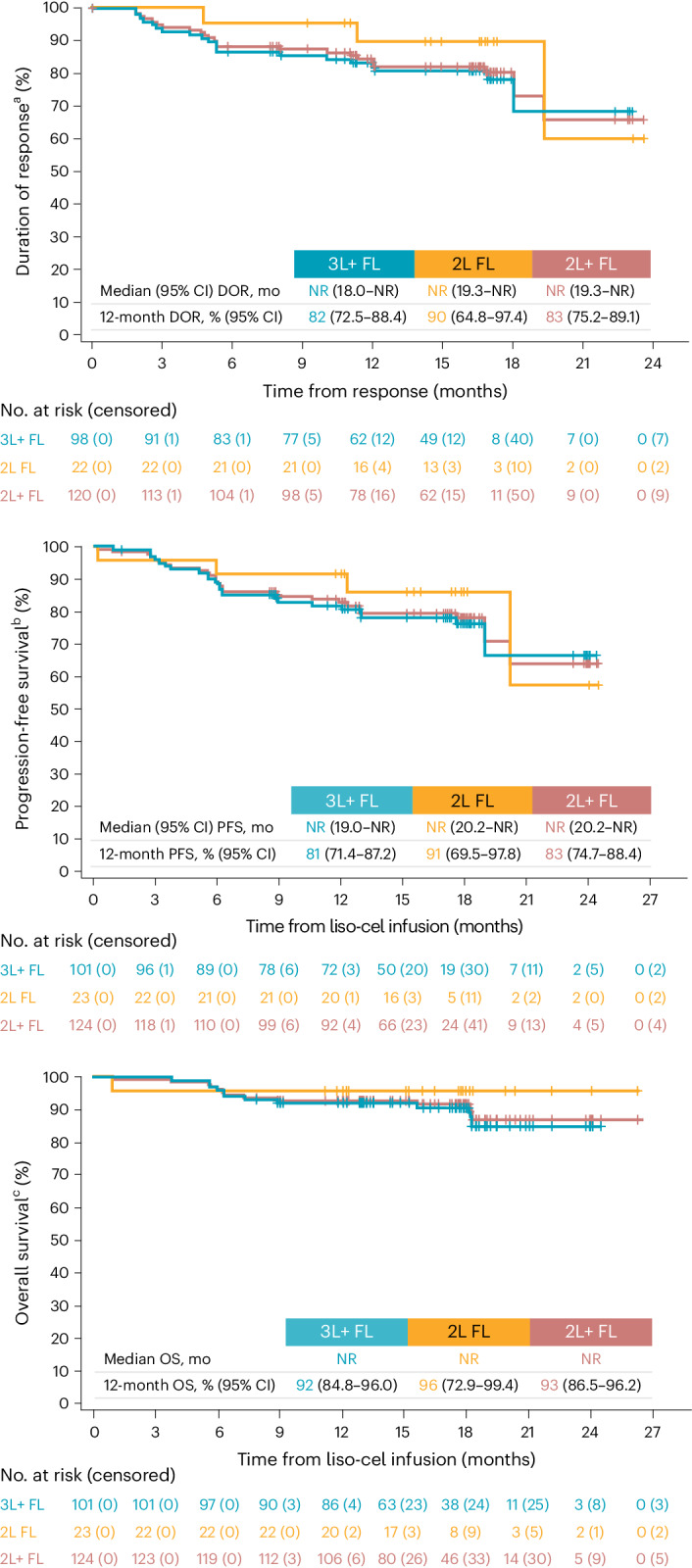
Kaplan–Meier curves for DOR, PFS and OS (efficacy set). Kaplan–Meier data are presented as median (95% CI). Median follow-up was calculated using the reverse Kaplan–Meier method. ^a^DOR was defined as time from first response to PD or death from any cause. Median follow-up for DOR in 2L+ was 16.7 months (95% CI: 16.2‒16.9). ^b^PFS was defined as time from liso-cel infusion to PD or death from any cause. Median follow-up for PFS in 2L+ was 17.6 months (95% CI: 17.1‒17.8). ^c^OS was defined as time from liso-cel infusion to time of death from any cause. Median follow-up for OS in 2L+ was 17.9 months (95% CI: 17.45‒18.0). A total of 90% of patients in the efficacy set were censored from the OS analysis at data cutoff. mo, months.

In patients with 3L+ FL, the overall response rate (ORR) was 97% (95% confidence interval (CI): 91.6‒99.4; *P* < 0.0001), with 92 of 95 responders achieving complete response (CR); the CR rate was 94% (95% CI: 87.5‒97.8; *P* < 0.0001) (Fig. 2 and Supplementary Tables 5 and 6). Median time to first response was 1 month (range, 0.6‒3.3). Median duration of response (DOR) was not reached (NR; 95% CI: 18.0‒NR) at a median follow-up of 16.6 months, and the 12-month DOR rate was 82% (95% CI: 72.5‒88.4) (Fig. 3). Median progression-free survival (PFS) was NR (95% CI: 19.0‒NR) at median follow-up of 17.6 months, and the 12-month PFS rate was 81% (95% CI: 71.4‒87.2). Median OS was NR, and the 12-month OS rate was 92% (95% CI: 84.8‒96.0).

In patients with 2L FL, the ORR was 96% (95% CI: 78.1‒99.9; *P* < 0.0001), with all responders achieving a CR. Median time to first response was 1 month (range, 0.8‒2.8) (Fig. 2). Median DOR was NR (95% CI: 19.3‒NR) at a median follow-up of 16.8 months, and the 12-month DOR rate was 90% (95% CI: 64.8‒97.4) (Fig. 3). Median PFS was NR (95% CI: 20.2‒NR) at a median follow-up of 17.8 months, and the 12-month PFS rate was 91% (95% CI: 69.5‒97.8). Median OS was NR, and the 12-month OS rate was 96% (95% CI: 72.9‒99.4).

Results from the efficacy set subgroup analyses were consistent with the primary analysis. ORR, CR rate, DOR and PFS (defined by 12-month estimates of continued response rate and PFS rate) remained high across patient subgroups, including those with high-risk disease features (Supplementary Figs. 1‒8).

Response rates were similar in the intent-to-treat (ITT) (leukapheresed) population, with ORR of 93% (95% CI: 86.6‒96.9) and CR rate of 90% (95% CI: 83.4‒95.1) in 3L+ FL and ORR of 92% (95% CI: 74.0‒99.0) in 2L FL, with all responders achieving a CR (Supplementary Table 7). Among liso-cel–treated patients who received bridging therapy, ORR was 95% (38/40) in 3L+ FL and 80% (4/5) in 2L FL, with all responders achieving CR. The ORR per investigator assessment was 98% (99/101) in 3L+ FL and 100% (23/23) in 2L FL (Supplementary Table 8). The study demonstrated 95% concordance between IRC-assessed and investigator-assessed best overall response (BOR) in patients with 2L+ FL.

### Safety

Among liso-cel–treated patients, 97 (75%: 3L+, 83 (78%); 2L, 14 (61%)) had grade ≥3 treatment-emergent adverse events (TEAEs) and 32 (25%: 3L+, 28 (26%); 2L, 4 (17%)) experienced serious TEAEs. The most common grade ≥3 TEAEs were cytopenias, including neutropenia in 76 patients (58%: 3L+, 64 (60%); 2L, 12 (52%)) and anemia and thrombocytopenia in 13 patients (10%: 3L+, 12 (11%); 2L, 1 (4%)) each (Table 2 and Supplementary Table 9). Eight patients (6%: 3L+, 6 (6%); 2L, 2 (9%)) had febrile neutropenia.

**Table 2:** Most common TEAEs^a^ (≥10%) in patients with 2L+ FL (liso-cel–treated set)

	2L+ FL (*n* = 130)	
TEAE, *n* (%)	Any grade	Grade ≥3
Neutropenia	85 (65)	76 (58)
CRS	75 (58)	1 (1)
Anemia	49 (38)	13 (10)
Headache	38 (29)	0
Thrombocytopenia	33 (25)	13 (10)
Constipation	26 (20)	0
Pyrexia	23 (18)	0
Diarrhea	22 (17)	0
Lymphopenia	20 (15)	17 (13)
Fatigue	19 (15)	0
Tremor	18 (14)	0
Leukopenia	18 (14)	15 (12)
Asthenia	16 (12)	0

^a^ TEAE period was defined as the time from initiation of liso-cel administration through and including study day 90.

Any-grade cytokine release syndrome (CRS) occurred in 75 patients (58%: 3L+, 63 (59%); 2L, 12 (52%)) with a median onset of 6 d (range, 1‒17; 3L+, median 6 d; 2L, median 6 d) and median duration of 3 d (range, 1‒10; 3L+, median 4 d; 2L, median 3 d). Most CRS events were grade 1 (42%: 3L+, 45%; 2L, 30%), with grade 3 in only one patient (1%: 3L+, 1%; 2L, 0) and no grade 4 or 5 events. CRS was managed with tocilizumab alone in 18 patients (14%: 3L+, 15%; 2L, 9%) and both tocilizumab and corticosteroids in 15 patients (12%: 3L+, 13%; 2L, 4%). Two patients (2%: 3L+, 2%; 2L, 0) received vasopressors.

Any-grade neurological events (NEs), defined as investigator-identified neurological AEs related to liso-cel, occurred in 20 patients (15%: 3L+, 15%; 2L, 17%), with a median onset of 8.5 d (range, 4‒16; 3L+, median 8.5 d; 2L, median 8.5 d) and median duration of 3.5 d (range, 1‒17; 3L+, median 4.5 d; 2L, median 2.5 d). Most NEs were grade 1 (12%: 3L+, 11%; 2L, 13%), with grade 3 in three patients (2%: 3L+, 2%; 2L, 4%) and no grade 4 or 5 events (Table 3 and Supplementary Table 10). The most common any-grade treatment-emergent NE signs and symptoms were aphasia and tremor (*n* = 9 each; 3L+, *n* = 7 and *n* = 8, respectively; 2L, *n* = 2 and *n* = 1, respectively) and dyscalculia, dysgraphia and headache (*n* = 3 each; 3L+, *n* = 3 each; 2L, *n* = 0) (Supplementary Table 11). NEs were managed with corticosteroids alone in eight patients (6%: 3L+, 6%; 2L, 9%) and both tocilizumab and corticosteroids in one patient (1%: 3L+, 1%; 2L, 0). TEAEs of nervous system/psychiatric disorders regardless of attribution to liso-cel occurred in 68 patients (52%: 3L+, 49%; 2L, 70%), including, most commonly, headache in 38 patients (29%: 3L+, 28%; 2L, 35%) and tremor in 18 patients (14%: 3L+, 15%, 2L, 9%). Most events were grade 1 (33%: 3L+, 35%; 2L, 26%) or grade 2 (14%: 3L+, 10%; 2L, 30%), with grade 3 in seven patients (5%: 3L+, 4%; 2L, 13%) and no grade 4 or 5 events (Supplementary Table 12).

**Table 3:** AEs of special interest (liso-cel–treated set)

	2L+ FL (*n* = 130)
CRS^a^, *n* (%)	
Any grade	75 (58)
Grade 1	55 (42)
Grade 2	19 (15)
Grade 3^b^	1 (1)
Grade 4/5	0
Median time to first onset of CRS (range), d	6.0 (1‒17)
Median time to resolution of first CRS (range), d	3.0 (1‒10)
Treatment for CRS, *n* (%)	
Tocilizumab only	18 (14)
Corticosteroids only	0
Both tocilizumab and corticosteroid	15 (12)
Tocilizumab and/or corticosteroid	33 (25)
Vasopressors^c^	2 (2)
NEs^d^, *n* (%)	
Any grade	20 (15)
Grade 1	15 (12)
Grade 2	2 (2)
Grade 3^e^	3 (2)
Grade 4/5	0
Median time to first onset of NE (range), d	8.5 (4‒16)
Median time to resolution of first NE (range), d	3.5 (1‒17)
Treatment for NEs, *n* (%)	
Tocilizumab only	0
Corticosteroids only (dexamethasone)	8 (6)
Both tocilizumab and corticosteroid	1 (1)
Tocilizumab and/or corticosteroid	9 (7)
Vasopressors	0
Prolonged cytopenia^f^, *n* (%)	29 (22)
Grade ≥3 neutropenia at day 29 visit, *n* (%)	20 (15)
Recovered to grade ≤2 by day 60^g^, *n*/*N* (%)	12/20 (60)
Recovered to grade ≤2 by day 90^g^, *n*/*N* (%)	18/20 (90)
Grade ≥3 anemia at day 29 visit, *n* (%)	6 (5)
Recovered to grade ≤2 by day 60^g^, *n*/*N* (%)	2/6 (33)
Recovered to grade ≤2 by day 90^g^, *n*/*N* (%)	5/6 (83)
Grade ≥3 thrombocytopenia at day 29 visit, *n* (%)	19 (15)
Recovered to grade ≤2 by day 60^g^, *n*/*N* (%)	7/19 (37)
Recovered to grade ≤2 by day 90^g,h^, *n*/*N* (%)	11/19 (58)
Grade ≥3 infection^i^, *n* (%)	7 (5)
Hypogammaglobulinemia^j^, *n* (%)	6 (5)
Second primary malignancy^j,k^, *n* (%)	4 (3)
MAS/HLH, *n* (%)^b,l^	1 (1)

^a^Graded according to the Lee et al.^24^ criteria.

^b^AE led to ICU hospitalization (Supplementary Table 15).

^c^Includes one case of low-dose vasopressors.

^d^Defined as investigator-identified neurological AEs related to liso-cel and graded per the NCI CTCAE, version 5.0.

^e^The three patients with grade 3 NEs fully recovered by 1 d, 2 d and 17 d after onset.

^f^Defined as grade ≥3 laboratory abnormalities of neutropenia, anemia or thrombocytopenia on day 29. Of patients with prolonged cytopenia, 24 received GCSF from day 1 to day 28 for reasons of neutropenia (*n* = 16), intermittent neutropenia (*n* = 2), prophylaxis for neutropenia/neutrophil count wasting (*n* = 2), confirmed neutrophil count decreased (*n* = 1), febrile neutropenia (*n* = 1), thrombocytopenia (*n* = 1) and worsening neutropenia (*n* = 1). Twenty-two patients received GCSF from day 29 onward for reasons of neutropenia (*n* = 13), worsening neutropenia (*n* = 3), intermittent neutropenia (*n* = 2), prophylaxis for neutropenia/neutrophil count wasting (*n* = 2), thrombocytopenia (*n* = 1) and bilateral pneumonia related to COVID-19 (*n* = 1).

^g^Recovery data are presented for patients with prolonged cytopenia who had laboratory results after day 29.

^h^Of the eight patients (42%) with unresolved grade ≥3 thrombocytopenia at day 90, seven had thrombocytopenia at baseline (grade 3 or 4, *n* = 4; grade 1 or 2, *n* = 3), and one patient with normal platelet counts at baseline had grade 4 thrombocytopenia at day 90 and died because of disease progression at day 180. Among the seven patients with thrombocytopenia at baseline, platelet counts had recovered to baseline levels in six patients, in two patients by day 90 and in four patients by day 180. The remaining patient had grade 4 thrombocytopenia at day 90 and died on day 114 because of a second primary malignancy of acute myeloid leukemia.

^i^Within the 90-d treatment-emergent period: bacterial urinary tract infection (*n* = 1), bronchitis (*n* = 1), COVID-19 and COVID-19 pneumonia (*n* = 1), device-related bacteremia (*n* = 1), *Escherichia* sepsis and pyelonephritis (*n* = 1), perforated appendicitis (*n* = 1) and pneumonia (*n* = 1); grade ≥3 infections occurring after the 90-d treatment-emergent period are provided in Supplementary Table 13.

^j^Could occur within or beyond the 90-d treatment-emergent period.

^k^AML (*n* = 2), rectal cancer (*n* = 1) and colon adenocarcinoma (*n* = 1).

^l^Grade 5 TEAE of MAS/HLH occurred in a 66-year-old male with 2L FL (stage IV), high-risk FLIPI, met mGELF criteria and had POD24 (that is, had achieved PR to first-line R-CHOP and progressed on rituximab maintenance ≤6 months of initiation of R-CHOP). At baseline, the patient’s bone marrow was more than 90% lymphoma with bone lesions and pleural effusions, and the patient received BR as bridging therapy. After liso-cel infusion, the patient had grade 2 CRS on days 2 and 5, which was treated with tocilizumab/steroids. MAS/HLH was treated with steroids and anakinra. Cytomegalovirus reactivation occurred at approximately day 21, and anakinra was stopped. Rebounded MAS/HLH was not responsive to treatment with anakinra, steroids and emapalumab. PET/CT scan on day 23 showed PR per investigator assessment and PD per IRC assessment, with death occurring on day 29. Before liso-cel treatment, the patient had pancytopenia and elevated ferritin based on laboratory assessment in peripheral blood. AML, acute myeloid leukemia; BR, bendamustine plus rituximab; GCSF, granulocyte colony-stimulating factor; PR, partial response.

Grade ≥3 infections were reported in seven patients (5%: 3L+, 7%; 2L, 0) within the 90-d treatment-emergent period. Grade ≥3 late infections (that is, >90-d TEAE period) occurred in three patients (all 3L+) (Supplementary Table 13). Prolonged cytopenia (grade ≥3 cytopenias based on laboratory values at day 29) was reported in 29 patients (22%: 3L+, 24%; 2L, 13%). Of patients with prolonged cytopenia and laboratory results after day 29, 18 of 20 (90%: 3L+, 89%; 2L, 100%) with neutropenia, five of six (83%: all 3L+) with anemia and 11 of 19 (58%: 3L+, 56%; 2L, 100%) with thrombocytopenia had recovered to grade ≤2 by day 90. Of the eight patients (42%) with unresolved grade ≥3 thrombocytopenia at day 90 (all 3L+), seven had thrombocytopenia at baseline, and platelet counts had recovered to baseline levels in six of those patients. Hypogammaglobulinemia as an AE (coded to specific Medical Dictionary for Regulatory Activities (MedDRA) preferred terms as described in the Methods) was reported in six patients (5%) (3L+, 5%; 2L, 4%). As neither the National Cancer Institute Common Terminology Criteria for Adverse Events (NCI CTCAE), version 5.0, nor the study protocol specified a threshold for immunoglobulin (Ig) levels to define an AE of hypogammaglobulinemia, we also analyzed available laboratory data (46 of 117 patients (39%: 3L+, 46%; 2L, 10%) had baseline serum IgG <500 mg dl^−1^). The proportion of patients with IgG <500 mg dl^−1^ did not change substantially over time, with incidence ranging from 50% of patients at day 29 to 56% of patients 1 year after liso-cel infusion. Mean baseline levels of serum IgA (79.6 mg dl^−1^) and IgM (44.1 mg dl^−1^) decreased by 44‒51% and 21‒47%, respectively, between day 29 and 1 year after liso-cel infusion. Among liso-cel–treated patients, 27 received concomitant IgG therapy, either for treatment or infection prophylaxis. Macrophage activation syndrome/hemophagocytic lymphohistiocytosis (MAS/HLH) and second primary malignancies were reported in one patient (1%: 3L+, 0; 2L, 4%) and four patients (3%: 3L+, 3%; 2L, 4%), respectively (Table 3).

There were 13 deaths (3L+, 12; 2L, 1) on-study, with one before liso-cel infusion (respiratory failure) and 12 after liso-cel infusion, including four due to disease progression (Supplementary Table 14). Two deaths were considered related to liso-cel by the investigator: one occurred in a patient with grade 5 TEAE of MAS/HLH and was the only death in the 2L FL cohort (reported in Table 3), and one occurred in a patient with an AE of progressive multifocal leukoencephalopathy after the 90-d treatment-emergent period. Patients with an intensive care unit (ICU) stay are reported in Supplementary Table 15.

Fifteen patients were monitored as outpatients (that is, liso-cel was administered in the outpatient facility or in the inpatient facility with subsequent discharge the same day at the end of the observation period) using standard operating procedures and multidisciplinary care teams. Patients and caregivers were educated to recognize early signs of CRS and NEs and remained within 1 h of the clinic for 30 d. Patients were monitored daily for the first 7 d and then at least twice weekly for the first month. Safety data for the 15 patients (12%: 3L+, 13%; 2L, 4%) monitored in the outpatient setting are reported in Supplementary Table 16. Of the 15 patients monitored in the outpatient setting, seven were hospitalized, with no ICU admissions. Median time to initial hospitalization was 7 d (range, 4‒16), and median duration of initial hospitalization was 5 d (range, 3‒8).

### Cellular kinetics and B cell aplasia

In 128 patients with evaluable cellular kinetic parameters (2L+ FL), liso-cel exhibited rapid expansion with a median time from liso-cel infusion to peak transgene levels (t_max_) of 10 d (IQR: 8‒11) after infusion (Supplementary Table 17 and Supplementary Fig. 9). Median peak expansion (C_max_) was 42,026 copies per microgram (IQR: 13,537‒110,390), and median area under the curve from 0 d to 28 d after infusion (AUC_(0‒28d)_) was 260,274 days×copies per microgram (IQR: 106,797‒673,556). Median t_max_, C_max_ and AUC_(0‒28d)_ were 10 d (IQR: 8‒11), 30,530 copies per microgram (IQR: 12,412‒96,795) and 253,400 days×copies per microgram (IQR: 105,912‒622,704) in 3L+ FL and 10 d (IQR: 9‒10), 62,091 copies per microgram (IQR: 45,428‒176,273) and 385,668 days×copies per microgram (IQR: 194,260‒921,947) in 2L FL, respectively. Persistence of liso-cel transgene levels was detected in 24 of 59 patients (41%) at 18 months (Supplementary Table 18). In the liso-cel–treated set (2L+ FL, *n* = 130), B cell aplasia incidence increased from 77% at baseline to 99% in patients after liso-cel infusion and remained at ≥91% through day 90 before gradually decreasing from month 6 onwards (57% at month 18) (Supplementary Table 19). Incidence of B cell aplasia was numerically lower at timepoints after day 90 in 2L FL compared to 3L+ FL, although the sample size for the 2L FL data was small and cannot be precisely interpreted.

### Patient-reported outcomes

For patient-reported outcome (PRO) measures, patients completed the European Organization for Research and Treatment of Cancer Quality of Life Questionnaire-Core 30 items (EORTC QLQ-C30) and the Functional Assessment of Cancer Therapy-Lymphoma ‘Additional Concerns’ Scale (FACT-LymS), which measures common lymphoma-specific symptoms and functioning. Of patients with 3L+ and 2L FL, completion rates for EORTC QLQ-C30 assessments were 70‒95% from baseline through month 18 (day 545) (Supplementary Fig. 10); completion rates were similar for FACT-LymS. In both 3L+ and 2L FL cohorts, the mean scores on most primary domains, including fatigue, pain, global health status and FACT-LymS, improved at the day 29 visit compared to baseline and were generally maintained throughout subsequent visits. The mean scores for most secondary domains also showed improvement by day 29, and this improvement was sustained throughout subsequent visits in both cohorts. Additionally, at some visits, the improvements exceeded the contemporary threshold for clinically meaningful improvement, similar to the primary domains (Supplementary Figs. 11‒13).

Overall least squares mean changes from baseline through day 730 showed statistically significant improvements in the following primary domains of interest: global health status (3L+ FL), fatigue (2L FL), pain (3L+ and 2L FL) and FACT-LymS (3L+ and 2L FL). Significant improvements were also observed in some secondary domains of interest in both cohorts (Supplementary Table 20). Median time to confirmed improvement occurred within 3 months across all primary domains in both cohorts, except for fatigue and FACT-LymS in 3L+ FL (Supplementary Figs. 14 and 15). In 3L+ FL, median time to confirmed improvement was 10.4 weeks for global health status, 11.1 weeks for physical functioning, 10.7 weeks for role functioning, 10.0 weeks for cognitive functioning, 27.1 weeks for fatigue, 5.0 weeks for pain and 27.1 weeks for FACT-LymS. Median time to confirmed improvement was shorter for 2L FL than 3L+ FL for all primary domains except global health status. In individual patient-level analyses, from day 29 onward, most patients with 3L+ FL reported improvement or no change across all primary domains (60‒93% at day 29, 71‒85% at day 90, 67‒81% at month 12 and 65‒83% at month 18) (Supplementary Figs. 16 and 17). Results were similar in 2L FL.

## Discussion

TRANSCEND FL evaluated CAR T cell therapy in the largest population of patients with R/R FL enrolled in a clinical trial to date and is, to our knowledge, the first study to report outcomes of CAR T cell therapy in patients with 2L R/R FL. The study population included patients with late-stage disease and high-risk disease features and with median age of 60 years; 86% had stage III/IV FL; 56% met mGELF criteria; 82% were intermediate or high risk per FLIPI; 65% had progressive disease (PD) during or ≤6 months of completing the last line of therapy; 56% had POD24 from initial immunochemotherapy; 62% were double refractory; and 38% received bridging therapy. The patient population in TRANSCEND FL was relatively young, which may have been mainly attributable to the willingness of physicians to provide cellular therapy to younger patients with high-risk disease who may have rapid progression after the last prior systemic therapy. Another contributing factor could have been the fairly short median time from initial treatment to first disease progression (1.5 years) and time from the most recent relapse to liso-cel infusion (0.3 years). The median age of our study population was consistent with that reported for phase 2 studies of axicabtagene ciloleucel (axi-cel), tisagenlecleucel and mosunetuzumab in patients with 3L+ FL^11,12,17^.

In this primary analysis, primary and key secondary endpoints were met, and similar efficacy was observed across lines of therapy. Liso-cel demonstrated remarkable efficacy in patients with 3L+ R/R FL, with a very high ORR (97%) and almost all responders achieving CR (94%). In patients with 2L FL who were eligible only if they met POD24 from initial immunochemotherapy (65%) or mGELF criteria (70%), ORR was similarly high at 96%, with all responders achieving CR. Responses were rapid and durable, with median time to response of 1 month and median DOR and PFS NR at a median follow-up of approximately 17 months. ORR, CR rate and 12-month DOR and PFS rates were consistently high across all subgroups, including patients with POD24 from initial immunochemotherapy, with double-refractory disease, patients with high tumor burden based on mGELF criteria, patients with high-risk FLIPI and patients who received bridging therapy. Reconfirmation of PET/CT-positive measurable disease after the liso-cel manufacturing period was a requirement to proceed with lymphodepleting chemotherapy (LDC) and liso-cel infusion, including in patients who received optional bridging therapy for anti-cancer disease control during this period. For patients who received radiation therapy as bridging therapy, the presence of non-irradiated PET-positive lesions was required to continue to meet eligibility criteria. In this study, two patients reached CR after bridging therapy (one patient had PET-negative disease at the reassessment visit and was not treated with liso-cel, whereas the other patient relapsed, had measurable disease at the reassessment visit, received liso-cel and was in ongoing CR as of data cutoff). In subgroup analyses, efficacy outcomes were similar regardless of bridging therapy status. Although the observed high response rates precluded detection of differences in subgroups, these results suggest that liso-cel treatment in the 3L+ and 2L settings can achieve high probabilities of continued response and survival without progression ≥1 year after infusion across broad patient subgroups, including those with poorer prognosis, such as POD24 from initial immunochemotherapy and double-refractory disease.

Although direct cross-trial comparisons of efficacy and safety cannot be made owing to differences in study design and definitions, some observations are worth mentioning that highlight the high efficacy and low toxicity profile of liso-cel. The ORR and CR rate achieved by liso-cel were consistent with those achieved by non-chemotherapy treatment strategies currently approved for 3L+ R/R FL. In phase 2, single-arm, registrational studies with the CD19-directed CAR T cell therapies axi-cel (ZUMA-5; EE, *n* = 86) and tisagenlecleucel (ELARA; EE, *n* = 94) and the CD20×CD3 bispecific antibody mosunetuzumab (GO29781; EE, *n* = 90; treatment included 8‒17 cycles), ORR ranged from 80% to 94%, and CR rates ranged from 60% to 79% (refs. ^11,12,17^). Median on-study follow-up after liso-cel treatment in the primary analysis of TRANSCEND FL was 18.9 months. In primary publications, the median on-study follow-ups were in the same range (24.4 months for axi-cel (updated analysis), 16.9 months for tisagenlecleucel and 18.3 months for mosunetuzumab). CR rates with liso-cel were the same for patients with versus without POD24 from initial immunochemotherapy (94% versus 94%). CR rates were numerically lower in patients with versus without POD24 from initial immunochemotherapy in studies of axi-cel (72% versus 83%) and tisagenlecleucel (59% versus 88%). The probability of PFS at 12 months was 83% for liso-cel. The 12-month PFS rates were 78% for axi-cel, 67% for tisagenlecleucel and 58% for mosunetuzumab. Three-year follow-up results for axi-cel and mosunetuzumab, and 2-year follow-up results for tisagenlecleucel, were consistent with the primary publications^18–20^. In two recent matching-adjusted indirect comparisons of mosunetuzumab and CAR T cell therapies, efficacy outcomes of ORR (liso-cel), CR rate (axi-cel and liso-cel) and PFS (axi-cel, liso-cel and tisagenlecleucel) favored CAR T cell therapies in patients with 3L+ FL, although data from randomized comparator trials or real-world studies are warranted to provide more conclusive results^21,22^.

Safety outcomes of liso-cel in R/R FL were consistent with previous studies of liso-cel in 2L and 3L+ R/R large B cell lymphoma^13–16^. Among liso-cel–treated patients with R/R FL, rates of severe CRS and NEs were low, with low tocilizumab/corticosteroid usage (25% for CRS; 7% for NEs), and no grade 4 or 5 events occurred. For liso-cel, rates of grade ≥3 CRS and NEs were 1% and 2%, respectively. For axi-cel^11^, tisagenlecleucel^12^, and mosunetuzumab^17,23^, respectively, rates of grade ≥3 CRS were 6%, 0% and 2%, and rates of grade ≥3 NEs were 15%, 3% and 3%.

We reported grade ≥3 infections in 5% of patients treated with liso-cel. In primary analyses of axi-cel, tisagenlecleucel and mosunetuzumab, grade ≥3 infections were reported in 15%, 5% and 14%, respectively. In ZUMA-5, prolonged high-grade cytopenias, defined as grade ≥3 cytopenias present on or after day 30 and before any subsequent anti-lymphoma therapy (assessed as TEAEs), were reported in 33% of patients with FL^11^. In TRANSCEND FL and ELARA, severe prolonged cytopenias were also reported per hematology laboratory data. Grade ≥3 laboratory-based neutropenia, anemia and thrombocytopenia, respectively, were reported in 15%, 5% and 15% of liso-cel–treated patients at day 29, with recovery to grade ≤2 in 90%, 83% and 58% by day 90. Of the 42% of patients with unresolved thrombocytopenia at day 90, all but one had thrombocytopenia at baseline (that is, pre-infusion), and 75% had recovered to their baseline platelet counts by the data cutoff. Similar results were reported in ELARA^12^.

The safety profile of liso-cel–treated patients in 2L and 3L+ FL was generally similar, with similar type, frequency and severity of AEs and no new safety signals identified in 2L FL. However, rates of severe infection (0% versus 7%), prolonged cytopenia incidence (13% versus 24%) and the proportion of patients treated with tocilizumab and/or corticosteroids to manage CRS/NEs (13% versus 31%) were numerically lower in 2L FL compared to 3L+ FL, although sample size disparity may have contributed to differences in safety observations between cohorts.

Among all liso-cel–treated patients, cellular kinetic analyses demonstrated liso-cel persistence through day 90 in over 60% of patients, with sustained persistence in ≥41% of patients through month 18. Although additional analyses are needed to determine the impact of cellular kinetics on clinical safety and efficacy, rates of CRS and NEs remained low, and median DOR was NR at the data cutoff, indicating that overall liso-cel exposure (AUC_0‒28d_) in our study was safe and conducive to clinical activity; exposure was similar to that of liso-cel in patients with large B cell lymphoma^13–16^. Patients with 2L FL showed higher maximum liso-cel expansion (with high variability among patients) but lower persistence than patients with 3L+ FL; however, larger patient populations and longer follow-up are required to appreciate any clinical consequences. Per B cell aplasia data in all liso-cel–treated patients, targeted depletion of CD19^+^ B cells was maintained over the timecourse analyzed, indicating sustained liso-cel functional activity. B cell aplasia incidence was high through day 90 (91‒99%) and steadily decreased thereafter, consistent with liso-cel persistence data.

We evaluated PROs using a dataset with high completion rates across assessments, representing, to our knowledge, the first extensive report of PROs from a clinical trial of patients with FL treated with CAR T cell therapy. These data, which included measures of quality of life, disease symptoms and functioning, showed improvement in PROs for most patients across 3L+ FL and 2L FL after liso-cel infusion, with improvements occurring as early as day 29 and within 3 months for most primary domains. Differences in demographic and disease characteristics at baseline between patients with 3L+ FL and 2L FL may have contributed to the faster median time to improvement across PRO domains observed with the 2L FL group. Patients with 3L+ FL versus 2L FL had slightly higher median age (62 years versus 53 years), longer median time from diagnosis to liso-cel infusion (5 years versus 2 years) and a higher proportion of patients with certain high-risk clinical features, such as FLIPI score 3‒5 (57% versus 35%), double-refractory disease status (64% versus 48%) and requirement for bridging therapy (41% versus 22%). Furthermore, the clinical data showed that patients with 2L FL versus 3L+ FL had slightly higher rates for 12-month DOR (90% versus 83%) and 12-month PFS (91% versus 81%), suggesting that the 2L FL group may have derived longer benefit and higher probability of progression-free disease that could have contributed to differences in PROs. However, observed differences should be interpreted with caution because the study was not designed to compare across cohorts. Overall, the PRO data characterize the direct patient experience with one-time liso-cel treatment in R/R FL and complement study outcomes demonstrating clinical benefit and manageable safety^11,12,17^.

This study has some limitations. Longer follow-up for DOR and PFS data are needed, as most patients were censored with ongoing response. This study has a single-arm design; however, this design was chosen because of the lack of established standard of care for patients with 3L+ FL and for those with 2L FL and high-risk disease features. The number of patients who could be monitored in an outpatient setting was affected by regulatory requirements in Europe and the coronavirus disease 2019 (COVID-19) pandemic.

In conclusion, patients with R/R FL enrolled in TRANSCEND FL represent a population without an established standard of care and with an unfavorable prognosis. Liso-cel demonstrated a meaningful benefit in patients with 2L+ R/R FL, including patients with 2L R/R FL with POD24 and/or other high-risk disease features, as supported by high response rates and durable responses. Liso-cel showed a favorable benefit/risk ratio in these patients, with low rates of severe CRS and NEs. Results from this study support liso-cel as a potential therapeutic option in patients with R/R FL, including 2L FL.

## Methods

### Study oversight

This study was conducted according to the Good Clinical Practice guidelines as described in the International Conference on Harmonization, ethical principles in the Declaration of Helsinki and applicable regulatory requirements. The institutional review board/ethics committee at participating institutions (Supplementary Appendix, page 4) reviewed and approved the study protocol and amendments. All patients provided written informed consent before any study procedures.

### Study design and participants

TRANSCEND FL (NCT04245839) is a global, phase 2, open-label, single-arm, multicohort, multicenter study evaluating efficacy and safety of liso-cel in patients with R/R iNHL. For FL cohorts, the study enrolled patients ≥18 years of age with histologically confirmed FL ≤6 months of screening as assessed by local pathology. All patients must have received one or more prior lines of combination systemic therapy with an anti-CD20 antibody and alkylator. Cohort assignment was as follows: fourth-line or later (4L+) FL and 3L cohort patients had received three or more and two prior lines of systemic therapy, respectively; 2L FL cohort patients had received one prior line of therapy and must have met POD24 criteria per protocol (that is, progressed ≤24 months of diagnosis and treated with an anti-CD20 antibody and alkylating agent ≤6 months of initial FL diagnosis) and/or ≥1 of the mGELF criteria (that is, symptoms attributable to FL, not limited to B symptoms; threatened end-organ function OR cytopenia secondary to lymphoma OR bulky disease (that is, for measurable nodal or extranodal lesions, single mass >7 cm or ≥3 masses >3 cm)); splenomegaly; and steady progression over ≥6 months (Supplementary Appendix, page 5)^5^.

Eligible patients underwent leukapheresis for liso-cel manufacturing. Treatment consisted of LDC (intravenous fludarabine 30 mg/m^2^/d and intravenous cyclophosphamide 300 mg/m^2^/d for 3 d) followed 2‒7 d later by a single liso-cel infusion at a total target dose of 100 × 10^6^ CAR^+^ T cells. Optional bridging therapy was allowed per treating investigator for disease control during liso-cel manufacturing and required reconfirmation of PET/CT-positive disease before LDC. Retreatment with liso-cel was not allowed. Liso-cel infusion and monitoring in the outpatient setting was allowed at the treating investigator’s discretion. Patients will be followed for safety, disease status and survival until 5 years after liso-cel infusion. Upon completion in TRANSCEND FL, all patients with FL treated with liso-cel would be asked to complete a signed informed consent form to enroll in a separate long-term follow-up (LTFU) study (NCT03435796) for up to 15 years after liso-cel infusion. In the LTFU study, patients would undergo assessments of safety and OS. A description of the trial design and eligibility criteria are provided in the Supplementary Appendix (pages 5‒9).

### Study endpoints

The primary endpoint was ORR per IRC by PET/CT per Lugano et al.^25^. Secondary efficacy endpoints were CR rate, DOR, DOR in patients with a BOR of CR, PFS and OS. PET/CT assessments were performed at screening, at days 29 and 90 and at months 6, 9, 12, 18, 24, 36, 48 and 60. Additional secondary endpoints were safety, cellular kinetics and PROs. Efficacy subgroup analyses (conducted for subgroups with ≥5 patients) and peripheral B cell aplasia were assessed as exploratory endpoints.

TEAEs were defined as an AE that started from liso-cel administration through and including 90 d after liso-cel infusion. AEs of special interest included infusion-related reaction, CRS, NEs (defined as investigator-identified neurological AEs related to liso-cel), MAS/HLH, tumor lysis syndrome (TLS), grade ≥3 infection, prolonged cytopenias (defined as grade ≥3 laboratory abnormalities of neutropenia, anemia or thrombocytopenia at day 29), hypogammaglobulinemia and second primary malignancy. Hypogammaglobulinemia and second primary malignancy could have occurred within or beyond the 90-d TEAE period.

Reporting of AEs was based on MedDRA and NCI CTCAE, version 5.0, with the exception of CRS, which was graded according to Lee et al.^24^ criteria, and TLS, which was graded according to Cairo and Bishop^26^. NEs were defined as investigator-identified neurological AEs related to liso-cel (captured on the AE domain of the electronic case report form (eCRF) using the preferred term ‘neurotoxicity’) and graded using NCI CTCAE, version 5.0, on the basis of the highest individual symptom grade. Symptoms of NEs were defined as investigator-identified events entered on the ‘Clinical Events − Neurotoxicity Details’ record in the eCRF from verbatim terms in patients who received liso-cel and for which the question ‘Is this event related to liso-cel?’ had been answered with ‘suspected’ on the neurotoxicity AE eCRF. Reporting of hypogammaglobulinemia was based on AEs that occurred on or after the liso-cel infusion date and were coded to the following MedDRA preferred terms: blood Ig A decreased, blood IgG decreased, blood IgM decreased, hypogammaglobulinemia, immunoglobulins decreased, selective IgA immunodeficiency, selective IgG subclass deficiency and selective IgM immunodeficiency. Reporting of second primary malignancy was based on findings from Standardized MedDRA Queries (SMQs) searches for ‘premalignant disorders’ and ‘malignancies’ and subsequent medical review by an internal adjudication panel. The process consisted of a review of preferred terms detected during an SMQs search of all reported AEs and selecting those deemed appropriate for inclusion as malignancies.

Cellular kinetic analyses of liso-cel in peripheral blood were performed in the cellular kinetic set, which included patients in the liso-cel–treated set with any available measurements of cellular kinetics by polymerase chain reaction. Concentration values after new anti-FL treatment were excluded from the summaries. B cell aplasia was defined as CD19^+^ B cells representing less than 3% of peripheral blood lymphocytes as measured by flow cytometry.

PROs were evaluated in the PRO analysis set (that is, patients in the liso-cel–treated set who completed a pre-LDC baseline visit and ≥1 post-baseline PRO measurement) using the EORTC QLQ-C30, FACT-LymS and EQ-5D-5L (health utility index and visual analog scale). We present results for primary domains of interest pre-selected for relevance to FL, which included six EORTC QLQ-C30 domains (global health status/quality of life, physical functioning, role functioning, cognitive functioning, fatigue and pain) and the FACT-LymS subscale assessing lymphoma-specific symptoms. Results for the EORTC QLQ-C30 secondary domains are also presented. The completion rates, defined as the number of patients submitting a valid PRO assessment at a given timepoint over the number of patients who are still expected to submit a PRO assessment at that timepoint, were calculated for all PRO questionnaires. At the group level, PROs were analyzed based on the mean changes from baseline at each study visit. Per Supplementary Table 20, minimally clinically important differences were based on published thresholds^27,28^. Additional PRO assessments conducted as post hoc analyses included a linear mixed-effects model for repeated measures to estimate least squares mean changes from baseline for primary and secondary domains, time to confirmed improvement for primary domains based on Kaplan–Meier methodology and proportions of patients with clinically meaningful improvement or deterioration from baseline for primary domains based on individual-level descriptive analyses.

### Statistical analyses

Data collection was performed using the Bristol Myers Squibb Rave Electronic Data Capture platform. Hierarchical hypothesis testing was used to control type I error across lines of therapy (4L+ FL, 3L+ FL (4L+ and 3L FL cohorts) and 2L FL) and endpoints (ORR and CR rate) at a one-sided α level of 0.025 (Supplementary Fig. 18). Assuming a 20% dropout rate, a planned sample size of approximately 138 patients with iNHL would ensure that approximately 110 patients with FL (4L+, *n* = 50; 3L, *n* = 40; 2L, *n* = 20) were treated with liso-cel. With this sample size, using exact binomial one-sample tests unadjusted for the hierarchical testing procedure, there would be 90% power in 4L+ FL and 3L+ FL and 80% power in 2L to detect improvements in ORR and CR rate endpoints versus defined thresholds (Supplementary Fig. 18). Specifically, for patients with 4L+ FL, the null hypotheses were ≤50% for ORR and ≤20% for CR rate, as assessed by PET/CT. With a sample size of 50 treated patients, using one-sided 0.025 level testing, there would be 90% power to detect an ORR of 74% versus 50% or a CR rate of 42% versus 20%. For patients with 3L+ FL (that is, 4L+ FL and 3L FL), the null hypotheses were ≤60% for ORR and ≤30% for CR rate. With a sample size of 90 patients, using one-sided 0.025 level testing, there would be 90% power to detect an ORR of 77% versus 60% or a CR rate of 48% versus 30%. For patients with 2L FL, the null hypotheses were ≤50% for ORR and ≤19% for CR rate. The analysis of this cohort was for proof of concept. With a sample size of 20 treated patients, using one-sided 0.025 level testing, there would be 80% power to detect an ORR of 80% versus 50% or a CR rate of 50% versus 19%. Efficacy and safety results in 2L+ FL were reported with descriptive statistics with no predefined testing hypothesis.

Safety was assessed in all liso-cel–treated patients (2L+ FL; liso-cel‒treated set). Efficacy was evaluated in the efficacy set (all patients in the liso-cel–treated set who had PET/CT-positive disease per IRC before liso-cel administration) and reported by lines of therapy. Patients without a repeat baseline assessment after bridging therapy and before liso-cel administration were excluded from the efficacy set. Further study population details are provided in Supplementary Table 21.

Time-to-event endpoints were summarized with medians and 95% CIs using the Kaplan–Meier method. For DOR and PFS, patients without documented PD or death were censored at the date of the last adequate disease assessment. For assessment of OS, data from surviving patients were censored at the last time that the patient was known to be alive. Sensitivity analysis of efficacy was performed in leukapheresed patients (that is, the ITT set).

### Reporting summary

Further information on research design is available in the Nature Portfolio Reporting Summary linked to this article.

## Online content

Any methods, additional references, Nature Portfolio reporting summaries, source data, extended data, supplementary information, acknowledgements, peer review information; details of author contributions and competing interests; and statements of data and code availability are available at 10.1038/s41591-024-02986-9.

### Supplementary information

Supplementary Information Study sites, Eligibility criteria, Study design and endpoints, Supplementary Tables 1–21, Supplementary Figs. 1–18 and References.

Reporting Summary

## Data Availability

In-scope proposals are sent to an independent review committee (IRC) to review and provide the final decision on the requests. Bristol-Myers Squibb has established a relationship with Duke University through the Duke Clinical Research Institute (DCRI) to act as that IRC. The IRC ensures that qualifying requests for patient-level data have a complete, consistent and fair assessment. They also review the proposal with the research team and discuss any clarifying questions that would better support the decision on the proposal. The IRC membership represents three broadly defined areas of expertise: clinical, statistical and bioethical/protection of human subjects. They also contract with additional experts depending on the request, therapeutic area or other relevant factors. DCRI will evaluate proposals based on scientific rationale and methodology, experience and relevant qualifications of the research team, presence of a robust statistical analysis plan and publication plan. No potential conflicts of interest exist. If conflicts of interest are present, there is a plan to address them. Before data being released, the researcher(s) will be expected to sign the Vivli Data Use Agreement. Upon execution of an agreement, the de-identified and/or anonymized datasets will be available within the Vivli Research environment. The Bristol-Myers Squibb policy on data sharing may be found at https://www.bms.com/researchers-and-partners/independent-research/data-sharing-request-process.html.
