# Author Correction: Lisocabtagene maraleucel in follicular lymphoma: the phase 2 TRANSCEND FL study

**DOI:** 10.1038/s41591-024-03175-4

**Published:** 2024-07-09

**Authors:** Franck Morschhauser, Saurabh Dahiya, M. Lia Palomba, Alejandro Martin Garcia-Sancho, Juan Luis Reguera Ortega, John Kuruvilla, Ulrich Jäger, Guillaume Cartron, Koji Izutsu, Martin Dreyling, Brad Kahl, Hervé Ghesquieres, Kirit Ardeshna, Hideki Goto, Anna Maria Barbui, Jeremy S. Abramson, Peter Borchmann, Isabelle Fleury, Stephan Mielke, Alan Skarbnik, Sven de Vos, Manali Kamdar, Reem Karmali, Andreas Viardot, Thalia Farazi, Omotayo Fasan, James Lymp, Min Vedal, Rina Nishii, Ariel Avilion, Jessica Papuga, Jinender Kumar, Loretta J. Nastoupil

**Affiliations:** 1 Centre Hospitalier Universitaire de Lille, Groupe de Recherche sur les formes Injectables et les Technologies Associées, Lille, France; 2 Stanford University School of Medicine, Stanford, CA USA; 3 University of Maryland Greenebaum Comprehensive Cancer Center, Baltimore, MD USA; 4 Memorial Sloan Kettering Cancer Center, New York, NY USA; 5 Hospital Universitario de Salamanca, IBSAL, CIBERONC, Centro de Investigación del Cáncer-IBMCC (USAL-CSIC), Salamanca, Spain; 6 Hospital Virgen del Rocío, Instituto de Biomedicina de la Universidad de Sevilla, Seville, Spain; 7 Princess Margaret Cancer Centre, Toronto, Ontario Canada; 8 Medical University of Vienna, Vienna, Austria; 9 Montpellier University Hospital Center, UMR CNRS 5535, Montpellier, France; 10 National Cancer Center Hospital, Tokyo, Japan; 11 LMU University Hospital, München, Germany; 12 Washington University School of Medicine in St. Louis, St. Louis, MO USA; 13 Hôpital Lyon Sud, Lyon, France; 14 University College London Hospitals Biomedical Research Centre, London, UK; 15 Hokkaido University Hospital, Sapporo, Japan; 16 Azienda Socio Sanitaria Territoriale Papa Giovanni XXIII, Bergamo, Italy; 17 Massachusetts General Hospital Cancer Center, Harvard Medical School, Boston, MA USA; 18 Universität zu Köln, Köln, Germany; 19 Hôpital Maisonneuve – Rosemont, Montreal, Quebec Canada; 20 Karolinska Institutet and University Hospital, Karolinska Comprehensive Cancer Center, Karolinska ATMP Center, Stockholm, Sweden; 21 Novant Health Cancer Institute, Charlotte, NC USA; 22 UCLA Santa Monica Medical Centre, Santa Monica, CA USA; 23 University of Colorado Cancer Center, Aurora, CO USA; 24 Northwestern University Feinberg School of Medicine, Robert H. Lurie Comprehensive Cancer Center, Chicago, IL USA; 25 Department of Internal Medicine III, University Hospital, Ulm, Germany; 26 Bristol Myers Squibb, Brisbane, CA USA; 27 Bristol Myers Squibb, Princeton, NJ USA; 28 Bristol Myers Squibb, Seattle, WA USA; 29 Bristol Myers Squibb, Boudry, Switzerland; 30 The University of Texas MD Anderson Cancer Center, Houston, TX USA

**Keywords:** B-cell lymphoma, Medical research

Correction to: *Nature Medicine* 10.1038/s41591-024-02986-9, published online 3 June 2024.

In the version of the article initially published, in the Japan row of Supplementary Fig. 2, the values for the columns “Evaluable patients” and “Patients with ORR” were originally both 8 but have now been amended to 9. A corrected Supplementary Information file has now been uploaded online.

