# Supplementary material for: Lisocabtagene maraleucel in follicular lymphoma: the phase 2 TRANSCEND FL study
Source: Nat Med. 2024 Jun 3;30(8):2199–207. doi: 10.1038/s41591-024-02986-9 (PMC11333271; doi:10.1038/s41591-024-02986-9)
Supplement: Supplementary file 1 — Study sites, Eligibility criteria, Study design and endpoints, Supplementary Tables 1–21, Supplementary Figs. 1–18 and References. [file 41591_2024_2986_MOESM1_ESM.pdf]

nature medicine

Article https://doi.org/10.1038/s41591-024-02986-9

Lisocabtagene maraleucel in follicular lymphoma: the phase 2 TRANSCEND FL study

In the format provided by the authors and unedited

Table of contents

Study sites.. 4
Eligibility criteria... 5
Study design and endpoints 10
Supplementary Table 1 | Manufacturing summary (liso-cel–treated set)................................12
Supplementary Table 2 | Demographic and baseline characteristics (liso-cel–treated set)[a]...13
Supplementary Table 3 | Patient-level characteristics of patients with 2L FL 16
Supplementary Table 4 | Anticancer (bridging) regimens for disease control[a]........................18
Supplementary Table 5 | ORR by best overall response per IRC assessment (efficacy set)..20
Supplementary Table 6 | Patient characteristics for nonresponders (efficacy set) 21
Supplementary Table 7 | ORR, DOR, PFS, and OS per IRC assessment (leukapheresed ITT set)...22
Supplementary Table 8 | ORR by best overall response per investigator assessment (efficacy set)...24
Supplementary Table 9 | Most common TEAEs[a] (≥10% in 2L+) (liso-cel–treated set) 25
Supplementary Table 10 | Grade 3 NEs (liso-cel–treated set) 26
Supplementary Table 11 | Treatment-emergent NE signs and symptoms (liso-cel–treated set)[a]..27
Supplementary Table 12. TEAEs of nervous system/psychiatric disorders regardless of attribution to liso-cel (liso-cel–treated set)[a] 29
Supplementary Table 13 | Grade 3 infections after 90-day treatment-emergent period (liso-cel–treated set)[a] 31
Supplementary Table 14 | Deaths (leukapheresed ITT set)..32
Supplementary Table 15 | ICU length of stay and AEs 34
Supplementary Table 16 | Summary of safety after liso-cel administration in patients monitored in the outpatient setting (outpatient analysis set)[a] 35
Supplementary Table 17 | Liso-cel cellular kinetic parameters (cellular kinetic set[a,b]) 36
Supplementary Table 18 | Liso-cel transgene persistence[a] (cellular kinetic set[b]) 37
Supplementary Table 19 | B-cell aplasia[a] (liso-cel–treated set[b]) 38
Supplementary Table 20 | Overall least square mean changes from baseline (PRO analysis set)[a]..39
Supplementary Table 21 | Study population definitions 40
Supplementary Fig. 1 | Forest plot of ORR per IRC assessment in 3L+ (efficacy set)............41
Supplementary Fig. 2 | Forest plot of ORR per IRC assessment in 2L+ (efficacy set)............42
Supplementary Fig. 3 | Forest plot of CRR per IRC assessment in 3L+ (efficacy set)............43

Supplementary Fig. 4 | Forest plot of CRR per IRC assessment in 2L+ (efficacy set). 44
Supplementary Fig. 5 | Forest plot of 12-month continued response rate (DOR) per IRC assessment in 3L+ FL (efficacy set). 45
Supplementary Fig. 6 | Forest plot of 12-month continued response rate (DOR) per IRC assessment in 2L+ FL (efficacy set). 46
Supplementary Fig. 7 | Forest plot of 12-month PFS rate per IRC assessment in 3L+ FL (efficacy set). 47
Supplementary Fig. 8 | Forest plot of 12-month PFS rate per IRC assessment in 2L+ FL (efficacy set). 48
Supplementary Fig. 9 | Liso-cel cellular kinetics over time in patients with 2L and 3L+ FL (cellular kinetic set[a])..49
Supplementary Fig. 10 | Completion rates for EORTC QLQ-C30 (PRO analysis set).[a] 50
Supplementary Fig. 11 | EORTC QLQ-C30 mean changes from baseline in the primary domains of interest over time (PRO analysis set).[a] 51
Supplementary Fig. 12 | EORTC QLQ-C30 mean changes from baseline in the secondary domains of interest over time (PRO analysis set).[a] 52
Supplementary Fig. 13 | FACT-LymS mean changes from baseline over time (PRO analysis set).[a] 53
Supplementary Fig. 14 | Time to confirmed improvement in primary domains of interest (PRO analysis set).[a] 54
Supplementary Fig. 15 | Time to confirmed improvement in FACT-LymS (PRO analysis set).[a] 55
Supplementary Fig. 16 | Proportion of patients with clinically meaningful change in the EORTC QLQ-C30 primary domains of interest (PRO analysis set).[a] 56
Supplementary Fig. 17 | Proportion of patients with clinically meaningful change in FACT-LymS (PRO analysis set).[a] 57
Supplementary Fig. 18 | Hierarchical hypothesis testing.[a] 58
References..59

Study sites

**Study site (*n*=31)**	**Country**
Medizinische Universitaet Wien	Austria
Hôpital Maisonneuve-Rosemont / Institut universitaire d'hématologie-oncologie et de thérapie cellulaire	Canada
Princess Margaret Cancer Centre	Canada
CHU Montpellier - Hôpital Saint Eloi	France
CHRU de Lille Hopital Claude Huriez; EFS Hauts de France Normandie	France
Centre Hospitalier Lyon Sud	France
University Hospital of Ulm; Institut fur klinische Transfusionsmedizin und Immungenetik Ulm gGmbH	Germany
Universitatsklinikum Koln; Klinik 1 fur Innere Medizin	Germany
U.O. Ematologia, Dipartimento di Oncologia ed Ematologia	Italy
National Cancer Center Hospital	Japan
Hokkaido University Hospital	Japan
Complejo Asistencial Universitario de Salamanca-Hospital Clinico Universitario de Salamanca	Spain
Hospital Universitario Virgen del Rocio; Centro Nacional de Aceleradores de Sevilla	Spain
Karolinska University Hospital Huddinge	Sweden
University College London Hospitals NHS Foundation Trust	United Kingdom
The Christie NHS Foundation Trust	United Kingdom
University of Maryland, Greenebaum Comprehensive Cancer Center	United States
The University of Texas MD Anderson Cancer Center	United States
Memorial Sloan Kettering Cancer Center	United States
Massachusetts General Hospital/Beth Israel Deaconess Medical Center - Dana-Farber Cancer Institute[a]	United States
Cleveland Clinic; Cleveland Clinic Taussig Cancer Institute	United States
University of Virginia Health System	United States
Northwestern University, Feinberg School of Medicine; Northwestern Memorial Hospital; Northwestern Medical Group	United States
University of Colorado Hospital	United States
Fred Hutchinson Cancer Research Center; Seattle Cancer Care Alliance; University of Washington Medical Center	United States
Novant Health Cancer Institute Hematology - Charlotte; Novant Health Presbyterian Medical Center; Novant Health Cancer Institute	United States
University of California, Los Angeles, Department of Medicine-Hematology/Oncology; University of California, Los Angeles, Hematology/Oncology-Santa Monica	United States
Yale University, Yale Cancer Center; Smilow Cancer Hospital at Yale-New Haven	United States
Providence Cancer Institute Franz Clinic; Providence Portland Medical Center; Providence Oncology and Hematology Care Clinic-Westside; Providence St.Vincent Medical Center; Providence Newberg Medical Center	United States
University of Pennsylvania, Abramson Cancer Center and Perelman Center for Advanced Medicine; Hospital of the University of Pennsylvania	United States

[a]The institution had two separate study sites and lead investigators.

Eligibility criteria

Inclusion criteria

Patients must satisfy the following criteria to be enrolled in the study:

1. Patient had follicular lymphoma (FL; grade 1, 2, or 3a) histologically confirmed within 6 months of screening, as assessed by local pathology. Availability of adequate archival tumor biopsy tissue from the last relapse, with corresponding pathology report for retrospective central pathology confirmation of diagnosis was required. If archival sample was before the last relapse or there was no tissue or insufficient tissue available, a new tumor biopsy was required. Tissue from fine needle aspirate was not permitted
2. Patient must have relapsed/refractory (R/R) disease, as assessed by the investigator
    a. Relapsed lymphoma was defined as relapse after an initial response of complete response (CR) or partial response to the prior therapy
    b. Refractory lymphoma was defined as a best response of stable disease or progressive disease (PD) after prior therapy
3. Patient must have measurable disease as follows:
    a. For patients with FL (fourth-line or later (4L+), third-line (3L), and second-line (2L) cohorts), positron emission tomography (PET)–positive disease with ≥1 PET-positive lesion and ≥1 measurable nodal or extranodal lesion according to the Celgene interpretation of the Lugano classification in two perpendicular dimensions. Nodal lesions >1.5 cm in the long axis regardless of the short axis and extranodal lesions >1.0 cm in the long and short axis on cross section imaging by computed tomography (CT) were considered measurable
4. Patient must have received the following, depending on cohort assignment:
    a. Cohort 1 (4L+ R/R FL): received ≥3 prior lines of systemic therapy. At least one of these lines must be a combination, which included an anti-CD20 antibody (e.g., rituximab, obinutuzumab) and an alkylating agent. Prior hematopoietic stem cell transplantation (HSCT) was permitted as a prior line of therapy. A group of 4L+ double-refractory patients were enrolled in this cohort
    b. Cohort 2 (3L R/R FL): received two prior lines of systemic therapy. At least one of these lines must be a combination, which included an anti-CD20 antibody (e.g., rituximab, obinutuzumab) and an alkylating agent. Prior HSCT was permitted as a prior line of therapy
    c. Cohort 3 (2L R/R FL): received one prior line of combination systemic therapy, which included an anti-CD20 antibody (e.g., rituximab, obinutuzumab) and an alkylating agent. A group of patients with progression of disease ≤24 months (POD24) of diagnosis, and had received treatment within 6 months of the original FL diagnosis, were enrolled to this cohort. The 2L patients

who did not meet this POD24 definition must instead have met one of the modified Groupe d'Etude des Lymphomes Folliculaires (mGELF) criteria, as outlined in inclusion criterion 5

5. Cohort 3 (2L R/R FL) patients must meet ≥1 criterion of the mGELF criteria listed below if they did not meet criteria of POD24 (having POD24 from diagnosis and must have received treatment for FL within 6 months of diagnosis):
    a. Symptoms attributable to FL (not limited to the symptoms noted below)
    b. Threatened end-organ function; OR cytopenia secondary to lymphoma; OR bulky disease (single mass >7 cm or ≥3 masses >3 cm)
    c. Splenomegaly
    d. Steady progression over ≥6 months
6. Patient was ≥18 years of age at the time of signing the informed consent form (ICF)
7. Patients who had received previous CD19-targeted therapy must have CD19-positive lymphoma confirmed on a biopsy since completing the prior CD19-targeted therapy
8. Patient had Eastern Cooperative Oncology Group performance status of 0 or 1
9. Patient had adequate organ function, defined as:
    a. Adequate bone marrow function to receive lymphodepleting chemotherapy (LDC), as assessed by the investigator
    b. Serum creatinine ≤1.5× age-adjusted upper limit of normal (ULN) OR calculated creatinine clearance (Cockcroft-Gault) >30 ml/min
    c. Alanine aminotransferase ≤5× ULN and total bilirubin <2.0 mg/dl (or <3.0 mg/dl for patients with Gilbert’s syndrome or lymphomatous infiltration of the liver)
    d. Adequate pulmonary function, defined as grade ≤1 dyspnea according to the National Cancer Institute Common Terminology Criteria for Adverse Events (NCI CTCAE), version 5.0, and oxygen saturation ≥92% on room air
    e. Adequate cardiac function, defined as left ventricular ejection fraction ≥40% as assessed by echocardiogram or multigated acquisition scan performed within 4 weeks of determination of eligibility
10. Patient had adequate vascular access for leukapheresis procedure
11. Patient must understand and voluntarily sign an ICF before any study-related assessments/procedures being conducted
12. Patient was willing and able to adhere to the study visit schedule and other protocol requirements
13. Patients must agree to not donate blood, organs, sperm or semen, and egg cells for usage in other individuals for ≥1 year after LDC. There were insufficient exposure data to provide any recommendation concerning the duration of refraining from tissue donation after treatment with lisocabtagene maraleucel (liso-cel). Therefore, patients treated with liso-cel should not donate blood, organs, tissues, and cells for transplantation
14. Females of childbearing potential (FCBP[a]) patients must:

a. Have had two negative pregnancy tests as verified by the investigator (one negative serum beta-human chorionic gonadotropin pregnancy test result at screening, and within 7 days before the first dose of LDC). This applies even if the patient practices true abstinence[b] from heterosexual contact
b. Have either committed to true abstinence[b] from heterosexual contact (which must be reviewed on a monthly basis and source documented) or agreed to use, and be able to comply with, effective contraception without interruption. Contraception methods must include one highly effective method from screening until ≥12 months after the LDC
c. Have agreed to abstain from breastfeeding during study participation and for ≥12 months after LDC
d. There were insufficient exposure data to provide any recommendation concerning the duration of contraception and the abstaining from breastfeeding after treatment with liso-cel. Any decision regarding contraception and breastfeeding after liso-cel infusion should be discussed with the treating physician
e. Note: highly effective methods were defined as those that result in a low failure rate (i.e., <1% per year) when used consistently and correctly. The following were examples of highly effective and additional effective methods of contraception:
    i. Intrauterine device
    ii. Hormonal (birth control pill, injections, implants)
    iii. Tubal ligation
    iv. Partner’s vasectomy

15. Male patients must:
    a. Practice true abstinence[b] (which must be reviewed on a monthly basis) or agree to use a condom during sexual contact with a pregnant female or a FCBP for 12 months after LDC even if he had undergone a successful vasectomy
    b. There were insufficient exposure data to provide any recommendation concerning the duration of contraception after treatment with liso-cel. Any decision regarding contraception after liso-cel infusion should be discussed with the treating physician

[a]A FCBP was a female who: 1) had achieved menarche at some point, 2) had not undergone a hysterectomy or bilateral oophorectomy, or 3) had not been naturally postmenopausal (amenorrhea after cancer therapy does not rule out childbearing potential) for ≥12 consecutive months (i.e., has had menses at any time in the preceding 12 consecutive months).

[b]True abstinence was acceptable when this was in line with the preferred and usual lifestyle of the patient. In contrast, periodic abstinence (e.g., calendar, ovulation, symptothermal, postovulation methods) and withdrawal were not acceptable methods of contraception.

Exclusion criteria

The presence of any of the following would exclude a patient from enrollment:

1. Evidence or history of composite diffuse large B-cell lymphoma and FL, or of transformed FL. Patients with World Health Organization subclassification of duodenal-type FL
2. Any significant medical condition, laboratory abnormality, or psychiatric illness that would prevent the patient from participating in the study based on the investigator’s judgment
3. Any condition, including the presence of laboratory abnormalities, which places the patient at unacceptable risk if he/she were to participate in the study based on the investigator’s judgment
4. Any condition that confounds the ability to interpret data from the study based on the investigator’s judgment
5. Central nervous system (CNS)—only involvement by malignancy (note: patients with secondary CNS involvement were allowed on study)
6. History of another primary malignancy that had not been in remission for ≥2 years, with the exception of the following noninvasive malignancies:
    a. Basal cell carcinoma of the skin
    b. Squamous cell carcinoma of the skin
    c. Carcinoma in situ of the cervix
    d. Carcinoma in situ of the breast
    e. Incidental histologic finding of prostate cancer (T1a or T1b using the TNM [tumor, nodes, metastasis] clinical staging system) or prostate cancer that was curative
    f. Other completely resected stage 1 solid tumor with low risk for recurrence
7. Previous treatment with alemtuzumab within 6 months of leukapheresis, or treatment with fludarabine or cladribine within 3 months of leukapheresis
8. Prior chimeric antigen receptor T-cell or other genetically modified cell therapy
9. History of active human immunodeficiency virus
10. Active hepatitis B or active hepatitis C. Patients with a negative polymerase chain reaction assay for viral load for hepatitis B or C were permitted. Patients positive for hepatitis B surface antigen and/or antihepatitis B core antibody with negative viral load were eligible and should be considered for prophylactic antiviral therapy
11. Uncontrolled systemic fungal, bacterial, viral, or other infection (including tuberculosis) despite appropriate antibiotics or other treatment
12. Active autoimmune disease requiring immunosuppressive therapy
13. Presence of acute or chronic graft-versus-host disease
14. History of any of the following cardiovascular conditions within the past 6 months before signing the ICF: Class III or IV heart failure as defined by the New York Heart Association, cardiac angioplasty or stenting, myocardial infarction, unstable angina, or other clinically significant cardiac disease

15. History or presence of clinically relevant CNS pathology not related to disease under study such as epilepsy, seizure, aphasia, stroke, cerebral edema, severe brain injuries, dementia, Parkinson’s disease, cerebellar disease, organic brain syndrome, or psychosis
16. Patient was a pregnant or nursing (lactating) woman
17. Patient had an intolerance to dimethyl sulfoxide and/or dextran
18. Progressive vascular tumor invasion, thrombosis, or embolism
19. Venous thrombosis or embolism not managed on a stable regimen of anticoagulation
20. Patient had received or undergone the following:
    a. Therapeutic doses of corticosteroids (defined as >20 mg/day prednisone or equivalent) within 7 days before unstimulated leukapheresis. Physiologic replacement, topical, and inhaled steroids were permitted
    b. Cytotoxic chemotherapeutic agents that were not considered lymphotoxic (see below) and intrathecal chemotherapy must be stopped ≥7 days before unstimulated leukapheresis
    c. Lymphotoxic chemotherapeutic agents (e.g., cyclophosphamide >300 mg/m^2, ifosfamide, bendamustine) 2 weeks before unstimulated leukapheresis
    d. Experimental agents within 4 weeks before signing the ICF unless no response or PD was documented on the experimental therapy and ≥3 half-lives have elapsed before unstimulated leukapheresis
    e. Ibrutinib, lenalidomide, and phosphoinositide 3-kinase inhibitor within 3 half-lives before unstimulated leukapheresis
    f. Immunosuppressive therapies within 4 weeks before leukapheresis and liso-cel infusion (e.g., calcineurin inhibitors, methotrexate or other chemotherapeutics, mycophenolate, rapamycin, thalidomide, immunosuppressive antibodies such as antitumor necrosis factor, anti-interleukin [IL] 6, or anti-IL6R)
    g. Donor lymphocyte infusion within 6 weeks before liso-cel infusion
    h. Radiation within 6 weeks of leukapheresis. Patient must have PD in irradiated lesions or have additional nonirradiated, PET-positive lesions to be eligible. Radiation to a single lesion, if additional nonirradiated PET-positive lesions were present, was allowed up to 2 weeks before unstimulated leukapheresis
    i. Systemic immunostimulatory agents (including but not limited to interferon and IL2) within 6 weeks or 5 half-lives of the drug, whichever was shorter, before liso-cel infusion
    j. Allogeneic HSCT within 90 days of leukapheresis

Study design and endpoints

Endpoint type	Endpoint	Description
Primary	ORR	Percentage of patients with a BOR[a] of CR or PR up to EOS[b]
Secondary	CR rate	Percentage of patients achieving CR at any time on-study up to EOS[b]
	Duration of CR	Time from first response (CR or PR) to PD or death from any cause, whichever occurs first, in patients with BOR of CR up to EOS[b]
	DOR	Time from first response (CR or PR) to PD or death from any cause, whichever occurs first, up to EOS[b]
	PFS	Time from liso-cel infusion to PD or death from any cause, whichever occurs first, up to EOS[b]
	OS	Time from liso-cel infusion to death from any cause up to EOS[b]
	Safety	Type, frequency, and severity of AEs and laboratory abnormalities, up to EOS[b]
	Cellular kinetics[c]	C_{max}, t_{max}, AUC, and persistence of liso-cel as assessed by PCR, up to EOS[b]
	PRO/HRQOL	Primary domains of interest assessed by EORTC QLQ-C30 (global health status/QOL, physical functioning, cognitive functioning, role functioning, fatigue, pain) and FACT-LymS up to 24 months after liso-cel infusion
Exploratory	Efficacy subgroup analyses	ORR, CR rate, DOR and PFS (defined by 12-month estimates of continued response rate and PFS rate), in patient subgroups by baseline demographic variables. Subgroup analyses were only performed if there were ≥5 patients in each subgroup
	B-cell aplasia[c]	Defined as <3% $CD19^+$ B cells in peripheral blood lymphocytes as assessed by flow cytometry

[a]PET/CT (CT was allowed if CR by PET with confirmatory bone marrow biopsy) assessments performed at screening, days 29 and 90, and months 6, 9, 12, 18, 24, 36, 48, and 60. [b]EOS, defined as up to 60 months after liso-cel infusion. [c]Peripheral blood samples were collected for cellular kinetics and B-cell aplasia assessments. For cellular kinetics: before LDC, day 1 (before liso-cel infusion), on days 4, 8, 11, 15, 22, 29, 60, and 90, and months 6, 9, 12, 18, 24, 30, 36, 42, 48, 54, and 60. For B-cell aplasia: before LDC, day 1 (before liso-cel infusion), and on days 8, 15, 22, 29, 60, and 90, and months 6, 9, 12, 18, 24, 36, 48, and 60.
AE, adverse event; AUC, area under the curve; BOR, best overall response; C_{max}, peak transgene level after liso-cel infusion; CR, complete response; DOR,

duration of response; EORTC QLQ-C30, European Organisation for Research and Treatment of Cancer Quality of Life Questionnaire-Core 30 items; EOS, end of study; FACT-LymS, Functional Assessment of Cancer Therapy-Lymphoma “Additional Concerns” Scale; HRQOL, health-related quality of life; LDC, lymphodepleting chemotherapy; liso-cel, lisocabtagene maraleucel; ORR, overall response rate; OS, overall survival; PCR, polymerase chain reaction; PD, progressive disease; PFS, progression-free survival; PR, partial response; PRO, patient-reported outcome; QOL, quality of life; t_{max}, time from liso-cel infusion to peak transgene level.

Supplementary Table 1 | Manufacturing summary (liso-cel–treated set)

	Europe (*n*=73)	US/Canada (*n*=47)	Japan (*n*=10)	3L+ FL (*n*=107)	2L FL (*n*=23)	Total (*N*=130)
Median time from leukapheresis to liso-cel availability (IQR), days	30 (29–33)	24 (22–28)	27.5 (27–29)	29 (26–31)	28 (25–29.5)	29 (25–31)
Median time from leukapheresis to infusion (IQR), days	52 (49–59)	38 (36–43)	48.5 (43–54)	50 (42–56)	48 (37.5–51.5)	49 (41–55)
Median time from liso-cel availability to infusion (IQR), days	22 (15–30)	15 (11–21)	21 (14–28)	20 (14–26)	19 (13.5–26.5)	20 (14–26)

IQR, interquartile range; liso-cel, lisocabtagene maraleucel; US, United States.

Supplementary Table 2 | Demographic and baseline characteristics (liso-cel–treated set)[a]

	**4L+ FL (*n*=59)**	**3L FL (*n*=48)**	**3L+ FL (*n*=107)**	**2L FL (*n*=23)**	**2L+ FL (*n*=130)**
Median age (range), years	64 (23–80)	60 (27–78)	62 (23–80)	53 (34–69)	60 (23–80)
Male sex (biological attribute), *n* (%)	35 (59)	31 (65)	66 (62)	17 (74)	83 (64)
Ethnicity, *n* (%)					
Hispanic or Latino	4 (7)	1 (2)	5 (5)	1 (4)	6 (5)
Not Hispanic or Latino	39 (66)	35 (73)	74 (69)	15 (65)	89 (68)
Not reported	16 (27)	12 (25)	28 (26)	7 (30)	35 (27)
Primary race, *n* (%)					
Asian	7 (12)	3 (6)	10 (9)	2 (9)	12 (9)
Black or African American	3 (5)	0	3 (3)	1 (4)	4 (3)
White	31 (53)	29 (60)	60 (56)	9 (39)	69 (53)
Not collected or unknown	18 (31)	16 (33)	34 (32)	11 (48)	45 (35)
Region, *n* (%)					
Europe	27 (46)	34 (71)	61 (57)	12 (52)	73 (56)
Japan	6 (10)	3 (6)	9 (8)	1 (4)	10 (8)
North America	26 (44)	11 (23)	37 (35)	10 (43)	47 (36)
ECOG PS at screening, *n* (%)					
0	30 (51)	35 (73)	65 (61)	17 (74)	82 (63)
1	29 (49)	13 (27)	42 (39)	6 (26)	48 (37)
FL subtype/grade at screening, *n* (%)					
Grade 1	3 (5)	6 (12.5)	9 (8)	6 (26)	15 (12)
Grade 2	41 (69)	31 (65)	72 (67)	11 (48)	83 (64)
Grade 3a	15 (25)	10 (21)	25 (23)	6 (26)	31 (24)
Unknown	0	1 (2)	1 (1)	0	1 (1)
Ann Arbor stage at screening, *n* (%)					
Stage I	1 (2)	0	1 (1)	1 (4)	2 (2)
Stage II	4 (7)	7 (15)	11 (10)	5 (22)	16 (12)
Stage III	18 (31)	21 (44)	39 (36)	6 (26)	45 (35)

	4L+ FL (*n*=59)	3L FL (*n*=48)	3L+ FL (*n*=107)	2L FL (*n*=23)	2L+ FL (*n*=130)
Stage IV	36 (61)	20 (42)	56 (52)	11 (48)	67 (52)
FLIPI at screening, *n* (%)					
Low risk (0–1)	9 (15)	3 (6)	12 (11)	11 (48)	23 (18)
Intermediate risk (2)	14 (24)	20 (42)	34 (32)	4 (17)	38 (29)
High risk (3–5)	36 (61)	25 (52)	61 (57)	8 (35)	69 (53)
SPD ≥50 cm^2 before LDC per IRC, *n* (%)	8 (14)	14 (29)	22 (21)	3 (13)	25 (19)
LDH > ULN before LDC, *n* (%)	27 (46)	20 (42)	47 (44)	6 (26)	53 (41)
mGELF criteria[b] met at time of most recent relapse, *n* (%)	31 (53)	26 (54)	57 (53)	16 (70)	73 (56)
Symptoms attributable to FL	8 (14)	5 (10)	13 (12)	6 (26)	19 (15)
Threatened end-organ function or cytopenia secondary to lymphoma or bulky disease	12 (20)	12 (25)	24 (22)	7 (30)	31 (24)
Splenomegaly	1 (2)	3 (6)	4 (4)	0	4 (3)
Steady progression over ≥6 mo	10 (17)	6 (12.5)	16 (15)	3 (13)	19 (15)
Median prior lines of systemic therapy (range)	4 (3–10)	2 (2–2)	3 (2–10)	1 (1–1)	2 (1–10)
Prior HSCT[c], *n* (%)	16 (27)	17 (35)	33 (31)	0	33 (25)
Received prior rituximab and lenalidomide, *n* (%)	18 (31)	5 (10)	23 (21)	0	23 (18)
Received prior bendamustine use at baseline, *n* (%)					
No prior bendamustine use	18 (31)	24 (50)	42 (39)	17 (74)	59 (45)
Prior bendamustine ≤6 mo before date of leukapheresis	2 (3)	2 (4)	4 (4)	1 (4)	5 (4)
Prior bendamustine >6 and ≤12 mo before date of leukapheresis	2 (3)	2 (4)	4 (4)	2 (9)	6 (5)
Prior bendamustine >12 mo before date of leukapheresis	37 (63)	20 (42)	57 (53)	3 (13)	60 (46)
Refractory to systemic therapy[d], *n* (%)	24 (41)	14 (29)	38 (36)	3 (13)	41 (32)
PD while on the last LOT or ≤6 mo of completing the last LOT, *n* (%)	44 (75)	25 (52)	69 (64)	15 (65)	84 (65)
SD or PD while on the last LOT or ≤6 mo of completing the last LOT, *n* (%)	45 (76)	27 (56)	72 (67)	15 (65)	87 (67)
POD24 from diagnosis, *n* (%)	21 (36)	25 (52)	46 (43)	12 (52)	58 (45)
FL progression ≤24 mo of first-line therapy with anti-CD20 antibody and alkylator, *n* (%)	27 (46)	31 (65)	58 (54)	15 (65)	73 (56)
Double refractory (anti-CD20 and alkylator), *n* (%)	36 (61)	33 (69)	69 (64)	11 (48)	80 (62)
Median time-to-event analyses (range)					

	4L+ FL (*n*=59)	3L FL (*n*=48)	3L+ FL (*n*=107)	2L FL (*n*=23)	2L+ FL (*n*=130)
Time from diagnosis to first PD, mo	24 (3–108)	24 (4–204)	24 (3–204)	24 (6–132)	24 (3–204)
Time from initial treatment to first PD, mo	20 (1–106)	16 (3–60)	18 (1–106)	16 (4–134)	18 (1–134)
Time from completion of last LOT to SD or PD[e], mo	1 (0–115)	3 (0–44)	2 (0–115)	4 (0–106)	2 (0–115)
Time from diagnosis to liso-cel infusion, mo	87 (22–423)	55 (8–225)	62 (8–423)	24 (9–137)	57 (8–423)
Time from most recent relapse to liso-cel infusion, mo	4 (0.4–38)	5 (2–24)	4 (0.4–38)	4 (1.7–16)	4 (0.4–38)
Received bridging therapy, *n* (%)	29 (49)	15 (31)	44 (41)	5 (22)	49 (38)

[a]Percentages may not add up to 100% due to rounding. All percentages are rounded to whole numbers except those with ".5%". [b]mGELF criteria (i.e., symptoms attributable to FL, not limited to B symptoms; threatened end-organ function OR cytopenia secondary to lymphoma OR bulky disease (i.e., for measurable nodal or extranodal lesions, single mass >7 cm or ≥3 masses >3 cm); splenomegaly; steady progression over ≥6 months). [c]All prior HSCT was autologous HSCT. [d]Defined as best response of SD or PD after prior therapy. If not refractory, then relapsed, defined as relapse after an initial response of complete response or partial response to the prior therapy. [e]Calculated by taking the day of progression (or SD if missing progression data) and subtracting the day of the last prior regimen completion. For patients who progressed before completion of their last prior line of anticancer therapy, the start date of range was set to 0. 2L, second line; 2L+, second line or later; 3L, third line; 3L+, third line or later; 4L+, fourth line or later; ECOG PS, Eastern Cooperative Oncology Group performance status; FL, follicular lymphoma; FLIPI, Follicular Lymphoma International Prognostic Index; HSCT, hematopoietic stem cell transplantation; IRC, independent review committee; LDC, lymphodepleting chemotherapy; LDH, lactate dehydrogenase; liso-cel, lisocabtagene maraleucel; LOT, line of therapy; mGELF, modified Groupe d'Etude des Lymphomes Folliculaires; PD, progressive disease; POD24, progression of disease ≤24 months; SD, stable disease; SPD, sum of the product of perpendicular diameters; ULN, upper limit of normal.

Supplementary Table 3 | Patient-level characteristics of patients with 2L FL

Patient	Prior systemic anticancer regimen	Number of cycles[a]	Best response	Time from initial FL treatment to first PD (months)	Time from completion of most recent systemic regimen to PD (months)	FL subtype/ grade at screening	Ann Arbor stage at screening	FLIPI at screening	SPD before LDC per IRC (cm^2)	LDH before LDC	mGELF at the time of most recent relapse (yes or no)	Specific mGELF criteria met
1	R-CHOP	6	CR	8.3	−4.0[b]	3A	III	Low (0–1)	15.7	<500 U/L ≤ULN	Yes	Symptoms attributable to FL
2	R-CHOP	6	PR	5.5	−1.0[b]	1	IV	High (3–5)	2.9	≥500 U/L ≤ULN	Yes	Threatened end-organ function; or cytopenia secondary to lymphoma; or bulky disease
3	O-CHOP	6	CR	9.5	−0.5[b]	2	II	Low (0–1)	16.3	<500 U/L >ULN	No	
4	O-CHOP	6	CR	16.0	−0.2[b]	3A	II	Low (0–1)	9.5	<500 U/L ≤ULN	No	
5	R-CHOP	6	CR	16.4	0.1	2	IV	Low (0–1)	2.7	<500 U/L ≤ULN	No	
6	BR	6	PR	19.2	0.6	1	III	High (3–5)	30.9	<500 U/L ≤ULN	Yes	Symptoms attributable to FL
7	R-CHOP	6	PR	7.6	1.0	3A	II	Low (0–1)	13.3	<500 U/L ≤ULN	Yes	Threatened end-organ function; or cytopenia secondary to lymphoma; or bulky disease
8	BR	3	PD	4.0	1.0	2	IV	Intermediate (2)	211.3	<500 U/L >ULN	Yes	Symptoms attributable to FL
9	R-CHOP	6	PR	6.3	1.1	2	IV	Low (0–1)	9.8	<500 U/L >ULN	No	
10	R-CHOP	14[c]	CR	19.5	1.6	2	II	Low (0–1)	38.2	<500 U/L ≤ULN	Yes	Threatened end-organ function; or cytopenia secondary to lymphoma; or bulky disease
11	R-CHOP	6	CR	33.0	1.6	1	III	Intermediate (2)	65.5	<500 U/L ≤ULN	Yes	Threatened end-organ function; or cytopenia secondary to lymphoma; or bulky disease
12	R-CHOP	6	PR	7.6	3.7	3A	IV	High (3–5)	16.0	<500 U/L ≤ULN	Yes	Steady progression over at least 6 months
13	R-CHOP	6	CR	10.8	4.9	3A	IV	High (3–5)	15.0	<500 U/L >ULN	No	
14	R-CHOP	6	CR	28.5	5.5	2	IV	Intermediate (2)	13.5	<500 U/L ≤ULN	Yes	Steady progression over at least 6 months

Patient	Prior systemic anticancer regimen	Number of cycles[a]	Best response	Time from initial FL treatment to first PD (months)	Time from completion of most recent systemic regimen to PD (months)	FL subtype/ grade at screening	Ann Arbor stage at screening	FLIPI at screening	SPD before LDC per IRC (cm^2)	LDH before LDC	mGELF at the time of most recent relapse (yes or no)	Specific mGELF criteria met
15	R-CHP	8	PR	37.4	5.6	2	IV	High (3–5)	49.7	<500 U/L ≤ULN	Yes	Threatened end-organ function; or cytopenia secondary to lymphoma; or bulky disease
16	BR	6	PR	11.0	6.1	2	I	Low (0–1)	9.8	<500 U/L ≤ULN	Yes	Steady progression over at least 6 months
17	BR	6	PR	11.6	7.0	2	III	Low (0–1)	26.5	<500 U/L ≤ULN	No	
18	BR	6	CR	17.9	12.9	3A	II	Low (0–1)	1.7	<500 U/L ≤ULN	No	
19	FCR	4	CR	46.3	14.3	1	IV	High (3–5)	7.5	<500 U/L ≤ULN	Yes	Symptoms attributable to FL
20	BR	6	CR	29.7	25.1	1	III	High (3–5)	47.1	<500 U/L >ULN	Yes	Threatened end-organ function; or cytopenia secondary to lymphoma; or bulky disease
21	R-CHOP	NA	PR	46.6	29.1	2	III	High (3–5)	36.0	<500 U/L ≤ULN	Yes	Symptoms attributable to FL
22	Chlorambucil-R	NA	CR	75.0	48.0	2	IV	Intermediate (2)	57.4	<500 U/L >ULN	Yes	Threatened end-organ function; or cytopenia secondary to lymphoma; or bulky disease
23	R-CHOP	6	CR	133.5	106.1	1	IV	Low (0–1)	6.4	<500 U/L ≤ULN	Yes	Symptoms attributable to FL

[a]Number of cycles reported (when available) reflects the number of cycles administered for the combination regimen. [b]Negative value was due to disease progression before regimen completion. [c]Per investigator, patient received 8 cycles of R-CHOP and 6 cycles of R maintenance. 2L, second line; BR, bendamustine plus rituximab; CR, complete response; FCR, fludarabine plus cyclophosphamide plus rituximab; FL, follicular lymphoma; FLIPI, Follicular Lymphoma International Prognostic Index; IRC, independent review committee; LDC, lymphodepleting chemotherapy; LDH, lactate dehydrogenase; mGELF, modified Groupe d'Etude des Lymphomes Folliculaires; NA, not available; O-CHOP, obinutuzumab plus cyclophosphamide, doxorubicin, vincristine, and prednisone; PD, progressive disease; PR, partial response; R, rituximab; R-CHP, rituximab plus cyclophosphamide, doxorubicin, and prednisone; R-CHOP, rituximab plus cyclophosphamide, doxorubicin, vincristine, and prednisone; SPD, sum of the product of perpendicular diameters; ULN, upper limit of normal.

Supplementary Table 4 | Anticancer (bridging) regimens for disease control[a]

	**2L+ FL (*n*=130)**
Received bridging therapy, *n* (%)	
Yes	49 (38)
No	81 (62)
Type of therapy, *n* (%)	
Systemic therapy only	44 (34)
Radiotherapy only	5 (4)
Both	0
Bridging regimens used,[b] *n* (%)	
PI3Ki[c]	7 (5)
R-GemOx	6 (5)
Bendamustine plus rituximab	3 (2)
R2	3 (2)
R-CVP	3 (2)
Bendamustine	2 (2)
Cyclophosphamide plus prednisone	2 (2)
CHOP	2 (2)
CVP	2 (2)
R-CHOP	2 (2)
VDC	2 (2)
Bendamustine plus prednisone	1 (1)
Bendamustine plus obinutuzumab	1 (1)
Carboplatin plus doxorubicin	1 (1)
GDP	1 (1)
DHAOx	1 (1)
D-GemOx	1 (1)
GemOx	1 (1)
Lenalidomide	1 (1)
Lenalidomide plus GemOx	1 (1)

	2L+ FL (*n*=130)
O-CHPol	1 (1)
O-CV	1 (1)
P-BTKi	1 (1)
Pol-CH	1 (1)
Rituximab	1 (1)
R-DHAOx	1 (1)
R-EPOH	1 (1)
R-GDP	1 (1)
R-GemCis	1 (1)

[a]Anticancer treatment for disease control was defined as any systemic therapy or radiation therapy provided to patients for disease control after leukapheresis and before LDC. One patient achieved CR after bridging therapy and relapsed. This patient had measurable disease at the pretreatment visit and was treated with liso-cel. The patient was in ongoing CR at the data cutoff.

[b]Of 54 leukapheresed patients who received bridging therapy, 49 patients received systemic therapy with a total of 52 systemic bridging regimens used to treat these patients; 2 patients had received 2 different combination therapies during the bridging period, accounting for the greater relative number of regimens to recipients.

[c]Includes single-regimen PI3Ki (*n*=4), PI3Ki in combination with chemotherapy (*n*=1), and PI3Ki plus anti-CD20 antibody (*n*=2). 2L+, second line or later; BTKi, Bruton tyrosine kinase inhibitor; CHOP, cyclophosphamide, doxorubicin, vincristine, and prednisone; CR, complete response; CVP, cyclophosphamide, vincristine, and prednisone; D-GemOx, dexamethasone plus gemcitabine and oxaliplatin; DHAOx, dexamethasone, cytarabine, and oxaliplatin; FL, follicular lymphoma; GDP, gemcitabine, dexamethasone, and cisplatin; GemOx, gemcitabine and oxaliplatin; LDC, lymphodepleting chemotherapy; liso-cel, lisocabtagene maraleucel; O, obinutuzumab; O-CHPol, obinutuzumab plus polatuzumab, cyclophosphamide, and doxorubicin; O-CV, obinutuzumab plus cyclophosphamide and vincristine; P-BTKi, prednisone plus BTKi; PI3Ki, phosphoinositide 3-kinase inhibitor; Pol-CH, polatuzumab, cyclophosphamide, and doxorubicin; R2, lenalidomide and rituximab; R-CHOP, rituximab plus cyclophosphamide, doxorubicin , vincristine, and prednisone; R-CVP, rituximab plus cyclophosphamide, vincristine, and prednisone; R-DHAOx, rituximab plus dexamethasone, cytarabine, and oxaliplatin; R-EPOH, rituximab plus etoposide, vincristine, doxorubicin, and prednisone; R-GDP, rituximab plus gemcitabine, dexamethasone, and cisplatin; R-GemCis, rituximab plus gemcitabine and cisplatin; VDC, vincristine, doxorubicin, and cyclophosphamide.

Supplementary Table 5 | ORR by best overall response per IRC assessment (efficacy set)

	**4L+ FL (*n*=53)**	**3L FL (*n*=48)**	**3L+ FL (*n*=101)**	**2L FL (*n*=23)**	**2L+ FL (*n*=124)**
ORR, *n* (%)	51 (96)	47 (98)	98 (97)	22 (96)	120 (97)
95% CI	87.0–99.5	88.9–99.9	91.6–99.4	78.1–99.9	91.9–99.1
One-sided *P* value	<0.0001 (H_0: ≤50%)	Not tested	<0.0001 (H_0: ≤60%)	<0.0001 (H_0: ≤50%)	Not tested
CR rate, *n* (%)	49 (92)	46 (96)	95 (94)	22 (96)	117 (94)
95% CI	81.8–97.9	85.7–99.5	87.5–97.8	78.1–99.9	88.7–97.7
One-sided *P* value	<0.0001 (H_0: ≤20%)	Not tested	<0.0001 (H_0: ≤30%)	<0.0001 (H_0: ≤19%)	Not tested
PR rate, *n* (%)	2 (4)	1 (2)	3 (3)	0	3 (2)
95% CI	0.5–13.0	0.1–11.1	0.6–8.4	0.0–14.8	0.5–6.9
SD, *n* (%)	1 (2)[a]	0	1 (1)	0	1 (1)
PD, *n* (%)	1 (2)[a]	0	1 (1)	1 (4)[a]	2 (2)
Not evaluable, *n* (%)	0	1 (2)	1 (1)	0	1 (1)

[a]Please refer to **Supplementary Table 7** for patient characteristics. 2L, second line; 2L+, second line or later; 3L, third line; 3L+, third line or later; 4L+, fourth line or later; CI, confidence interval; CR, complete response; FL, follicular lymphoma; H_0, null hypothesis; IRC, independent review committee; ORR, overall response rate; PD, progressive disease; PR, partial response; SD, stable disease.

Supplementary Table 6 | Patient characteristics for nonresponders (efficacy set)

	Patient 1	Patient 2	Patient 3[a]
Age, years	53	64	66
FL cohort	4L+	4L+	2L
Stage	IV	IV	IV
FLIPI	High risk	High risk	High risk
Relapsed/Refractory	Refractory[b]	Refractory[b]	Relapsed[c]
Time from completion of most recent systemic therapy to PD	PD before regimen completion	PD before regimen completion	PD during anti-CD20 antibody maintenance therapy after completing treatment with anti-CD20 antibody plus alkylator
mGELF	Yes	Yes	Yes
POD24 from diagnosis	No	No	Yes
Double refractory to anti-CD20 antibody and alkylator	Yes	Yes	Yes
Prior LOT	8	4	1
Bridging therapy	Yes (PI3Ki)	Yes (CHOP)	Yes (BR)
Baseline LDH	>ULN	<ULN	<ULN
SPD before LDC, cm^2	NA	<50	<50
C_{max}, copies/µg	12,412	18,442	121,758
$AUC_{(0–28d)}$, days*copies/µg	NA	181,145	NA

[a]Bone marrow was >90% lymphoma with bone lesions and pleural effusions at baseline. Before liso-cel treatment, the patient had pancytopenia and elevated ferritin based on laboratory assessment in peripheral blood. The patient experienced a grade 5 TEAE of MAS/HLH after liso-cel treatment. [b]Refractory disease was defined as a best response of SD or PD after prior therapy. [c]Relapsed disease was defined as relapse after an initial response of CR or PR to the prior therapy. 2L, second line; 4L+, fourth line or later; $AUC_{(0–28d)}$, area under the curve for liso-cel transgene levels from 0 to 28 days after infusion; BR, bendamustine plus rituximab; CHOP, cyclophosphamide, doxorubicin, vincristine, and prednisone; C_{max}, peak liso-cel transgene level after infusion; CR, complete response; FL, follicular lymphoma; FLIPI, Follicular Lymphoma International Prognostic Index; LDC, lymphodepleting chemotherapy; LDH, lactate dehydrogenase; liso-cel, lisocabtagene maraleucel; LOT, line of therapy; MAS/HLH, macrophage activation syndrome/hemophagocytic lymphohistiocytosis; mGELF, modified Groupe d'Etude des Lymphomes Folliculaires; NA, not available/evaluable; PI3Ki, phosphoinositide 3-kinase inhibitor; POD24, progression of disease ≤24 months; PD, progressive disease; PR, partial response; SD, stable disease; SPD, sum of the product of perpendicular diameters; TEAE, treatment-emergent adverse event; ULN, upper limit of normal.

Supplementary Table 7 | ORR, DOR, PFS, and OS per IRC assessment (leukapheresed ITT set)

**Overall response outcomes**	**4L+ FL (*n*=65)**	**3L FL (*n*=49)**	**3L+ FL (*n*=114)**	**2L FL (*n*=25)**	**2L+ FL (*n*=139)**
ORR, *n* (%)<br>95% CI	58 (89)<br>79.1–95.6	48 (98)<br>89.1–99.9	106 (93)<br>86.6–96.9	23 (92)<br>74.0–99.0	129 (93)<br>87.2–96.5
CR rate, *n* (%)<br>95% CI	56 (86)<br>75.3–93.5	47 (96)<br>86.0–99.5	103 (90)<br>83.4–95.1	23 (92)<br>74.0–99.0	126 (91)<br>84.5–94.9
PR rate, *n* (%)<br>95% CI	2 (3)<br>0.4–10.7	1 (2)<br>0.1–10.9	3 (3)<br>0.5–7.5	0<br>0.0–13.7	3 (2)<br>0.4–6.2
SD, *n* (%)	2 (3)	0	2 (2)	0	2 (1)
PD, *n* (%)	1 (2)	0	1 (1)	1 (4)	2 (1)
No evidence of disease, *n* (%)	1 (2)	0	1 (1)	0	1 (1)
Not evaluable, *n* (%)	3 (5)	1 (2)	4 (4)	1 (4)	5 (4)
**Time-to-event outcomes**					
	n=58	*n*=48	*n*=106	*n*=23	*n*=129
Median DOR, mo[a]<br>95% CI	NR<br>18.0–NR	NR<br>—	NR<br>18.0–NR	NR<br>19.3–NR	NR<br>19.3–NR
12-month DOR rate, %<br>95% CI	84<br>71.7–91.4	82<br>67.5–90.8	83<br>74.4–89.3	90<br>65.2–97.4	84.5<br>76.8–89.9
	n=65	*n*=49	*n*=114	*n*=25	*n*=139
Median PFS, mo<br>95% CI	NR<br>20.7–NR	NR<br>—	NR<br>20.7–NR	NR<br>21.4–NR	NR<br>21.4–NR
12-month PFS rate, %<br>95% CI	84<br>71.8–90.9	83<br>69.4–91.3	83.5<br>75.1–89.3	92<br>70.6–97.8	85<br>77.7–90.0
Median OS[b], mo<br>95% CI	NR<br>NR–NR	NR<br>NR–NR	NR<br>NR–NR	NR<br>NR–NR	NR<br>NR–NR
12-month OS rate[b], %<br>95% CI	92<br>82.1–96.6	94<br>82.1–98.0	93<br>86.2–96.4	96<br>73.9–99.4	93<br>87.7–96.5

[a]Based on Kaplan–Meier estimates of DOR. [b]A total of 90% of patients in the efficacy set (*n*=124) were censored from the analysis at the data cutoff. 2L, second line; 2L+, second line or later; 3L, third line; 3L+, third line or later; 4L+, fourth line or later; CI, confidence interval; CR, complete response; DOR, duration of response; FL, follicular lymphoma; IRC, independent review committee; ITT, intent-to-treat; NR, not reached; ORR,

overall response rate; OS, overall survival; PD, progressive disease; PFS, progression-free survival; PR, partial response; SD, stable disease; SE, standard error.

Supplementary Table 8 | ORR by best overall response per investigator assessment (efficacy set)

	**4L+ FL (*n*=53)**	**3L FL (*n*=48)**	**3L+ FL (*n*=101)**	**2L FL (*n*=23)**	**2L+ FL (*n*=124)**
ORR, *n* (%)	52 (98)	47 (98)	99 (98)	23 (100)	122 (98)
95% CI	89.9–100.0	88.9–99.9	93.0–99.8	85.2–100.0	94.3–99.8
CR rate, *n* (%)	49 (92)	46 (96)	95 (94)	22 (96)	117 (94)
95% CI	81.8–97.9	85.7–99.5	87.5–97.8	78.1–99.9	88.7–97.7
PR rate, *n* (%)	3 (6)	1 (2)	4 (4)	1 (4)	5 (4)
95% CI	1.2–15.7	0.1–11.1	1.1–9.8	0.1–21.9	1.3–9.2
SD, *n* (%)	1 (2)	0	1 (1)	0	1 (1)
PD, *n* (%)	0	0	0	0	0
Not evaluable, *n* (%)	0	1 (2)	1 (1)	0	1 (1)

2L, second line; 2L+, second line or later; 3L, third line; 3L+, third line or later; 4L+, fourth line or later; CI, confidence interval; CR, complete response; FL, follicular lymphoma; ORR, overall response rate; PD, progressive disease; PR, partial response; SD, stable disease.

Supplementary Table 9 | Most common TEAEs[a] (≥10% in 2L+) (liso-cel–treated set)

	**4L+ FL (*n*=59)**		**3L FL (*n*=48)**		**3L+ FL (*n*=107)**		**2L FL (*n*=23)**		**2L+ FL (*n*=130)**	
**TEAE, *n* (%)**	**Any grade**	**Grade ≥3**	**Any grade**	**Grade ≥3**	**Any grade**	**Grade ≥3**	**Any grade**	**Grade ≥3**	**Any grade**	**Grade ≥3**
Neutropenia	38 (64)	35 (59)	33 (69)	29 (60)	71 (66)	64 (60)	14 (61)	12 (52)	85 (65)	76 (58)
CRS	35 (59)	1 (2)	28 (58)	0	63 (59)	1 (1)	12 (52)	0	75 (58)	1 (1)
Anemia	25 (42)	6 (10)	19 (40)	6 (12.5)	44 (41)	12 (11)	5 (22)	1 (4)	49 (38)	13 (10)
Headache	19 (32)	0	11 (23)	0	30 (28)	0	8 (35)	0	38 (29)	0
Thrombocytopenia	18 (31)	4 (7)	12 (25)	8 (17)	30 (28)	12 (11)	3 (13)	1 (4)	33 (25)	13 (10)
Constipation	13 (22)	0	9 (19)	0	22 (21)	0	4 (17)	0	26 (20)	0
Pyrexia	10 (17)	0	11 (23)	0	21 (20)	0	2 (9)	0	23 (18)	0
Diarrhea	9 (15)	0	7 (15)	0	16 (15)	0	6 (26)	0	22 (17)	0
Lymphopenia	8 (14)	7 (12)	7 (15)	6 (12.5)	15 (14)	13 (12)	5 (22)	4 (17)	20 (15)	17 (13)
Fatigue	9 (15)	0	3 (6)	0	12 (11)	0	7 (30)	0	19 (15)	0
Tremor	9 (15)	0	7 (14)	0	16 (15)	0	2 (9)	0	18 (14)	0
Leukopenia	8 (14)	7 (12)	6 (12.5)	4 (8)	14 (13)	11 (10)	4 (17)	4 (17)	18 (14)	15 (12)
Asthenia	9 (15)	0	5 (10)	0	14 (13)	0	2 (9)	0	16 (12)	0

[a]TEAE period was defined as the time from initiation of liso-cel administration through and including study day 90. All percentages are rounded to whole numbers except those with ".5%". 2L, second line; 2L+, second line or later; 3L, third line; 3L+, third line or later; 4L+, fourth line or later; CRS, cytokine release syndrome; FL, follicular lymphoma; liso-cel, lisocabtagene maraleucel; TEAE, treatment-emergent adverse event.

Supplementary Table 10 | Grade 3 NEs (liso-cel–treated set)

	NE symptoms[a,b], days	Time to onset[c]	Time to resolution[c]
Patient 1	Grade 3 expressive aphasia (8)	13 days	17 days
	Grade 3 confusion (8)		
	Grade 2 expressive aphasia (10)		
Patient 2	Grade 2 expressive dysphasia (1)	11 days	1 day
	Grade 3 encephalopathy (1)		
Patient 3	Grade 3 irritability (1)	9 days	2 days
	Grade 3 reduced consciousness (1)		
	Grade 3 speech disturbance (1)		

[a]NE was defined as investigator-identified neurological AEs related to liso-cel. NE symptoms were defined as investigator-identified events entered on the “Clinical Events− Neurotoxicity Details” record in the eCRF from verbatim terms in patients who received liso-cel and for which the question, “Is this event related to liso-cel?,” had been answered with “suspected” on the neurotoxicity AE eCRF. [b]Grade 1 NE symptoms not reported. [c]Describes time to event for all grades of NE symptoms experienced. AE, adverse event; eCRF, electronic case report form; liso-cel, lisocabtagene maraleucel; NE, neurological event.

Supplementary Table 11 | Treatment-emergent NE signs and symptoms (liso-cel–treated set)[a]

	**4L+ FL (*n*=59)**		**3L FL (*n*=48)**		**3L+ FL (*n*=107)**		**2L FL (*n*=23)**		**2L+ FL (*n*=130)**	
***n* (%)**	**Any grade**	**Grade ≥3**	**Any grade**	**Grade ≥3**	**Any grade**	**Grade ≥3**	**Any grade**	**Grade ≥3**	**Any grade**	**Grade ≥3**
Aphasia	6 (10)	1 (2)	1 (2)	0	7 (7)	1 (1)	2 (9)	0	9 (7)	1 (1)
Tremor	4 (7)	0	4 (8)	0	8 (7)	0	1 (4)	0	9 (7)	0
Dyscalculia	2 (3)	0	1 (2)	0	3 (3)	0	0	0	3 (2)	0
Dysgraphia	1 (2)	0	2 (4)	0	3 (3)	0	0	0	3 (2)	0
Headache	0	0	3 (6)	0	3 (3)	0	0	0	3 (2)	0
Confusional state	2 (3)	1 (2)	0	0	2 (2)	1 (1)	0	0	2 (2)	1 (1)
Disorientation	1 (2)	0	1 (2)	0	2 (2)	0	0	0	2 (2)	0
Agitation	1 (2)	0	0	0	1 (1)	0	0	0	1 (1)	0
Asthenia	0	0	1 (2)	0	1 (1)	0	0	0	1 (1)	0
Cognitive disorder	1 (2)	0	0	0	1 (1)	0	0	0	1 (1)	0
Depressed level of consciousness	0	0	1 (2)	1 (2)	1 (1)	1 (1)	0	0	1 (1)	1 (1)
Disturbance in attention	1 (2)	0	0	0	1 (1)	0	0	0	1 (1)	0
Dysarthria	1 (2)	0	0	0	1 (1)	0	0	0	1 (1)	0
Electroencephalogram abnormal	0	0	0	0	0	0	1 (4)	0	1 (1)	0
Encephalopathy	0	0	0	0	0	0	1 (4)	1 (4)	1 (1)	1 (1)
Feeling abnormal	0	0	0	0	0	0	1 (4)	0	1 (1)	0
Fine motor skill dysfunction	0	0	1 (2)	0	1 (1)	0	0	0	1 (1)	0
Visual hallucination	1 (2)	0	0	0	1 (1)	0	0	0	1 (1)	0
Irritability	0	0	1 (2)	1 (2)	1 (1)	1 (1)	0	0	1 (1)	1 (1)
Memory impairment	1 (2)	0	0	0	1 (1)	0	0	0	1 (1)	0
Mental impairment	1 (2)	0	0	0	1 (1)	0	0	0	1 (1)	0
Vision blurred	1 (2)	0	0	0	1 (1)	0	0	0	1 (1)	0
Somnolence	1 (2)	0	0	0	1 (1)	0	0	0	1 (1)	0

	**4L+ FL (*n*=59)**		**3L FL (*n*=48)**		**3L+ FL (*n*=107)**		**2L FL (*n*=23)**		**2L+ FL (*n*=130)**	
Speech disorder	0	0	1 (2)	1 (2)	1 (1)	1 (1)	0	0	1 (1)	1 (1)

[a]NE was defined as investigator-identified neurological AEs related to liso-cel. 2L, second line; 2L+, second line or later; 3L, third line; 3L+, third line or later; 4L+, fourth line or later; AE, adverse event; FL, follicular lymphoma; liso-cel, lisocabtagene maraleucel; NE, neurological event.

Supplementary Table 12. TEAEs of nervous system/psychiatric disorders regardless of attribution to liso-cel (liso-cel–treated set)[a]

	**4L+ FL (*n*=59)**		**3L FL (*n*=48)**		**3L+ FL (*n*=107)**		**2L FL (*n*=23)**		**2L+ FL (*n*=130)**	
***n* (%)**	**Any grade**	**Grade ≥3**	**Any grade**	**Grade ≥3**	**Any grade**	**Grade ≥3**	**Any grade**	**Grade ≥3**	**Any grade**	**Grade ≥3**
Headache	19 (32)	0	11 (23)	0	30 (28)	0	8 (35)	0	38 (29)	0
Tremor	9 (15)	0	7 (15)	0	16 (15)	0	2 (9)	0	18 (14)	0
Aphasia	6 (10)	1 (2)	1 (2)	0	7 (7)	1 (1)	2 (9)	0	9 (7)	1 (1)
Insomnia	4 (7)	0	1 (2)	0	5 (5)	0	2 (9)	0	7 (5)	0
Dizziness	2 (3)	0	1 (2)	0	3 (3)	0	3 (13)	0	6 (5)	0
Confusional state	3 (5)	1 (2)	0	0	3 (3)	1 (1)	0	0	3 (2)	1 (1)
Dyscalculia	2 (3)	0	1 (2)	0	3 (3)	0	0	0	3 (2)	0
Dysgraphia	1 (2)	0	2 (4)	0	3 (3)	0	0	0	3 (2)	0
Syncope	1 (2)	1 (2)	1 (2)	1 (2)	2 (2)	2 (2)	1 (4)	1 (4)	3 (2)	3 (2)
Agitation	1 (2)	0	1 (2)	0	2 (2)	0	0	0	2 (2)	0
Anxiety	1 (2)	0	1 (2)	0	2 (2)	0	0	0	2 (2)	0
Balance disorder	1 (2)	0	1 (2)	0	2 (2)	0	0	0	2 (2)	0
Disorientation	1 (2)	0	1 (2)	0	2 (2)	0	0	0	2 (2)	0
Taste disorder	1 (2)	0	0	0	1 (1)	0	1 (4)	0	2 (2)	0
Adjustment disorder	0	0	1 (2)	0	1 (1)	0	0	0	1 (1)	0
Ataxia	1 (2)	0	0	0	1 (1)	0	0	0	1 (1)	0
Basal ganglia infarction	1 (2)	0	0	0	1 (1)	0	0	0	1 (1)	0
Carpal tunnel syndrome	1 (2)	0	0	0	1 (1)	0	0	0	1 (1)	0
Cognitive disorder	1 (2)	0	0	0	1 (1)	0	0	0	1 (1)	0
Depressed level of consciousness	0	0	1 (2)	1 (2)	1 (1)	1 (1)	0	0	1 (1)	1 (1)
Depressed mood	0	0	0	0	0	0	1 (4)	0	1 (1)	0
Disturbance in attention	1 (2)	0	0	0	1 (1)	0	0	0	1 (1)	0

	**4L+ FL (*n*=59)**		**3L FL (*n*=48)**		**3L+ FL (*n*=107)**		**2L FL (*n*=23)**		**2L+ FL (*n*=130)**	
Dysarthria	1 (2)	0	0	0	1 (1)	0	0	0	1 (1)	0
Encephalopathy	0	0	0	0	0	0	1 (4)	1 (4)	1 (1)	1 (1)
Fine motor skill dysfunction	0	0	1 (2)	0	1 (1)	0	0	0	1 (1)	0
Visual hallucination	1 (2)	0	0	0	1 (1)	0	0	0	1 (1)	0
Hyperesthesia	1 (2)	0	0	0	1 (1)	0	0	0	1 (1)	0
Irritability	0	0	1 (2)	1 (2)	1 (1)	1 (1)	0	0	1 (1)	1 (1)
Lethargy	1 (2)	0	0	0	1 (1)	0	0	0	1 (1)	0
Memory impairment	1 (2)	0	0	0	1 (1)	0	0	0	1 (1)	0
Mental impairment	1 (2)	0	0	0	1 (1)	0	0	0	1 (1)	0
Paresthesia	1 (2)	0	0	0	1 (1)	0	0	0	1 (1)	0
Peripheral sensory neuropathy	1 (2)	0	0	0	1 (1)	0	0	0	1 (1)	0
Sleep disorder	0	0	1 (2)	0	1 (1)	0	0	0	1 (1)	0
Somnolence	1 (2)	0	0	0	1 (1)	0	0	0	1 (1)	0
Speech disorder	0	0	1 (2)	1 (2)	1 (1)	1 (1)	0	0	1 (1)	1 (1)

[a]TEAEs of nervous system disorders or psychiatric disorders standard of care regardless of investigator assessment of relatedness to liso-cel. 2L, second line; 2L+, second line or later; 3L, third line; 3L+, third line or later; 4L+, fourth line or later; FL, follicular lymphoma; liso-cel, lisocabtagene maraleucel; TEAE, treatment-emergent adverse event.

Supplementary Table 13 | Grade 3 infections after 90-day treatment-emergent period (liso-cel–treated set)[a]

	FL cohort	Type of infection	Grade
Patient 1	3L	COVID-19	3
		COVID-19 pneumonia	4
		COVID-19 pneumonia	3
Patient 2	4L+	Progressive multifocal leukoencephalopathy	4
		Progressive multifocal leukoencephalopathy	5
Patient 3	4L+	COVID-19 pneumonia	3
		Skin infection (leg)	3

[a]One patient had 2 events of hypogammaglobulinemia concurrent with late infections (COVID-19/COVID-19 pneumonia): a grade 1 event that resolved without treatment and a grade 2 event that was treated with intravenous immunoglobulin and had not resolved at data cutoff. 3L, third line; 4L+, fourth line or later; COVID-19, coronavirus disease 2019; FL, follicular lymphoma; liso-cel, lisocabtagene maraleucel.

Supplementary Table 14 | Deaths (leukapheresed ITT set)

	2L+ FL (*n*=139)	Considered related to liso-cel per investigator	Last response per IRC before death
Total deaths before liso-cel infusion (*n*=1)			
Respiratory failure, *n*	1	No	—
Total deaths after liso-cel infusion (*n*=12)			
Disease progression, *n*	4	No	—
Grade 5 TEAE (MAS/HLH)[a], *n*	1	Yes	PD (day 29)
AE, after 90-day TE period (PML)[b], *n*	1	Yes	CR (day 90)
New malignancy, after 90-day TE period (AML)[c], *n*	2	No	Patient 1: PD (day 270)<br>Patient 2: CR (day 90)
Other, after 90-day TE period, *n*	—	—	—
Cardiac event (heart failure)[d], *n*	1	No	SD (day 29)
COVID-19, *n*	2	No	Patient 1: PD (day 180)<br>Patient 2: CR (day 90)
Erythema multiforme, *n*	1	No	CR (day 365)

[a]Sixty-six-year-old male with 2L FL (stage IV), high-risk FLIPI, met mGELF criteria, and had POD24 (i.e., had achieved PR to frontline R-CHOP and progressed on rituximab maintenance ≤6 months of initiation of R-CHOP). At baseline, the patient's bone marrow was >90% lymphoma with bone lesions and pleural effusions and they received BR as bridging therapy. After liso-cel infusion, the patient had grade 2 CRS on days 2 and 5, which was treated with tocilizumab/steroids. MAS/HLH was treated with steroids and anakinra. CMV reactivation occurred at approximately day 21 and anakinra was stopped. Rebounded MAS/HLH was not responsive to treatment with anakinra, steroids, and emapalumab. PET/CT scan on day 23 showed PR per investigator, and PD per IRC assessment, respectively, with death occurring on day 29. Before liso-cel treatment, the patient had pancytopenia and elevated ferritin based on laboratory assessment in peripheral blood. [b]Forty-four-year-old female with double-refractory disease and POD24 who received four prior lines of therapy (R-CHOP plus rituximab maintenance, BG, PI3Ki, and rituximab in combination with lenalidomide). Patient had multiple rounds of prior systemic anticancer therapy regimens containing anti-CD20 antibody, including six cycles of R-CVP (approximately 4 months) plus rituximab maintenance (approximately 2 months), five cycles of obinutuzumab plus bendamustine (approximately 4 months), and seven cycles of R2 therapy (approximately 6 months), and bridging therapy with R-CVP, before liso-cel infusion. The number and duration of anti-CD20–containing regimens before liso-cel treatment may have contributed to JCV infection susceptibility. No evidence of PML was observed on pre–liso-cel infusion MRI scans. [c]Both patients were treated in the 4L+ setting. [d]Patient experienced delay between apheresis and liso-cel administration due to persistent COVID-19 infection. 2L, second line; 2L+, second line or later; 4L+, fourth line or later; AE, adverse event; AML, acute myeloid leukemia; BG, bendamustine plus obinutuzumab; BR, bendamustine plus rituximab; CMV, cytomegalovirus; COVID-19, coronavirus disease 2019; CT, computed tomography; CR, complete response; CRS, cytokine

release syndrome; FL, follicular lymphoma; FLIPI, Follicular Lymphoma International Prognostic Index; IRC, independent review committee; ITT, intent-to-treat; JCV, John Cunningham virus; liso-cel, lisocabtagene maraleucel; MAS/HLH, macrophage activation syndrome/hemophagocytic lymphohistiocytosis; mGELF, modified Groupe d'Etude des Lymphomes Folliculaires; MRI, magnetic resonance imaging; PD, progressive disease; PET, positron emission tomography; PI3Ki, phosphoinositide 3-kinase inhibitor; PML, progressive multifocal leukoencephalopathy; POD24, disease progression ≤24 months; PR, partial response; R2, lenalidomide plus rituximab; R-CHOP, rituximab plus cyclophosphamide, doxorubicin, vincristine, and prednisone; R-CVP, rituximab plus cyclophosphamide, vincristine, and prednisone; SD, stable disease; TE, treatment emergent; TEAE, treatment-emergent adverse event.

Supplementary Table 15 | ICU length of stay and AEs

	ICU length of stay, days	AEs leading to ICU hospitalization	Grade
Patient 1	3	CRS[a]	3
Patient 2	7	Syncope[b]	3
Patient 3	6	Allergy to immunoglobulin therapy	4
		MAS/HLH	5

[a]CRS was characterized by grade 3 hypotension and grade 1 fever. [b]Syncope per atrioventricular conduction failure (conduction disorder). AE, adverse event; CRS, cytokine release syndrome; ICU, intensive care unit; MAS/HLH, macrophage activation syndrome/hemophagocytic lymphohistiocytosis.

Supplementary Table 16 | Summary of safety after liso-cel administration in patients monitored in the outpatient setting (outpatient analysis set)[a]

**AESI, *n* (%)**	**Outpatients (2L+; *n*=15)[b]**
CRS[c]	6 (40)
Grade 1	4 (27)
Grade 2	2 (13)
Grade 3	0
Grade 4	0
Grade 5	0
NEs[d]	2 (13)
Grade 1	2 (13)
Grade 2	0
Grade 3	0
Grade 4	0
Grade 5	0
Hypogammaglobulinemia[e]	1 (7)
Prolonged cytopenia[f]	0
Grade ≥3 infection	0
Second primary malignancy[e]	0
MAS/HLH	0
Infusion-related reaction	0
Tumor lysis syndrome	0
Patients hospitalized	7 (47)
Median time to initial hospitalization (range)[g], days	7 (4–16)
Admitted to the ICU	0
Median duration of initial hospitalization (range), days	5 (3–8)

[a]The Outpatient Analysis Set included all liso-cel–treated patients who were monitored as an outpatient. A patient was considered to be monitored as an outpatient if, following liso-cel infusion, the patient was monitored initially as an outpatient, regardless of liso-cel administration setting. [b]Only 1 patient in the 2L FL cohort received liso-cel in an outpatient setting. The patient was hospitalized (non-ICU stay) due to NE characterized by grade 1 symptoms of aphasia and feeling abnormal at 7 days after liso-cel administration for a duration of 3 days. NE symptoms resolved in 1–2 days. [c]Graded according to the Lee 2014 criteria. [d]Defined as investigator-identified neurological adverse events related to liso-cel and graded per the National Cancer Institute Common Terminology Criteria for Adverse Events, version 5.0. [e]Could occur within or beyond the 90-day treatment-emergent period. [f]Defined as grade ≥3 laboratory abnormalities of neutropenia, anemia, or thrombocytopenia on day 29. [g]The time to first hospitalization after liso-cel treatment among outpatients who required hospitalization for treatment or care due to postinfusion complications (e.g., CRS, NEs). 2L+, second line or later; AESI, adverse event of special interest; CRS, cytokine release syndrome; ICU, intensive care unit; liso-cel, lisocabtagene maraleucel; MAS/HLH, macrophage activation syndrome/hemophagocytic lymphohistiocytosis; NE, neurological event.

Supplementary Table 17 | Liso-cel cellular kinetic parameters (cellular kinetic set[a,b])

Median liso-cel transgene by PCR (IQR)	4L+ FL (*n*=59)	3L FL (*n*=46)	3L+ FL (*n*=105)	2L FL (*n*=23)	2L+ FL (*n*=128)
C_{max}, copies/µg	29,170 (10,039–82,196) (*n*=59)	39,116 (13,804–124,019) (*n*=46)	30,530 (12,412–96,795) (*n*=105)	62,091 (45,428–176,273) (*n*=23)	42,026 (13,537–110,390) (*n*=128)
t_{max}, days	10.0 (7.0–13.0) (*n*=59)	10.0 (9.0–10.0) (*n*=46)	10.0 (8.0–11.0) (*n*=105)	10.0 (9.0–10.0) (*n*=23)	10.0 (8.0–11.0) (*n*=128)
$AUC_{(0–28d)}$, days*copies/µg	216,973 (77,145–512,587) (*n*=56)	270,781 (112,138–908,964) (*n*=44)	253,400 (105,912–622,704) (*n*=100)	385,668 (194,260–921,947) (*n*=20)	260,274 (106,797–673,556) (*n*=120)

[a]Patients in the liso-cel–treated set with any available measurements of cellular kinetics by PCR. [b]The patient with MAS/HLH had C_{max} of 121,758 copies/µg, but AUC was not evaluable due to sample availability. C_{max} in this patient was approximately 2.0-fold higher than the median C_{max} of the 2L group (62,091 copies/µg) but within its IQR (45,428–176,273 copies/µg) and approximately 2.9-fold higher than the median C_{max} of the 2L+ group (i.e., all patients with FL) (42,026 copies/µg), but within its range (238–688,672 copies/µg). 2L, second line; 2L+, second line or later; 3L, third line; 3L+, third line or later; 4L+, fourth line or later; $AUC_{(0–28d)}$, area under the curve for liso-cel transgene levels from 0 to 28 days after infusion; C_{max}, peak liso-cel transgene level after infusion; FL, follicular lymphoma; IQR, interquartile range; LBCL, large B-cell lymphoma; liso-cel, lisocabtagene maraleucel; MAS/HLH, macrophage activation syndrome/hemophagocytic lymphohistiocytosis; PCR, polymerase chain reaction; R/R, relapsed/refractory; t_{max}, time from liso-cel infusion to peak transgene levels.

Supplementary Table 18 | Liso-cel transgene persistence[a] (cellular kinetic set[b])

**Transgene, *n*/*N* (%)**	**4L+ FL (*n*=59)**	**3L FL (*n*=48)**	**3L+ FL (*n*=107)**	**2L FL (*n*=23)**	**2L+ FL (*n*=130)**
Day 29	54/56 (96)	44/46 (96)	98/102 (96)	21/22 (95)	119/124 (96)
Day 60	46/52 (88)	37/44 (84)	83/96 (86)	12/17 (71)	95/113 (84)
Day 90	33/56 (59)	33/45 (73)	66/101 (65)	8/21 (38)	74/122 (61)
Month 6	23/49 (47)	16/39 (41)	39/88 (44)	6/20 (30)	45/108 (42)
Month 9	22/46 (48)	19/39 (49)	41/85 (48)	5/20 (25)	46/105 (44)
Month 12	19/41 (46)	14/33 (42)	33/74 (45)	5/18 (28)	38/92 (41)
Month 18	14/28 (50)	8/21 (38)	22/49 (45)	2/10 (20)	24/59 (41)
Month 24	0/0 (0)	1/3 (33)	1/3 (33)	0/1 (0)	1/4 (25)

[a]Persistence was defined as a transgene count greater than or equal to the lower limit of quantitation (16 copies/reaction, which is around 40 copies/μg). [b]Patients in the liso-cel–treated set with any available measurements of cellular kinetics by PCR. 2L, second line; 2L+, second line or later; 3L, third line; 3L+, third line or later; 4L+, fourth line or later; FL, follicular lymphoma; liso-cel, lisocabtagene maraleucel; *n*/*N*, number of patients with liso-cel transgene persistence divided by the number of patients who were evaluable for cellular kinetics at each time point; PCR, polymerase chain reaction.

Supplementary Table 19 | B-cell aplasia[a] (liso-cel–treated set[b])

***n*/*N* (%)**	**4L+ FL (*n*=59)**	**3L FL (*n*=48)**	**3L+ FL (*n*=107)**	**2L FL (*n*=23)**	**2L+ FL (*n*=130)**
Baseline[c]	47/59 (80)	34/48 (71)	81/107 (76)	18/22 (82)	99/129 (77)
Day 8	55/55 (100)	41/42 (98)	96/97 (99)	22/22 (100)	118/119 (99)
Day 15	53/54 (98)	44/44 (100)	97/98 (99)	20/20 (100)	117/118 (99)
Day 22	56/57 (98)	45/45 (100)	101/102 (99)	23/23 (100)	124/125 (99)
Day 29	57/57 (100)	45/45 (100)	102/102 (100)	21/22 (95)	123/124 (99)
Day 60	52/53 (98)	42/44 (95)	94/97 (97)	17/17 (100)	111/114 (97)
Day 90	50/55 (91)	39/42 (93)	89/97 (92)	18/21 (86)	107/118 (91)
Month 6	33/47 (70)	29/39 (74)	62/86 (72)	10/20 (50)	72/106 (68)
Month 9	29/47 (62)	29/41 (71)	58/88 (66)	8/20 (40)	66/108 (61)
Month 12	28/44 (64)	26/38 (68)	54/82 (66)	7/20 (35)	61/102 (60)
Month 18	18/31 (58)	16/23 (70)	34/54 (63)	3/11 (27)	37/65 (57)

[a]Defined as <3% $CD19^+$ B cells in peripheral blood lymphocytes as assessed by flow cytometry. [b]Patients in the liso-cel–treated set with available B-cell aplasia data. [c]Defined as the last measurement before liso-cel infusion. 2L, second line; 2L+, second line or later; 3L, third line; 3L+, third line or later; 4L+, fourth line or later; FL, follicular lymphoma; liso-cel, lisocabtagene maraleucel; *n*/*N*, number of patients with B-cell aplasia divided by the number of patients evaluable for B-cell aplasia at each time point.

Supplementary Table 20 | Overall least square mean changes from baseline (PRO analysis set)[a]

Domain[b]	Overall least squares mean change (95% CI)[c]		MID[d]	
	3L+ FL	2L FL	Improvement	Deterioration
**EORTC QLQ-C30**	*n*=97	*n*=19		
Global health status/QOL	*3.2 (0.9, 5.4)[e]*	*1.3 (−2.7, 5.2)*	*5*	*−5*
Physical functioning	*0.7 (−0.9, 2.4)*	*0.7 (−2.5, 3.9)*	*2*	*−5*
Role functioning	*0.2 (−2.6, 2.9)*	*3.4 (−2.2, 9.1)*	*6*	*−7*
Cognitive functioning	*1.9 (−0.1, 4.0)*	*2.1 (−2.7, 6.8)*	*3*	*−1*
Fatigue	*−1.7 (−4.5, 1.0)*	***−6.7 (−10.6, −2.8)[e]***	*−4*	*5*
Pain	*−4.5 (−7.1, −2.0)[e]*	***−6.4 (−10.9, −2.0)[e]***	*−5*	*3*
Emotional functioning	5.6 (3.2, 8.1)[e]	**9.5 (4.7, 14.3)[e]**	6	−3
Social functioning	1.2 (−2.0, 4.4)	−2.5 (−8.8, 3.8)	3	−6
Nausea and vomiting	1.8 (−0.03, 3.5)	1.2 (−1.2, 3.7)	−3	5
Dyspnea	**−6.4 (−8.7, −4.0)[e]**	**−9.3 (−13.7, −4.9)[e]**	−2	5
Insomnia	**−7.3 (−10.5, −4.1)[e]**	**−7.9 (−15.8, 0.1)**	−5	2
Appetite loss	−0.8 (−3.0, 1.5)	−0.9 (−4.8, 2.9)	−7	2
Constipation	−2.8 (−5.4, −0.3)[e]	1.8 (−2.8, 6.3)	−4	5
Diarrhea	**−5.8 (−7.8, −3.9)[e]**	**−4.7 (−9.7, 0.4)**	−3	5
Financial difficulties	−0.5 (−4.6, 3.6)	−1.6 (−8.0, 4.7)	−3	2
**FACT-LymS**	*n*=102	*n*=20		
	1.9 (1.1, 2.8)[e]	***3.3 (1.8, 4.8)[e]***	*3*	*−3*

[a]Baseline was defined as the latest measurement within 7 days before LDC. [b]Primary domains of interest are in italics; all others are secondary domains of interest. The analysis was based on changes in PROs from baseline through day 730. A positive score denotes improvement in PROs or functional status for EORTC QLQ-C30, and FACT-LymS; a negative score denotes improvement in symptoms for the symptom domains of the EORTC QLQ-C30. [c]Bold numbers denote clinically meaningful improvements. [d]MID thresholds are defined for EORTC QLQ-C30[1] and FACT-LymS[2]. [e]*P* < 0.05. 2L, second line; 3L+, third line or later; CI, confidence interval; EORTC QLQ-C30, European Organisation for Research and Treatment of Cancer Quality of life Questionnaire-Core 30 items; FACT-LymS, Functional Assessment of Cancer Therapy-Lymphoma “Additional Concerns” Scale; FL, follicular lymphoma; LDC, lymphodepleting chemotherapy; MID, minimally important difference; NE, not evaluable; PRO, patient-reported outcome; QOL, quality of life.

Supplementary Table 21 | Study population definitions

Analysis set	Definition
Screened set	• All patients who signed informed consent
Eligible set	• All patients who signed informed consent, and who met all inclusion/exclusion criteria
Leukapheresed (ITT) analysis set	• All patients who signed informed consent, who met all inclusion/exclusion criteria, and who underwent leukapheresis<br>• In case of protocol deviations where patients underwent leukapheresis without meeting all inclusion/exclusion criteria, the patients will still be included in the leukapheresed set
Liso-cel–treated set	• All patients who received a dose of conforming liso-cel product
Efficacy set	• All patients in the liso-cel–treated set who had positive disease present before liso-cel administration based on IRC assessment (PET/CT)<br>• Patients who did not have baseline assessment repeated after anticancer therapy for disease control and before liso-cel administration were excluded from the liso-cel–treated efficacy analysis set
Outpatient set	• All patients in the liso-cel–treated set who were monitored as an outpatient<br>• A patient was considered to be monitored as an outpatient if, after liso-cel administration, the patient was monitored initially as an outpatient, regardless of liso-cel administration setting
Cellular kinetic set	• Patients in the liso-cel–treated set who had any available cellular kinetic measurements by PCR
PRO analysis set	• Included patients in the liso-cel–treated set who completed their baseline PRO questionnaires and have ≥1 postbaseline PRO measurement

CT, computed tomography; IRC, independent review committee; ITT, intent-to-treat; liso-cel, lisocabtagene maraleucel; PCR, polymerase chain reaction; PET, positron emission tomography; PRO, patient-reported outcome.

Supplementary Fig. 1 | Forest plot of ORR per IRC assessment in 3L+ (efficacy set).

	Evaluable patients, *n*	Patients with ORR, *n*	ORR, % (95% CI)
Overall	101	98	97.0 (91.6–99.4)
Age, years			
<65 years	60	58	96.7 (88.5–99.6)
≥65 and <70 years	32	31	96.9 (83.8–99.9)
≥75 years	9	9	100.0 (66.4–100.0)
Sex (biological attribute)			
Male	62	61	98.4 (91.3–100.0)
Female	39	37	94.9 (82.7–99.4)
Race			
White	57	56	98.2 (90.6–100.0)
Non-White	12	12	100.0 (73.5–100.0)
Asian	9	9	100.0 (66.4–100.0)
Non-Asian	60	59	98.3 (91.1–100.0)
Ethnicity			
Hispanic or Latino	5	4	80.0 (28.4–99.5)
Not Hispanic or Latino	71	70	98.6 (92.4–100.0)
Region			
North America	35	34	97.1 (85.1–99.9)
Europe	58	56	96.6 (88.1–99.6)
Japan	8	8	100.0 (63.1–100.0)
FLIPI at screening			
Low (0–1)	10	10	100.0 (69.2–100.0)
Intermediate (2)	33	33	100.0 (89.4–100.0)
High (3–5)	58	55	94.8 (85.6–98.9)
mGELF criteria			
Yes	54	51	94.4 (84.6–98.8)
No	47	47	100.0 (92.5–100.0)
Bulky disease at screening[b]			
Yes	31	30	96.8 (83.3–99.9)
No	70	68	97.1 (90.1–99.7)
LDH at pre-LDC			
≤ULN	56	55	98.2 (90.4–100.0)
>ULN	45	43	95.6 (84.9–99.5)
No. of prior systemic lines of therapies			
1	0	0	NA
2	48	47	97.9 (88.9–99.9)
3	24	23	95.8 (78.9–99.9)
≥4	29	28	96.6 (82.2–99.9)
Prior ASCT			
Yes	30	28	93.3 (77.9–99.2)
No	71	70	98.6 (92.4–100.0)
Prior PI3Ki			
Yes	24	22	91.7 (73.0–99.0)
No	77	76	98.7 (93.0–100.0)
Prior R2			
Yes	20	19	95.0 (75.1–99.9)
No	81	79	97.5 (91.4–99.7)
Prior bendamustine			
None	42	41	97.6 (87.4–99.9)
≤6 months before leukapheresis	2	2	NA
>6 and ≤12 months before leukapheresis	3	3	NA
>12 months before leukapheresis	54	52	96.3 (87.3–99.5)
Bridging therapy			
Yes	40	38	95.0 (83.1–99.4)
No	61	60	98.4 (91.2–100.0)
Relapsed vs. refractory			
Relapsed	67	66	98.5 (92.0–100.0)
Refractory	34	32	94.1 (80.3–99.3)
PD on last line of therapy or ≤6 months after last line			
Yes	61	58	95.1 (86.3–99.0)
No	40	40	100.0 (91.2–100.0)
PD or SD to last line of therapy or ≤6 months after last line			
Yes	64	61	95.3 (86.9–99.0)
No	37	37	100.0 (90.5–100.0)
Double refractory (anti-CD20 + alkylator)			
Yes	66	63	95.5 (87.3–99.1)
No	35	35	100.0 (90.0–100.0)
Double refractory[c]			
Yes	40	38	95.0 (83.1–99.4)
No	61	60	98.4 (91.2–100.0)
Double refractory[d]			
Yes	71	68	95.8 (88.1–99.1)
No	30	30	100.0 (88.4–100.0)
POD24 from initial immunochemotherapy[e]			
Yes	56	54	96.4 (87.7–99.6)
No	44	43	97.7 (88.0–99.9)
POD24 from diagnosis[f]			
Yes	44	43	97.7 (88.0–99.9)
No	57	55	96.5 (87.9–99.6)
GCSF			
Yes	50	48	96.0 (86.3–99.5)
No	51	50	98.0 (89.6–100.0)

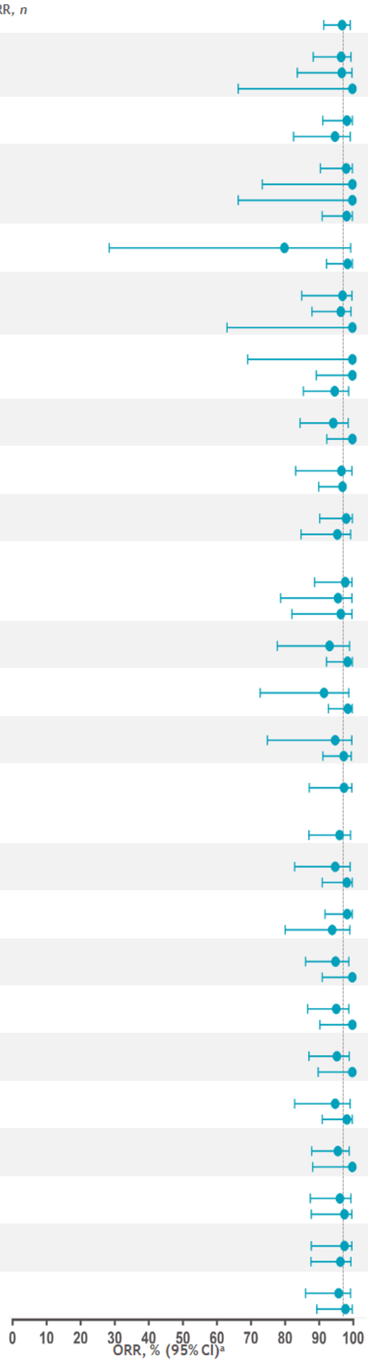

● Point estimates of ORR ⊢⊣ 95% CI based on the efficacy-evaluable population

[a]Two-sided 95% CI was based on exact Clopper-Pearson method. ORR difference was not computed for subset with <5 patients per analysis group. [b]Bulky disease was defined as any mass >7 cm, or 3 or more masses (each >3 cm) at screening based on investigator assessment. [c]Patients who did not respond or progressed during or up to 6 months after completing combination treatment with an anti-CD20 monoclonal antibody and alkylating agent. [d]Patients who did not respond or progressed during or up to 6 months after completing maintenance treatment with an anti-CD20 monoclonal antibody. Anti-CD20 maintenance defined as patients who completed an immediate course of combination treatment with anti-CD20 and alkylating agent. Assessment of SD or PD from start of anti-CD20 maintenance therapy up to and including 6 months after completion of anti-CD20 maintenance therapy. [e]FL progression within 24 months of first-line therapy with anti-CD20 antibody and alkylator. [f]Progression of disease within 24 months of diagnosis after treatment with an anti-CD20 monoclonal antibody and alkylating agent within 6 months of initial FL diagnosis. 3L+, third line or later; ASCT, autologous stem cell transplantation; CI, confidence interval; FL, follicular lymphoma; FLIPI, Follicular Lymphoma International Prognostic Index; GCSF, granulocyte colony-stimulating factor; IRC, independent review committee; LDC, lymphodepleting chemotherapy; LDH, lactate dehydrogenase; mGELF, modified Groupe d'Etude des Lymphomes Folliculaires; NA, not available; ORR, overall response rate; PD, progressive disease; PI3Ki, phosphoinositide 3-kinase inhibitor; POD24, progression of disease ≤24 months; R2, lenalidomide plus rituximab; SD, stable disease; ULN, upper limit of normal.

Supplementary Fig. 2 | Forest plot of ORR per IRC assessment in 2L+ (efficacy set).

	Evaluable patients, *n*	Patients with ORR, *n*	ORR, % (95% CI)
Overall	124	120	96.8 (91.9–99.1)
**Age, years**			
<65 years	78	76	97.4 (91.0–99.7)
≥65 and <70 years	37	35	94.6 (81.8–99.3)
≥75 years	9	9	100.0 (66.4–100.0)
**Sex (biological attribute)**			
Male	79	77	97.5 (91.2–99.7)
Female	45	43	95.6 (84.9–99.5)
**Race**			
White	66	65	98.5 (91.8–100.0)
Non-White	15	15	100.0 (78.2–100.0)
Asian	11	11	100.0 (71.5–100.0)
Non-Asian	70	69	98.6 (92.3–100.0)
**Ethnicity**			
Hispanic or Latino	6	5	83.3 (35.9–99.6)
Not Hispanic or Latino	86	84	97.7 (91.9–99.7)
**Region**			
North America	45	43	95.6 (84.9–99.5)
Europe	70	68	97.1 (90.1–99.7)
Japan	9	9	100.0 (66.4–100.0)
**FLIPI at screening**			
Low (0–1)	21	21	100.0 (83.9–100.0)
Intermediate (2)	37	37	100.0 (90.5–100.0)
High (3–5)	66	62	93.9 (85.2–98.3)
**mGELF criteria**			
Yes	70	66	94.3 (86.0–98.4)
No	54	54	100.0 (93.4–100.0)
**Bulky disease at screening[b]**			
Yes	41	40	97.6 (87.1–99.9)
No	83	80	96.4 (89.8–99.2)
**LDH at pre-LDC**			
≤ULN	73	71	97.3 (90.5–99.7)
>ULN	51	49	96.1 (86.5–99.5)
**No. of prior systemic lines of therapies**			
1	23	22	95.7 (78.1–99.9)
2	48	47	97.9 (88.9–99.9)
3	24	23	95.8 (78.9–99.9)
≥4	29	28	96.6 (82.2–99.9)
**Prior ASCT**			
Yes	30	28	93.3 (77.9–99.2)
No	94	92	97.9 (92.5–99.7)
**Prior PI3Ki**			
Yes	24	22	91.7 (73.0–99.0)
No	100	98	98.0 (93.0–99.8)
**Prior R2**			
Yes	20	19	95.0 (75.1–99.9)
No	104	101	97.1 (91.8–99.4)
**Prior bendamustine**			
None	59	57	96.6 (88.3–99.6)
≤6 months before leukapheresis	3	3	NA
>6 and ≤12 months before leukapheresis	5	5	100.0 (47.8–100.0)
>12 months before leukapheresis	57	55	96.5 (87.9–99.6)
**Bridging therapy**			
Yes	45	42	93.3 (81.7–98.6)
No	79	78	98.7 (93.1–100.0)
**Relapsed vs. refractory**			
Relapsed	87	85	97.7 (91.9–99.7)
Refractory	37	35	94.6 (81.8–99.3)
**PD on last line of therapy or ≤6 months after last line**			
Yes	76	72	94.7 (87.1–98.5)
No	48	48	100.0 (92.6–100.0)
**PD or SD to last line of therapy or ≤6 months after last line**			
Yes	79	75	94.9 (87.5–98.6)
No	45	45	100.0 (92.1–100.0)
**Double refractory (anti-CD20 + alkylator)**			
Yes	77	73	94.8 (87.2–98.6)
No	47	47	100.0 (92.5–100.0)
**Double refractory[c]**			
Yes	49	46	93.9 (83.1–98.7)
No	75	74	98.7 (92.8–100.0)
**Double refractory[d]**			
Yes	86	82	95.3 (88.5–98.7)
No	38	38	100.0 (90.7–100.0)
**POD24 from initial immunochemotherapy[e]**			
Yes	71	68	95.8 (88.1–99.1)
No	52	51	98.1 (89.7–100.0)
**POD24 from diagnosis[f]**			
Yes	56	54	96.4 (87.7–99.6)
No	68	66	97.1 (89.8–99.6)
**GCSF**			
Yes	60	57	95.0 (86.1–99.0)
No	64	63	98.4 (91.6–100.0)

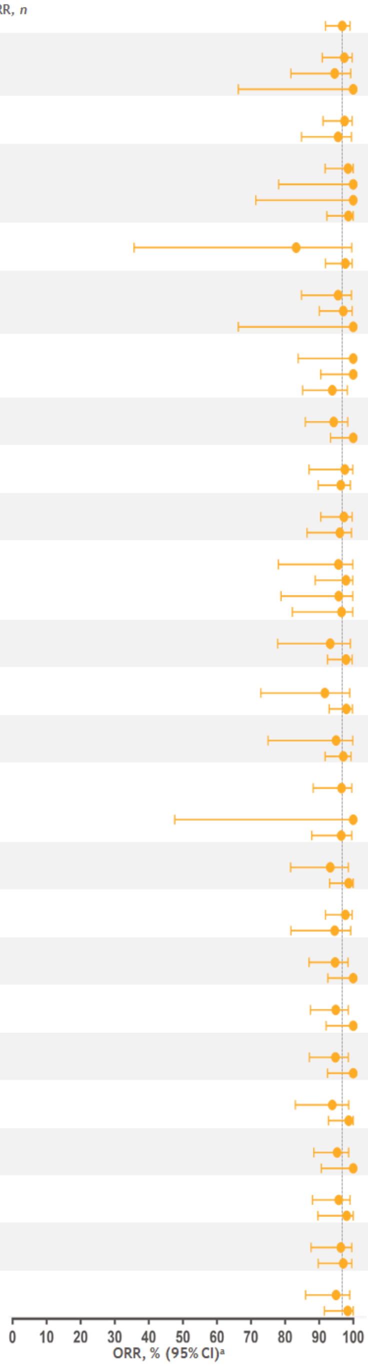

● Point estimates of ORR   ⊢⊣ 95% CI based on the efficacy-evaluable population

[a]Two-sided 95% CI was based on exact Clopper-Pearson method. ORR difference was not computed for subset with <5 patients per analysis group. [b]Bulky disease was defined as any mass >7 cm, or 3 or more masses (each >3 cm) at screening based on investigator assessment. [c]Patients who did not respond or progressed during or up to 6 months after completing combination treatment with an anti-CD20 monoclonal antibody and alkylating agent. [d]Patients who did not respond or progressed during or up to 6 months after completing maintenance treatment with an anti-CD20 monoclonal antibody. Anti-CD20 maintenance defined as patients who completed an immediate course of combination treatment with anti-CD20 and alkylating agent. Assessment of SD or PD from start of anti-CD20 maintenance therapy up to and including 6 months after completion of anti-CD20 maintenance therapy. [e]FL progression within 24 months of first-line therapy with anti-CD20 antibody and alkylating agent. [f]Progression of disease within 24 months of diagnosis after treatment with an anti-CD20 monoclonal antibody and alkylating agent within 6 months of initial FL diagnosis. 2L+, second line or later; ASCT, autologous stem cell transplantation; CI, confidence interval; FL, follicular lymphoma; FLIPI, Follicular Lymphoma International Prognostic Index; GCSF, granulocyte colony-stimulating factor; IRC, independent review committee; LDC, lymphodepleting chemotherapy; LDH, lactate dehydrogenase; mGELF, modified Groupe d'Etude des Lymphomes Folliculaires; NA, not available; ORR, overall response rate; PD, progressive disease; PI3Ki, phosphoinositide 3-kinase inhibitor; POD24, progression of disease ≤24 months; R2, lenalidomide plus rituximab; SD, stable disease; ULN, upper limit of normal.

Supplementary Fig. 3 | Forest plot of CRR per IRC assessment in 3L+ (efficacy set).

	Evaluable patients, *n*	Patients with CRR, *n*	CRR, % (95% CI)
Overall	101	95	94.1 (87.5–97.8)
**Age, years**			
<65 years	60	57	95.0 (86.1–99.0)
≥65 and <70 years	32	30	93.8 (79.2–99.2)
≥75 years	9	8	88.9 (51.8–99.7)
**Sex (biological attribute)**			
Male	62	59	95.2 (86.5–99.0)
Female	39	36	92.3 (79.1–98.4)
**Race**			
White	57	55	96.5 (87.9–99.6)
Non-White	12	11	91.7 (61.5–99.8)
Asian	9	9	100.0 (66.4–100.0)
Non-Asian	60	57	95.0 (86.1–99.0)
**Ethnicity**			
Hispanic or Latino	5	4	80.0 (28.4–99.5)
Not Hispanic or Latino	71	67	94.4 (86.2–98.4)
**Region**			
North America	35	32	91.4 (76.9–98.2)
Europe	58	55	94.8 (85.6–98.9)
Japan	8	8	100.0 (63.1–100.0)
**FLIPI at screening**			
Low (0–1)	10	10	100.0 (69.2–100.0)
Intermediate (2)	33	32	97.0 (84.2–99.9)
High (3–5)	58	53	91.4 (81.0–97.1)
**mGELF criteria**			
Yes	54	50	92.6 (82.1–97.9)
No	47	45	95.7 (85.5–99.5)
**Bulky disease at screening[b]**			
Yes	31	29	93.5 (78.6–99.2)
No	70	66	94.3 (86.0–98.4)
**LDH at pre-LDC**			
≤ULN	56	54	96.4 (87.7–99.6)
>ULN	45	41	91.1 (78.8–97.5)
**No. of prior systemic lines of therapies**			
1	0	0	NA
2	48	46	95.8 (85.7–99.5)
3	24	22	91.7 (73.0–99.0)
≥4	29	27	93.1 (77.2–99.2)
**Prior ASCT**			
Yes	30	28	93.3 (77.9–99.2)
No	71	67	94.4 (86.2–98.4)
**Prior PI3Ki**			
Yes	24	21	87.5 (67.6–97.3)
No	77	74	96.1 (89.0–99.2)
**Prior R2**			
Yes	20	18	90.0 (68.3–98.8)
No	81	77	95.1 (87.8–98.6)
**Prior bendamustine**			
None	42	40	95.2 (83.8–99.4)
≤6 months before leukapheresis	2	2	NA
>6 and ≤12 months before leukapheresis	3	1	NA
>12 months before leukapheresis	54	52	96.3 (87.3–99.5)
**Bridging therapy**			
Yes	40	38	95.0 (83.1–99.4)
No	61	57	93.4 (84.1–98.2)
**Relapsed vs. refractory**			
Relapsed	67	64	95.5 (87.5–99.1)
Refractory	34	31	91.2 (76.3–98.1)
**PD on last line of therapy or ≤6 months after last line**			
Yes	61	56	91.8 (81.9–97.3)
No	40	39	97.5 (86.8–99.9)
**PD or SD to last line of therapy or ≤6 months after last line**			
Yes	64	58	90.6 (80.7–96.5)
No	37	37	100.0 (90.5–100.0)
**Double refractory (anti-CD20 + alkylator)**			
Yes	66	62	93.9 (85.2–98.3)
No	35	33	94.3 (80.8–99.3)
**Double refractory[c]**			
Yes	40	37	92.5 (79.6–98.4)
No	61	58	95.1 (86.3–99.0)
**Double refractory[d]**			
Yes	71	66	93.0 (84.3–97.7)
No	30	29	96.7 (82.8–99.9)
**POD24 from initial immunochemotherapy[e]**			
Yes	56	53	94.6 (85.1–98.9)
No	44	41	93.2 (81.3–98.6)
**POD24 from diagnosis[f]**			
Yes	44	42	95.5 (84.5–99.4)
No	57	53	93.0 (83.0–98.1)
**GCSF**			
Yes	50	47	94.0 (83.5–98.7)
No	51	48	94.1 (83.8–98.8)

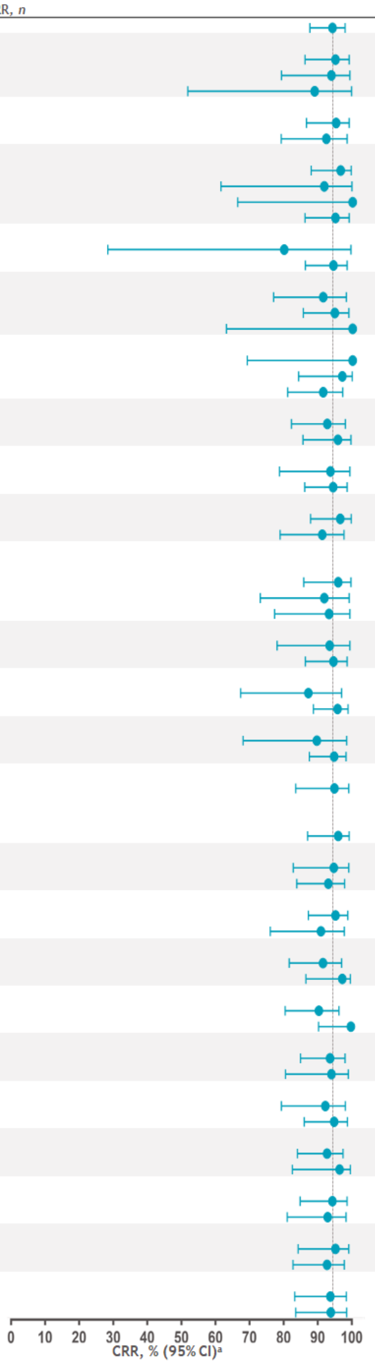

● Point estimates of CRR ⊢⊣ 95% CI based on the efficacy-evaluable population

[a]Two-sided 95% CI was based on exact Clopper-Pearson method. CRR difference was not computed for subset with <5 patients per analysis group. [b]Bulky disease was defined as any mass >7 cm, or 3 or more masses (each >3 cm) at screening based on investigator assessment. [c]Patients who did not respond or progressed during or up to 6 months after completing combination treatment with an anti-CD20 monoclonal antibody and alkylating agent. [d]Patients who did not respond or progressed during or up to 6 months after completing maintenance treatment with an anti-CD20 monoclonal antibody. Anti-CD20 maintenance defined as patients who completed an immediate course of combination treatment with anti-CD20 and alkylating agent. Assessment of SD or PD from start of anti-CD20 maintenance therapy up to and including 6 months after completion of anti-CD20 maintenance therapy. [e]FL progression within 24 months of first-line therapy with anti-CD20 antibody and alkylator. [f]Progression of disease within 24 months of diagnosis after treatment with an anti-CD20 monoclonal antibody and alkylating agent within 6 months of initial FL diagnosis. 3L+, third line or later; ASCT, autologous stem cell transplantation; CI, confidence interval; CRR, complete response rate; FL, follicular lymphoma; FLIPI, Follicular Lymphoma International Prognostic Index; GCSF, granulocyte colony-stimulating factor; IRC, independent review committee; LDC, lymphodepleting chemotherapy; LDH, lactate dehydrogenase; mGELF, modified Groupe d'Etude des Lymphomes Folliculaires; NA, not available; PD, progressive disease; PI3Ki, phosphoinositide 3-kinase inhibitor; POD24, progression of disease ≤24 months; R2, lenalidomide plus rituximab; SD, stable disease; ULN, upper limit of normal.

Supplementary Fig. 4 | Forest plot of CRR per IRC assessment in 2L+ (efficacy set).

	Evaluable patients, *n*	Patients with CRR, *n*	CRR, % (95% CI)
Overall	124	117	94.4 (88.7–97.7)
**Age, years**			
<65 years	78	75	96.2 (89.2–99.2)
≥65 and <70 years	37	34	91.9 (78.1–98.3)
≥75 years	9	8	88.9 (51.8–99.7)
**Sex (biological attribute)**			
Male	79	75	94.9 (87.5–98.6)
Female	45	42	93.3 (81.7–98.6)
**Race**			
White	66	64	97.0 (89.5–99.6)
Non-White	15	14	93.3 (68.1–99.8)
Asian	11	11	100.0 (71.5–100.0)
Non-Asian	70	67	95.7 (88.0–99.1)
**Ethnicity**			
Hispanic or Latino	6	5	83.3 (35.9–99.6)
Not Hispanic or Latino	81	86	94.2 (87.0–98.1)
**Region**			
North America	45	41	91.1 (78.8–97.5)
Europe	70	67	95.7 (88.0–99.1)
Japan	9	9	100.0 (66.4–100.0)
**FLIPI at screening**			
Low (0–1)	21	21	100.0 (83.9–100.0)
Intermediate (2)	37	36	97.3 (85.8–99.9)
High (3–5)	66	60	90.9 (81.3–96.6)
**mGELF criteria**			
Yes	70	65	92.9 (84.1–97.6)
No	54	52	96.3 (87.3–99.5)
**Bulky disease at screening[b]**			
Yes	41	39	95.1 (83.5–99.4)
No	83	78	94.0 (86.5–98.0)
**LDH at pre-LDC**			
≤ULN	73	70	95.9 (88.5–99.1)
>ULN	51	47	92.2 (81.1–97.8)
**No. of prior systemic lines of therapies**			
1	23	22	95.7 (78.1–99.9)
2	48	46	95.8 (85.7–99.5)
3	24	22	91.7 (73.0–99.0)
≥4	29	27	93.1 (77.2–99.2)
**Prior ASCT**			
Yes	30	28	93.3 (77.9–99.2)
No	94	89	94.7 (88.0–98.3)
**Prior PI3Ki**			
Yes	24	21	87.5 (67.6–97.3)
No	100	96	96.0 (90.1–98.9)
**Prior R2**			
Yes	20	18	90.0 (68.3–98.8)
No	104	99	95.2 (89.1–98.4)
**Prior bendamustine**			
None	59	56	94.9 (85.9–98.9)
≤6 months before leukapheresis	3	3	NA
>6 and ≤12 months before leukapheresis	5	3	60.0 (14.7–94.7)
>12 months before leukapheresis	57	55	96.5 (87.9–99.6)
**Bridging therapy**			
Yes	45	42	93.3 (81.7–98.6)
No	79	75	94.9 (87.5–98.6)
**Relapsed vs. refractory**			
Relapsed	87	83	95.4 (88.6–98.7)
Refractory	37	34	91.9 (78.1–98.3)
**PD on last line of therapy or ≤6 months after last line**			
Yes	76	70	92.1 (83.6–97.0)
No	48	47	97.9 (88.9–99.9)
**PD or SD to last line of therapy or ≤6 months after last line**			
Yes	79	72	91.1 (82.6–96.4)
No	45	45	100.0 (92.1–100.0)
**Double refractory (anti-CD20 + alkylator)**			
Yes	77	72	93.5 (85.5–97.9)
No	47	45	95.7 (85.5–99.5)
**Double refractory[c]**			
Yes	49	45	91.8 (80.4–97.7)
No	75	72	96.0 (88.8–99.2)
**Double refractory[d]**			
Yes	86	80	93.0 (85.4–97.4)
No	38	37	97.4 (86.2–99.9)
**POD24 from initial immunochemotherapy[e]**			
Yes	71	67	94.4 (86.2–98.4)
No	52	49	94.2 (84.1–98.8)
**POD24 from diagnosis[f]**			
Yes	56	53	94.6 (85.1–98.9)
No	68	64	94.1 (85.6–98.4)
**GCSF**			
Yes	60	56	93.3 (83.8–98.2)
No	64	61	95.3 (86.9–99.0)

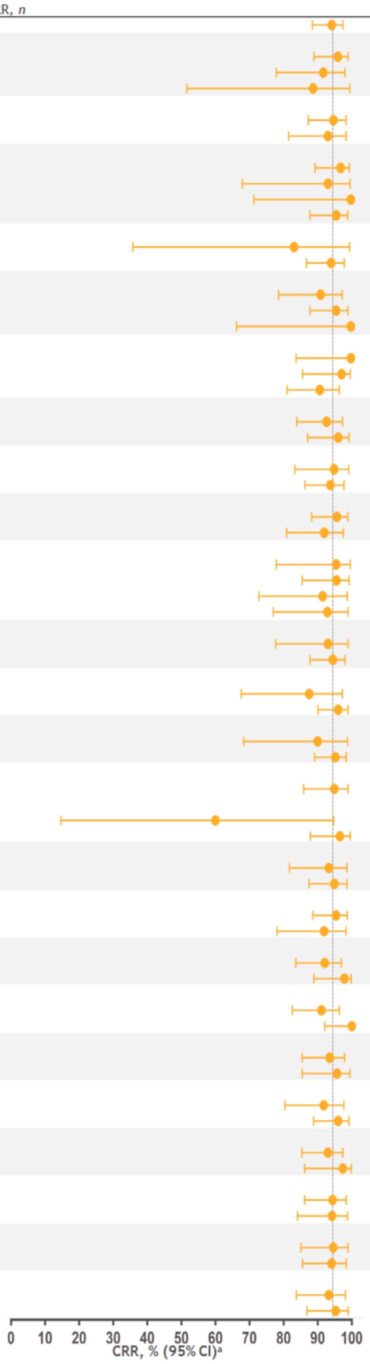

● Point estimates of CRR ├─┤ 95% CI based on the efficacy-evaluable population

[a]Two-sided 95% CI was based on exact Clopper-Pearson method. CRR difference was not computed for subset with <5 patients per analysis group. [b]Bulky disease was defined as any mass >7 cm, or 3 or more masses (each >3 cm) at screening based on investigator assessment. [c]Patients who did not respond or progressed during or up to 6 months after completing combination treatment with an anti-CD20 monoclonal antibody and alkylating agent. [d]Patients who did not respond or progressed during or up to 6 months after completing maintenance treatment with an anti-CD20 monoclonal antibody. Anti-CD20 maintenance defined as patients who completed an immediate course of combination treatment with anti-CD20 and alkylating agent. Assessment of SD or PD from start of anti-CD20 maintenance therapy up to and including 6 months after completion of anti-CD20 maintenance therapy. [e]FL progression within 24 months of first-line therapy with anti-CD20 antibody and alkylator. [f]Progression of disease within 24 months of diagnosis after treatment with an anti-CD20 monoclonal antibody and alkylating agent within 6 months of initial FL diagnosis. 2L+, second line or later; ASCT, autologous stem cell transplantation; CI, confidence interval; CRR, complete response rate; FL, follicular lymphoma; FLIPI, Follicular Lymphoma International Prognostic Index; GCSF, granulocyte colony-stimulating factor; IRC, independent review committee; LDC, lymphodepleting chemotherapy; LDH, lactate dehydrogenase; mGELF, modified Groupe d'Etude des Lymphomes Folliculaires; NA, not available; PD, progressive disease; PI3Ki, phosphoinositide 3-kinase inhibitor; POD24, progression of disease ≤24 months; R2, lenalidomide plus rituximab; SD, stable disease; ULN, upper limit of normal.

Supplementary Fig. 5 | Forest plot of 12-month continued response rate (DOR) per IRC assessment in 3L+ FL (efficacy set).

	Evaluable Patients (no.)	Patients With Response (no.)	12-Month DOR, % (95% CI)
Overall	101	98	81.9 (72.5–88.4)
Age — yr			
<65 yrs	60	58	87.0 (74.5–93.6)
≥65 and <70 yrs	32	31	77.4 (58.4–88.5)
≥75 yrs	9	9	66.7 (28.2–87.8)
Sex (biological attribute)			
Male	62	61	79.7 (66.9–87.9)
Female	39	37	85.9 (69.4–93.9)
Race			
White	57	56	85.2 (72.5–92.3)
Non-White	12	12	83.3 (48.2–95.6)
Asian	9	9	88.9 (43.3–98.4)
Non-Asian	60	59	84.2 (71.9–91.5)
Ethnicity			
Hispanic or Latino	5	4	75.0 (12.8–96.1)
Not Hispanic or Latino	71	70	82.0 (70.3–89.4)
Region			
North America	35	34	82.4 (64.9–91.7)
Europe	58	56	80.6 (66.7–89.1)
Japan	8	8	87.5 (38.7–98.1)
FLIPI at screening			
Low (0–1)	10	10	100.0 (100.0–100.0)
Intermediate (2)	33	33	89.7 (71.0–96.6)
High (3–5)	58	55	74.3 (60.4–83.9)
mGELF criteria			
Yes	54	51	75.3 (60.5–85.2)
No	47	47	88.9 (75.3–95.2)
Bulky disease at screening[b]			
Yes	31	30	78.6 (58.3–89.8)
No	70	68	83.3 (71.8–90.4)
LDH at pre-LDC			
≤ULN	56	55	88.6 (76.2–94.7)
>ULN	45	43	73.4 (57.1–84.3)
No. of prior systemic lines of therapies			
1	0	0	N/A
2	48	47	81.8 (66.8–90.5)
3	24	23	91.3 (69.5–97.8)
≥4	29	28	74.5 (53.7–87.0)
Prior ASCT			
Yes	30	28	96.4 (77.2–99.5)
No	71	70	76.1 (63.9–84.7)
Prior PI3Ki			
Yes	24	22	67.6 (43.7–83.1)
No	77	76	86.2 (75.8–92.3)
Prior R2			
Yes	20	19	77.8 (51.1–91.0)
No	81	79	83.1 (72.6–89.8)
Prior bendamustine			
None	42	41	81.7 (65.3–90.9)
≤6 months before leukapheresis	2	2	N/A
>6 and ≤12 months before leukapheresis	3	3	N/A
>12 months before leukapheresis	54	52	88.1 (75.4–94.5)
Bridging therapy			
Yes	40	38	86.4 (70.2–94.1)
No	61	60	79.1 (66.1–87.6)
Relapsed vs. refractory			
Relapsed	67	66	84.5 (73.0–91.3)
Refractory	34	32	77.0 (57.6–88.3)
PD on last line of therapy or ≤6 months after last line			
Yes	61	58	78.3 (64.9–87.1)
No	40	40	87.5 (72.5–94.6)
PD or SD to last line of therapy or ≤6 months after last line			
Yes	64	61	77.7 (64.6–86.4)
No	37	37	89.2 (73.7–95.8)
Double refractory (anti-CD20 + alkylator)			
Yes	66	63	76.6 (63.6–85.5)
No	35	35	91.4 (75.7–97.2)
Double refractory[c]			
Yes	40	38	73.4 (56.2–84.7)
No	61	60	87.4 (75.3–93.8)
Double refractory[d]			
Yes	71	68	78.8 (66.7–86.9)
No	30	30	89.4 (70.5–96.5)
POD24 from initial immunochemotherapy[e]			
Yes	56	54	76.3 (61.9–85.9)
No	44	43	90.7 (77.1–96.4)
POD24 from diagnosis[f]			
Yes	44	43	73.3 (56.8–84.4)
No	57	55	88.7 (76.6–94.8)
GCSF			
Yes	50	48	80.6 (66.0–89.4)
No	51	50	83.0 (68.7–91.2)

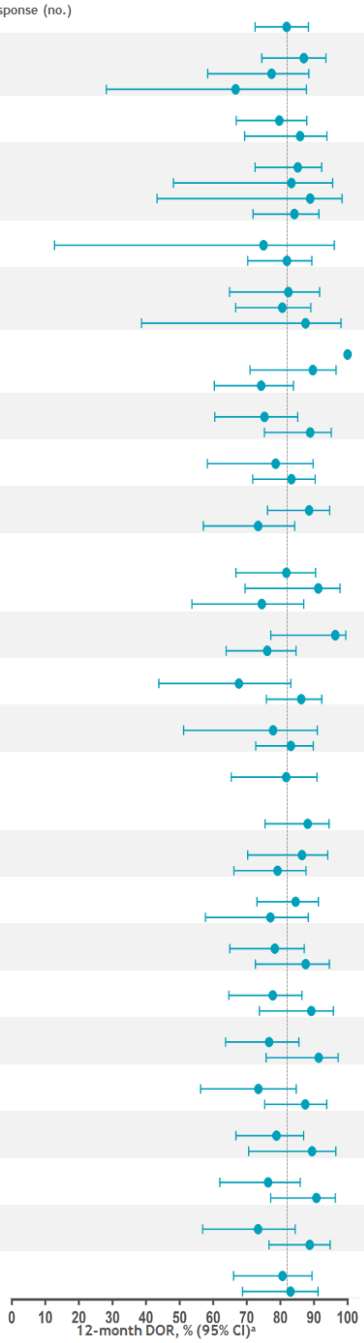

Point estimates of DOR   95% CI based on the efficacy-evaluable population

[a]Based on Kaplan–Meier estimates. DOR was defined as the interval from the first documentation of response to PD or death from any cause, whichever occurred first. [b]Bulky disease was defined as any mass >7 cm, or 3 or more masses (each >3 cm) at screening based on investigator assessment. [c]Patients who did not respond or progressed during or up to 6 months after completing combination treatment with an anti-CD20 monoclonal antibody and alkylating agent. [d]Patients who did not respond or progressed during or up to 6 months after completing maintenance treatment with an anti-CD20 monoclonal antibody. Anti-CD20 maintenance defined as patients who completed an immediate course of combination treatment with anti-CD20 and alkylating agent. Assessment of SD or PD from start of anti-CD20 maintenance therapy up to and including 6 months after completion of anti-CD20 maintenance therapy. [e]FL progression within 24 months of first-line therapy with anti-CD20 antibody and alkylator. [f]Progression of disease within 24 months of diagnosis after treatment with an anti-CD20 monoclonal antibody and alkylating agent within 6 months of initial FL diagnosis. 3L+, third line or later; ASCT, autologous stem cell transplantation; CI, confidence interval; DOR, duration of response; FL, follicular lymphoma; FLIPI, Follicular Lymphoma International Prognostic Index; GCSF, granulocyte colony-stimulating factor; IRC, independent review committee; LDC, lymphodepleting chemotherapy; LDH, lactate dehydrogenase; mGELF, modified Groupe d'Etude des Lymphomes Folliculaires; NA, not available; PD, progressive disease; PI3Ki, phosphoinositide 3-kinase inhibitor; POD24, progression of disease ≤24 months; R2, lenalidomide plus rituximab; SD, stable disease; ULN, upper limit of normal.

Supplementary Fig. 6 | Forest plot of 12-month continued response rate (DOR) per IRC assessment in 2L+ FL (efficacy set).

	Evaluable Patients (no.)	Patients With Response (no.)	12-Month DOR, % (95% CI)
Overall	124	120	83.4 (75.2–89.1)
**Age — yr**			
<65 yrs	78	76	87.2 (76.8–93.2)
≥65 and <70 yrs	37	35	80.0 (62.6–89.9)
≥75 yrs	9	9	66.7 (28.2–87.8)
**Sex (biological attribute)**			
Male	79	77	82.6 (71.8–89.5)
Female	45	43	84.9 (69.3–93.0)
**Race**			
White	66	65	87.3 (76.1–93.4)
Non-White	15	15	86.7 (56.4–96.5)
Asian	11	11	90.9 (50.8–98.7)
Non-Asian	70	69	86.6 (75.7–92.8)
**Ethnicity**			
Hispanic or Latino	6	5	80.0 (20.4–96.9)
Not Hispanic or Latino	86	84	85.0 (75.1–91.2)
**Region**			
North America	45	43	86.0 (71.6–93.5)
Europe	70	68	80.6 (68.1–88.6)
Japan	9	9	88.9 (43.3–98.4)
**FLIPI at screening**			
Low (0–1)	21	21	90.2 (66.2–97.5)
Intermediate (2)	37	37	90.9 (74.3–97.0)
High (3–5)	66	62	77.1 (64.3–85.7)
**mGELF criteria**			
Yes	70	66	81.0 (68.9–88.8)
No	54	54	86.3 (73.4–93.3)
**Bulky disease at screening[b]**			
Yes	41	40	84.0 (67.6–92.5)
No	83	80	83.1 (72.6–89.9)
**LDH at pre-LDC**			
≤ULN	73	71	91.1 (81.2–95.9)
>ULN	51	49	72.3 (57.0–82.9)
**No. of prior systemic lines of therapies**			
1	23	22	89.8 (64.8–97.4)
2	48	47	81.8 (66.8–90.5)
3	24	23	91.3 (69.5–97.8)
≥4	29	28	74.5 (53.7–87.0)
**Prior ASCT**			
Yes	30	28	96.4 (77.2–99.5)
No	94	92	79.5 (69.3–86.6)
**Prior PI3Ki**			
Yes	24	22	67.6 (43.7–83.1)
No	100	98	87.0 (78.2–92.4)
**Prior R2**			
Yes	20	19	77.8 (51.1–91.0)
No	104	101	84.6 (75.7–90.4)
**Prior bendamustine**			
None	59	57	83.0 (69.7–90.8)
≤6 months before leukapheresis	3	3	N/A
>6 and ≤12 months before leukapheresis	5	5	40.0 (5.2–75.3)
>12 months before leukapheresis	57	55	88.8 (76.7–94.8)
**Bridging therapy**			
Yes	45	42	87.6 (72.7–94.7)
No	79	78	81.2 (70.2–88.4)
**Relapsed vs. refractory**			
Relapsed	87	85	85.3 (75.5–91.4)
Refractory	37	35	78.8 (60.4–89.3)
**PD on last line of therapy or ≤6 months after last line**			
Yes	76	72	79.6 (67.9–87.4)
No	48	48	89.6 (76.8–95.5)
**PD or SD to last line of therapy or ≤6 months after last line**			
Yes	79	75	79.0 (67.5–86.8)
No	45	45	91.1 (78.0–96.6)
**Double refractory (anti-CD20 + alkylator)**			
Yes	77	73	76.9 (65.0–85.2)
No	47	47	93.6 (81.5–97.9)
**Double refractory[c]**			
Yes	49	46	73.4 (57.9–83.9)
No	75	74	89.9 (79.8–95.1)
**Double refractory[d]**			
Yes	86	82	79.7 (69.0–87.1)
No	38	38	91.7 (76.5–97.3)
**POD24 from initial immunochemotherapy[e]**			
Yes	71	68	78.0 (65.5–86.4)
No	52	51	92.1 (80.2–96.9)
**POD24 from diagnosis[f]**			
Yes	56	54	74.5 (60.0–84.5)
No	68	66	90.6 (80.2–95.7)
**GCSF**			
Yes	60	57	83.7 (70.9–91.2)
No	64	63	83.0 (70.5–90.5)

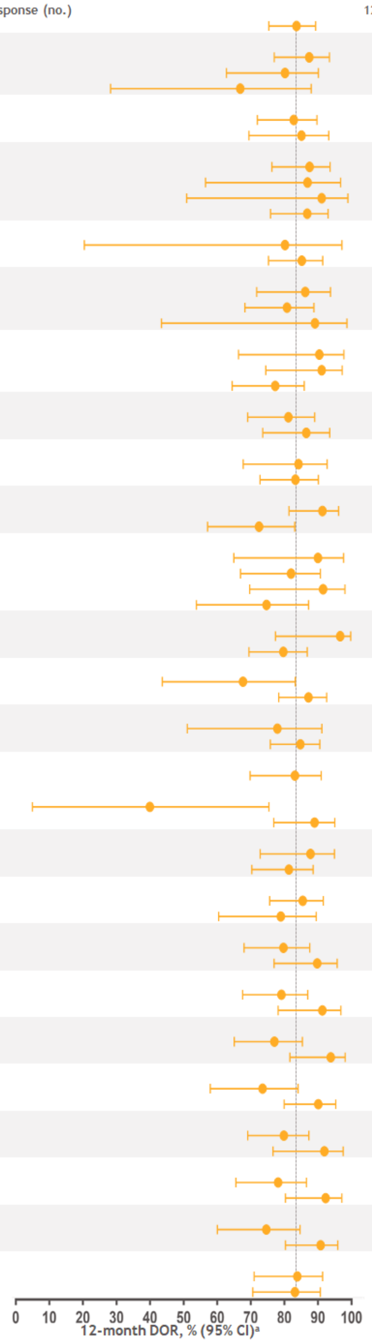

Point estimates of DOR — 95% CI based on the efficacy-evaluable population

[a]Based on Kaplan–Meier estimates. DOR was defined as the interval from the first documentation of response to PD or death from any cause, whichever occurred first. [b]Bulky disease was defined as any mass >7 cm, or 3 or more masses (each >3 cm) at screening based on investigator assessment. [c]Patients who did not respond or progressed during or up to 6 months after completing combination treatment with an anti-CD20 monoclonal antibody and alkylating agent. [d]Patients who did not respond or progressed during or up to 6 months after completing maintenance treatment with an anti-CD20 monoclonal antibody. Anti-CD20 maintenance defined as patients who completed an immediate course of combination treatment with anti-CD20 and alkylating agent. Assessment of SD or PD from start of anti-CD20 maintenance therapy up to and including 6 months after completion of anti-CD20 maintenance therapy. [e]FL progression within 24 months of first-line therapy with anti-CD20 antibody and alkylator. [f]Progression of disease within 24 months of diagnosis after treatment with an anti-CD20 monoclonal antibody and alkylating agent within 6 months of initial FL diagnosis. 2L+, second line or later; ASCT, autologous stem cell transplantation; CI, confidence interval; DOR, duration of response; FL, follicular lymphoma; FLIPI, Follicular Lymphoma International Prognostic Index; GCSF, granulocyte colony-stimulating factor; IRC, independent review committee; LDC, lymphodepleting chemotherapy; LDH, lactate dehydrogenase; mGELF, modified Groupe d'Etude des Lymphomes Folliculaires; NA, not available; PD, progressive disease; PI3Ki, phosphoinositide 3-kinase inhibitor; POD24, progression of disease ≤24 months; R2, lenalidomide plus rituximab; SD, stable disease; ULN, upper limit of normal.

Supplementary Fig. 7 | Forest plot of 12-month PFS rate per IRC assessment in 3L+ FL (efficacy set).

	Evaluable Patients (no.)	Patients With Events (no.)	12-Month PFS Rate, % (95% CI)
Overall	101	23	80.7 (71.4–87.2)
Age — yr			
<65 yrs	60	10	86.2 (74.2–92.9)
≥65 and <70 yrs	32	10	74.9 (56.0–86.6)
≥75 yrs	9	3	66.7 (28.2–87.8)
Sex (biological attribute)			
Male	62	15	80.3 (67.9–88.3)
Female	39	8	81.3 (64.7–90.6)
Race			
White	57	11	83.6 (70.9–91.1)
Non-White	12	2	83.3 (48.2–95.6)
Asian	9	1	88.9 (43.3–98.4)
Non-Asian	60	12	82.8 (70.3–90.4)
Ethnicity			
Hispanic or Latino	5	2	60.0 (12.6–88.2)
Not Hispanic or Latino	71	15	82.6 (71.4–89.7)
Region			
North America	35	9	80.0 (62.6–89.9)
Europe	58	13	80.0 (66.8–88.4)
Japan	8	1	87.5 (38.7–98.1)
FLIPI at screening			
Low (0–1)	10	0	100.0 (100.0–100.0)
Intermediate (2)	33	4	93.8 (77.3–98.4)
High (3–5)	58	19	70.4 (56.8–80.5)
mGELF criteria			
Yes	54	16	70.9 (56.3–81.4)
No	47	7	91.4 (78.8–96.7)
Bulky disease at screening[b]			
Yes	31	7	76.1 (56.1–87.9)
No	70	16	82.7 (71.5–89.8)
LDH at pre-LDC			
≤ULN	56	10	89.3 (77.7–95.0)
>ULN	45	13	70.0 (54.0–81.4)
No. of prior systemic lines of therapies			
1	0	0	NA
2	48	10	82.7 (68.3–91.0)
3	24	3	87.5 (66.1–95.8)
≥4	29	10	71.9 (51.5–84.9)
Prior ASCT			
Yes	30	3	90.0 (72.1–96.7)
No	71	20	76.8 (64.9–85.1)
Prior PI3Ki			
Yes	24	10	62.0 (39.6–78.1)
No	77	13	86.7 (76.6–92.6)
Prior R2			
Yes	20	7	73.7 (47.9–88.1)
No	81	16	82.5 (72.3–89.3)
Prior bendamustine			
None	42	9	82.6 (66.8–91.3)
≤6 months before leukapheresis	2	1	NA
>6 and ≤12 months before leukapheresis	3	3	NA
>12 months before leukapheresis	54	10	84.8 (71.9–92.1)
Bridging therapy			
Yes	40	8	82.1 (65.9–91.0)
No	61	15	79.7 (67.0–87.9)
Relapsed vs. refractory			
Relapsed	67	13	83.1 (71.6–90.3)
Refractory	34	10	75.7 (57.2–87.0)
PD on last line of therapy or ≤6 months after last line			
Yes	61	18	76.4 (63.3–85.3)
No	40	5	87.5 (72.5–94.6)
PD or SD to last line of therapy or ≤6 months after last line			
Yes	64	19	75.8 (63.0–84.6)
No	37	4	89.2 (73.7–95.8)
Double refractory (anti-CD20 + alkylator)			
Yes	66	19	74.9 (62.2–83.8)
No	35	4	91.4 (75.7–97.2)
Double refractory[c]			
Yes	40	13	69.7 (52.9–81.6)
No	61	10	88.1 (76.6–94.1)
Double refractory[d]			
Yes	71	19	77.1 (65.4–85.3)
No	30	4	89.4 (70.5–96.5)
POD24 from initial immunochemotherapy[e]			
Yes	56	16	76.1 (62.4–85.4)
No	44	6	88.6 (74.8–95.1)
POD24 from diagnosis[f]			
Yes	44	14	74.7 (58.9–85.1)
No	57	9	85.5 (73.1–92.5)
GCSF			
Yes	50	11	77.4 (62.8–86.8)
No	51	12	83.8 (70.2–91.6)

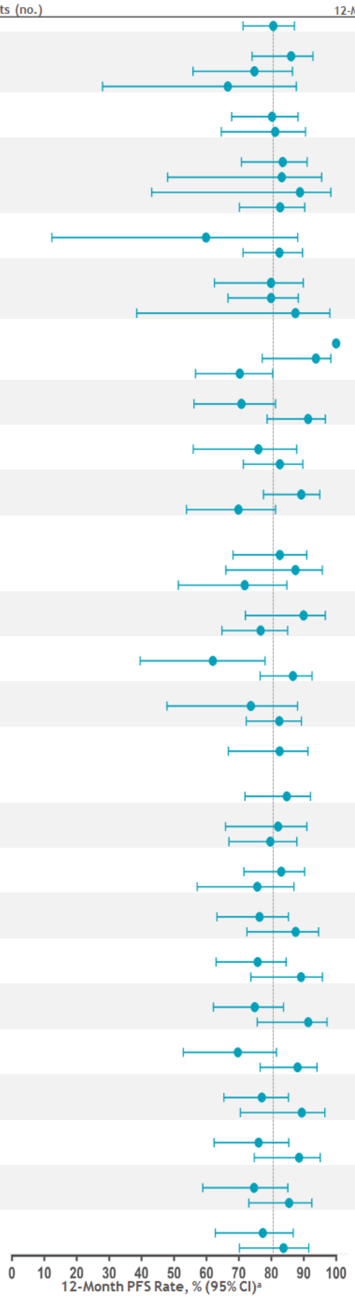

● Point estimates of PFS rate ├─┤ 95% CI based on the efficacy-evaluable population

[a]Based on Kaplan–Meier estimates. PFS was defined as the interval from the date of liso-cel infusion to PD or death due to any cause, whichever occurred first. [b]Bulky disease was defined as any mass >7 cm, or 3 or more masses (each >3 cm) at screening based on investigator assessment. [c]Patients who did not respond or progressed during or up to 6 months after completing combination treatment with an anti-CD20 monoclonal antibody and alkylating agent. [d]Patients who did not respond or progressed during or up to 6 months after completing maintenance treatment with an anti-CD20 monoclonal antibody. Anti-CD20 maintenance defined as patients who completed an immediate course of combination treatment with anti-CD20 and alkylating agent. Assessment of SD or PD from start of anti-CD20 maintenance therapy up to and including 6 months after completion of anti-CD20 maintenance therapy. [e]FL progression within 24 months of first-line therapy with anti-CD20 antibody and alkylator. [f]Progression of disease within 24 months of diagnosis after treatment with an anti-CD20 monoclonal antibody and alkylating agent within 6 months of initial FL diagnosis. 3L+, third line or later; ASCT, autologous stem cell transplantation; CI, confidence interval; FL, follicular lymphoma; FLIPI, Follicular Lymphoma International Prognostic Index; GCSF, granulocyte colony-stimulating factor; IRC, independent review committee; liso-cel, lisocabtagene maraleucel; LDC, lymphodepleting chemotherapy; LDH, lactate dehydrogenase; mGELF, modified Groupe d'Etude des Lymphomes Folliculaires; NA, not available; PD, progressive disease; PFS, progression-free survival; PI3Ki, phosphoinositide 3-kinase inhibitor; POD24, progression of disease ≤24 months; R2, lenalidomide plus rituximab; SD, stable disease; ULN, upper limit of normal.

Supplementary Fig. 8 | Forest plot of 12-month PFS rate per IRC assessment in 2L+ FL (efficacy set).

	Evaluable Patients (no.)	Patients With Event (no.)	12-Month PFS Rate, % (95% CI)
Overall	124	27	82.7 (74.7–88.4)
Age — yr			
<65 yrs	78	13	88.2 (78.5–93.7)
≥65 and <70 yrs	37	11	75.6 (58.3–86.5)
≥75 yrs	9	3	66.7 (28.2–87.8)
Sex (biological attribute)			
Male	79	17	82.1 (71.6–89.0)
Female	45	10	83.9 (69.2–92.0)
Race			
White	66	11	85.9 (74.7–92.4)
Non-White	15	2	86.7 (56.4–96.5)
Asian	11	1	90.9 (50.8–98.7)
Non-Asian	70	12	85.3 (74.4–91.8)
Ethnicity			
Hispanic or Latino	6	2	66.7 (19.5–90.4)
Not Hispanic or Latino	86	17	84.6 (74.9–90.7)
Region			
North America	45	11	82.2 (67.6–90.7)
Europe	70	15	82.1 (70.7–89.5)
Japan	9	1	88.9 (43.3–98.4)
FLIPI at screening			
Low (0–1)	21	3	95.2 (70.7–99.3)
Intermediate (2)	37	4	94.4 (79.6–98.6)
High (3–5)	66	20	72.5 (59.9–81.7)
mGELF criteria			
Yes	70	17	76.4 (64.3–84.8)
No	54	10	90.7 (79.1–96.0)
Bulky disease at screening[b]			
Yes	41	7	82.1 (66.0–91.1)
No	83	20	83.0 (73.0–89.6)
LDH at pre-LDC			
≤ULN	73	12	90.4 (80.9–95.3)
>ULN	51	15	71.7 (56.9–82.2)
No. of prior systemic lines of therapies			
1	23	4	91.3 (69.5–97.8)
2	48	10	82.7 (68.3–91.0)
3	24	3	87.5 (66.1–95.8)
≥4	29	10	71.9 (51.5–84.9)
Prior ASCT			
Yes	30	3	90.0 (72.1–96.7)
No	94	24	80.5 (70.8–87.2)
Prior PI3Ki			
Yes	24	10	62.0 (39.6–78.1)
No	100	17	87.8 (79.5–92.9)
Prior R2			
Yes	20	7	73.7 (47.9–88.1)
No	104	20	84.5 (75.9–90.2)
Prior bendamustine			
None	59	13	84.3 (72.0–91.5)
≤6 months before leukapheresis	3	1	NA
>6 and ≤12 months before leukapheresis	5	3	40.0 (5.2–75.3)
>12 months before leukapheresis	57	10	85.7 (73.3–92.6)
Bridging therapy			
Yes	45	9	81.9 (67.0–90.5)
No	79	18	83.2 (72.8–89.9)
Relapsed vs. refractory			
Relapsed	87	17	84.8 (75.2–90.9)
Refractory	37	10	77.7 (60.4–88.2)
PD on last line of therapy or ≤6 months after last line			
Yes	76	22	78.5 (67.3–86.2)
No	48	5	89.6 (76.8–95.5)
PD or SD to last line of therapy or ≤6 months after last line			
Yes	79	23	78.0 (66.9–85.7)
No	45	4	91.1 (78.0–96.6)
Double refractory (anti-CD20 + alkylator)			
Yes	77	23	75.9 (64.5–84.1)
No	47	4	93.6 (81.5–97.9)
Double refractory[c]			
Yes	49	16	71.3 (56.3–81.9)
No	75	11	90.4 (80.8–95.3)
Double refractory[d]			
Yes	86	23	78.8 (68.5–86.1)
No	38	4	91.7 (76.5–97.3)
POD24 from initial immunochemotherapy[e]			
Yes	71	20	78.4 (66.8–86.4)
No	52	6	90.3 (78.3–95.9)
POD24 from diagnosis[f]			
Yes	56	18	76.6 (63.1–85.7)
No	68	9	87.9 (77.3–93.8)
GCSF			
Yes	60	13	79.5 (66.7–87.8)
No	64	14	85.6 (74.2–92.3)

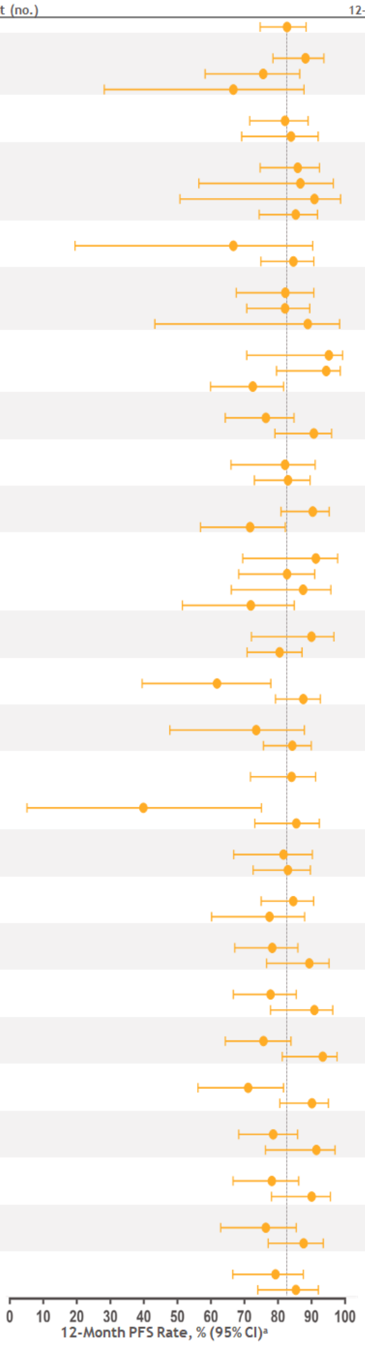

● Point estimates of PFS rate ⊢⊣ 95% CI based on the efficacy-evaluable population

[a]Based on Kaplan–Meier estimates. PFS was defined as the interval from the date of liso-cel infusion to PD or death due to any cause, whichever occurred first. [b]Bulky disease was defined as any mass >7 cm, or 3 or more masses (each >3 cm) at screening based on investigator assessment. [c]Patients who did not respond or progressed during or up to 6 months after completing combination treatment with an anti-CD20 monoclonal antibody and alkylating agent. [d]Patients who did not respond or progressed during or up to 6 months after completing maintenance treatment with an anti-CD20 monoclonal antibody. Anti-CD20 maintenance defined as patients who completed an immediate course of combination treatment with anti-CD20 and alkylating agent. Assessment of SD or PD from start of anti-CD20 maintenance therapy up to and including 6 months after completion of anti-CD20 maintenance therapy. [e]FL progression within 24 months of first-line therapy with anti-CD20 antibody and alkylator. [f]Progression of disease within 24 months of diagnosis after treatment with an anti-CD20 monoclonal antibody and alkylating agent within 6 months of initial FL diagnosis. 2L+, second line or later; ASCT, autologous stem cell transplantation; CI, confidence interval; FL, follicular lymphoma; FLIPI, Follicular Lymphoma International Prognostic Index; GCSF, granulocyte colony-stimulating factor; IRC, independent review committee; liso-cel, lisocabtagene maraleucel; LDC, lymphodepleting chemotherapy; LDH, lactate dehydrogenase; mGELF, modified Groupe d'Etude des Lymphomes Folliculaires; NA, not available; PD, progressive disease; PFS, progression-free survival; PI3Ki, phosphoinositide 3-kinase inhibitor; POD24, progression of disease ≤24 months; R2, lenalidomide plus rituximab; SD, stable disease; ULN, upper limit of normal.

Supplementary Fig. 9 | Liso-cel cellular kinetics over time in patients with 2L and 3L+ FL (cellular kinetic set[a]).

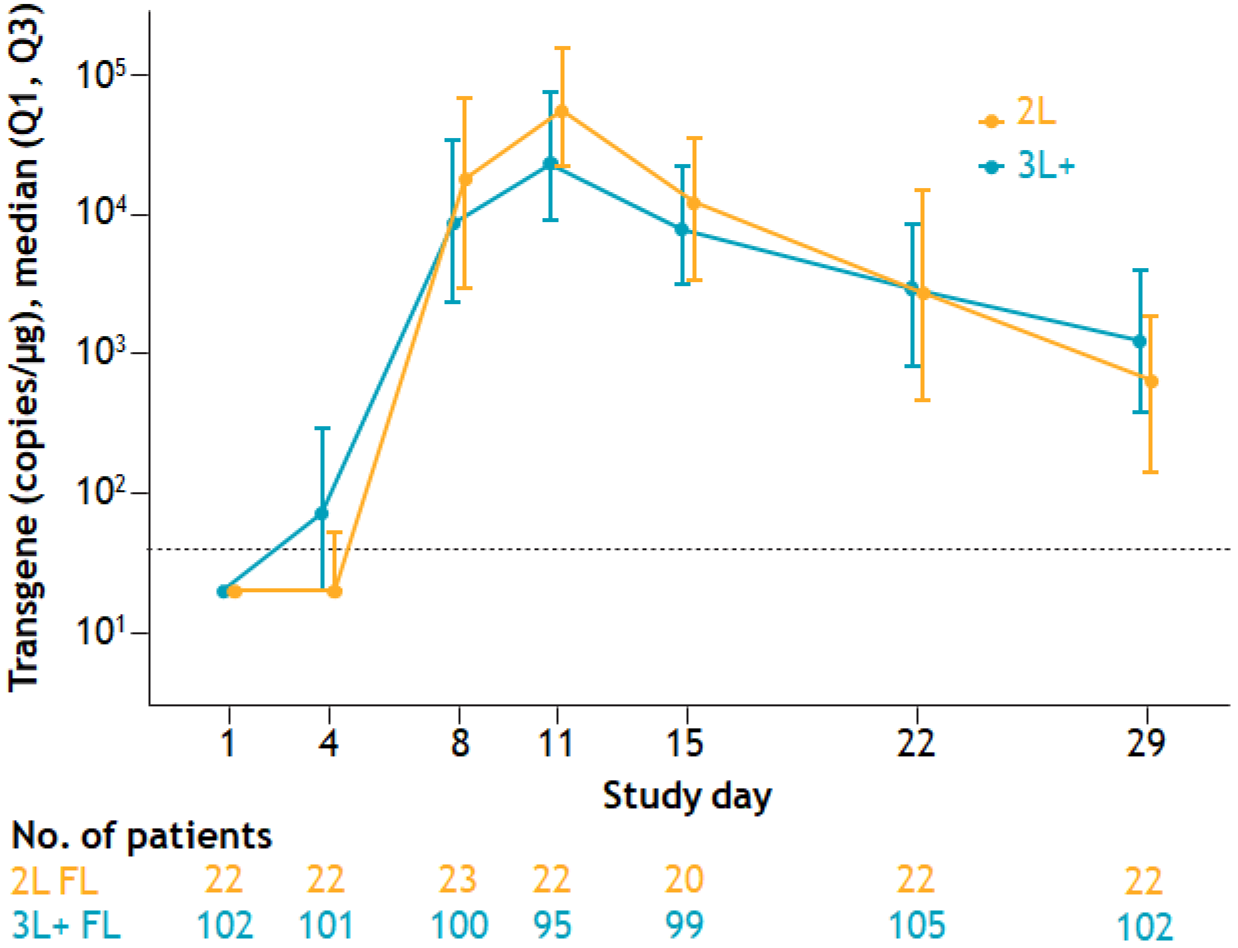

[a]Assessed in 2L and 3L+ FL liso-cel–treated set with available measurements of cellular kinetics by PCR. Transgene levels < LLOD (16 copies/reaction, which is around 40 copies/µg, denoted as dashed line) were substituted to LLOD/2. Any quantifiable values obtained before liso-cel infusion were excluded. 2L, second line; 3L+, third line or later; FL, follicular lymphoma; liso-cel, lisocabtagene maraleucel; LLOD, lower limit of detection; PCR, polymerase chain reaction; Q, quartile.

Supplementary Fig. 10 | Completion rates for EORTC QLQ-C30 (PRO analysis set).[a]

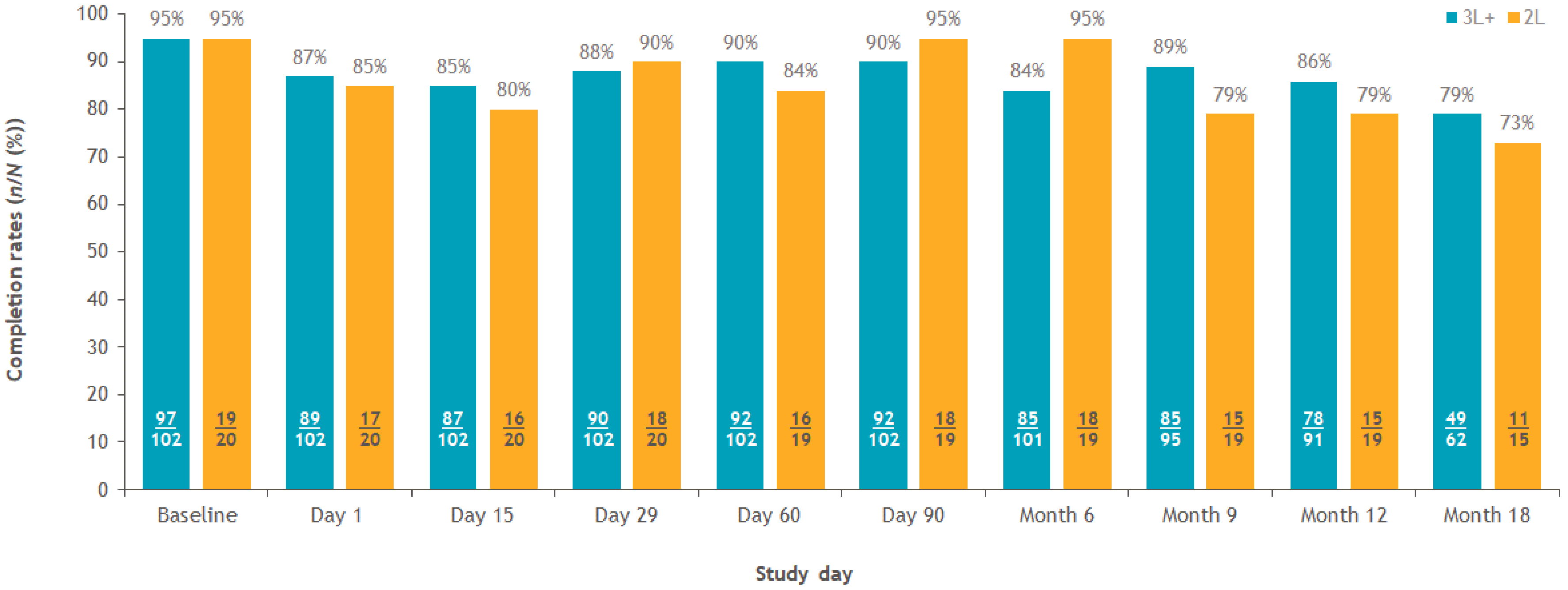

[a]Baseline was defined as the latest measurement within 7 days before LDC. 2L, second line; 3L+, third line or later; EORTC QLQ-C30, European Organisation for the Research and Treatment of Cancer Quality of Life Questionnaire-Core 30 items; LDC, lymphodepleting chemotherapy; PRO, patient-reported outcome.

Supplementary Fig. 11 | EORTC QLQ-C30 mean changes from baseline in the primary domains of interest over time (PRO analysis set).[a]

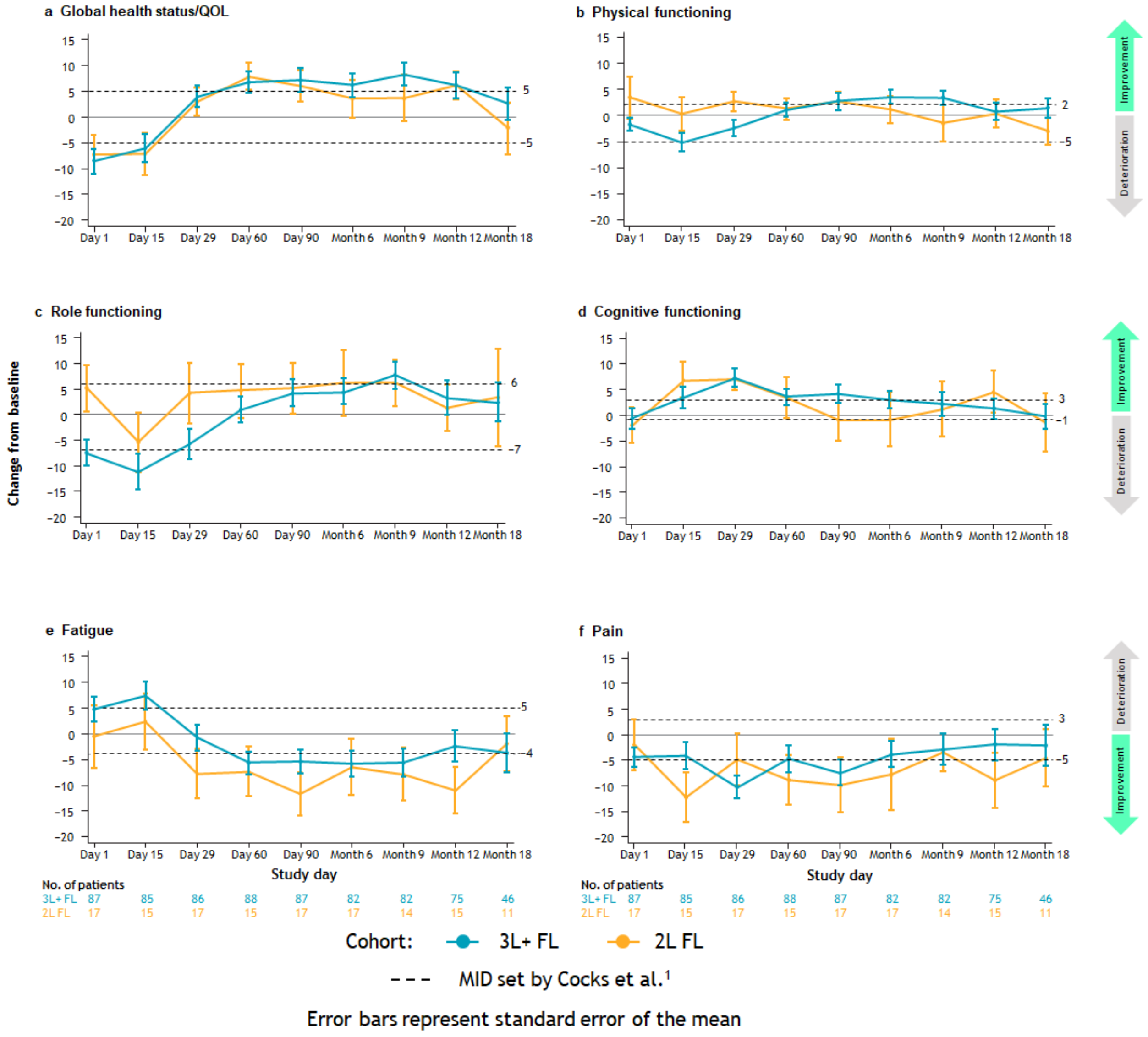

[a]Baseline was defined as the latest measurement within 7 days before LDC. The figure only included time points where data were available for ≥10 patients in each analysis group. The analysis was based on observed changes relative to baseline in primary domains from the day of liso-cel infusion (day 1) through month 18 (day 545). For symptom domains (pain and fatigue), decreases in observed mean changes from baseline indicate improvement in symptoms. 2L, second line; 3L+, third line or later; EORTC QLQ-C30, European Organisation for the Research and Treatment of Cancer Quality of Life Questionnaire-Core 30 items; FL, follicular lymphoma; LDC, lymphodepleting chemotherapy; MID, minimally important difference; PRO, patient-reported outcome.

Supplementary Fig. 12 | EORTC QLQ-C30 mean changes from baseline in the secondary domains of interest over time (PRO analysis set).[a]

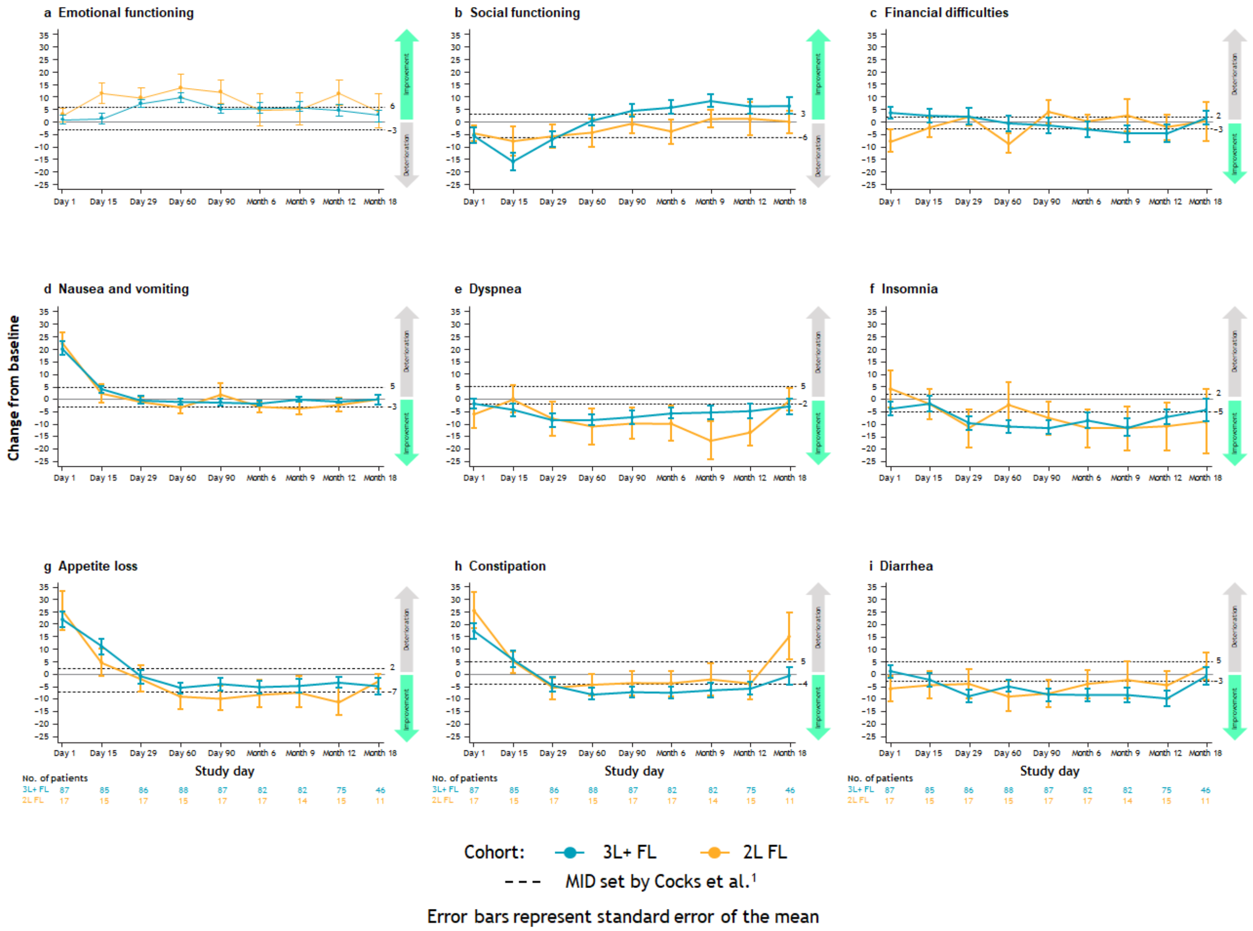

[a]Baseline was defined as the latest measurement within 7 days before LDC. The figure only included time points where data were available for ≥10 patients in each analysis group. The analysis was based on observed changes relative to baseline in secondary domains from the day of liso-cel infusion (day 1) through month 18 (day 545). For symptom domains (eg, insomnia and diarrhea), decreases in observed mean changes from baseline indicate improvement in symptoms. 2L, second line; 3L+, third line or later; EORTC QLQ-C30, European Organisation for the Research and Treatment of Cancer Quality of Life Questionnaire-Core 30 items; FL, follicular lymphoma; LDC, lymphodepleting chemotherapy; MID, minimally important difference; PRO, patient-reported outcome.

Supplementary Fig. 13 | FACT-LymS mean changes from baseline over time (PRO analysis set).[a]

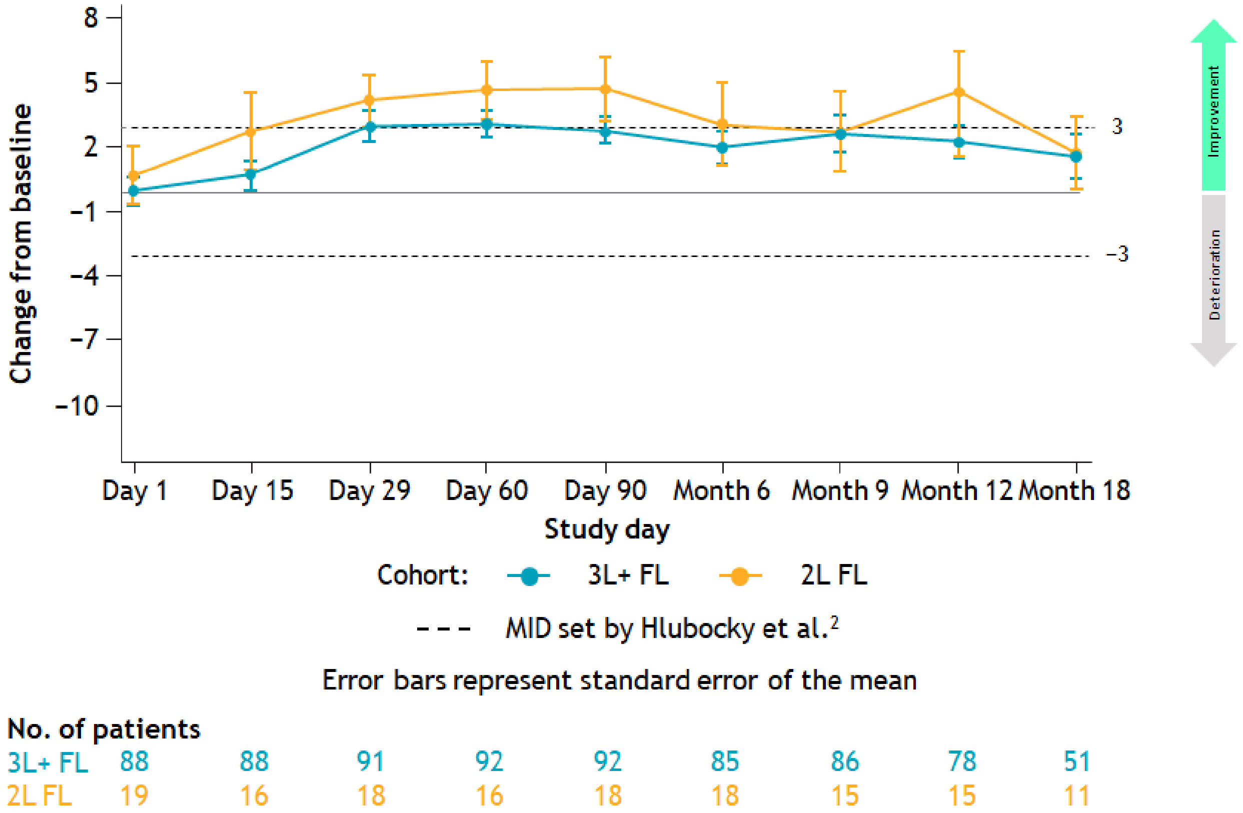

[a]Baseline was defined as the latest measurement within 7 days before LDC. At postbaseline time points, only time points where data were available for ≥10 patients in each analysis group are plotted. The analysis was based on observed changes relative to baseline in FACT-LymS from the day of liso-cel infusion (day 1) through month 18 (day 545). 2L, second line; 3L+, third line or later; FACT-LymS, Functional Assessment of Cancer Therapy-Lymphoma “Additional Concerns” Scale; FL, follicular lymphoma; LDC, lymphodepleting chemotherapy; MID, minimally important difference; PRO, patient-reported outcome.

Supplementary Fig. 14 | Time to confirmed improvement in primary domains of interest (PRO analysis set).[a]

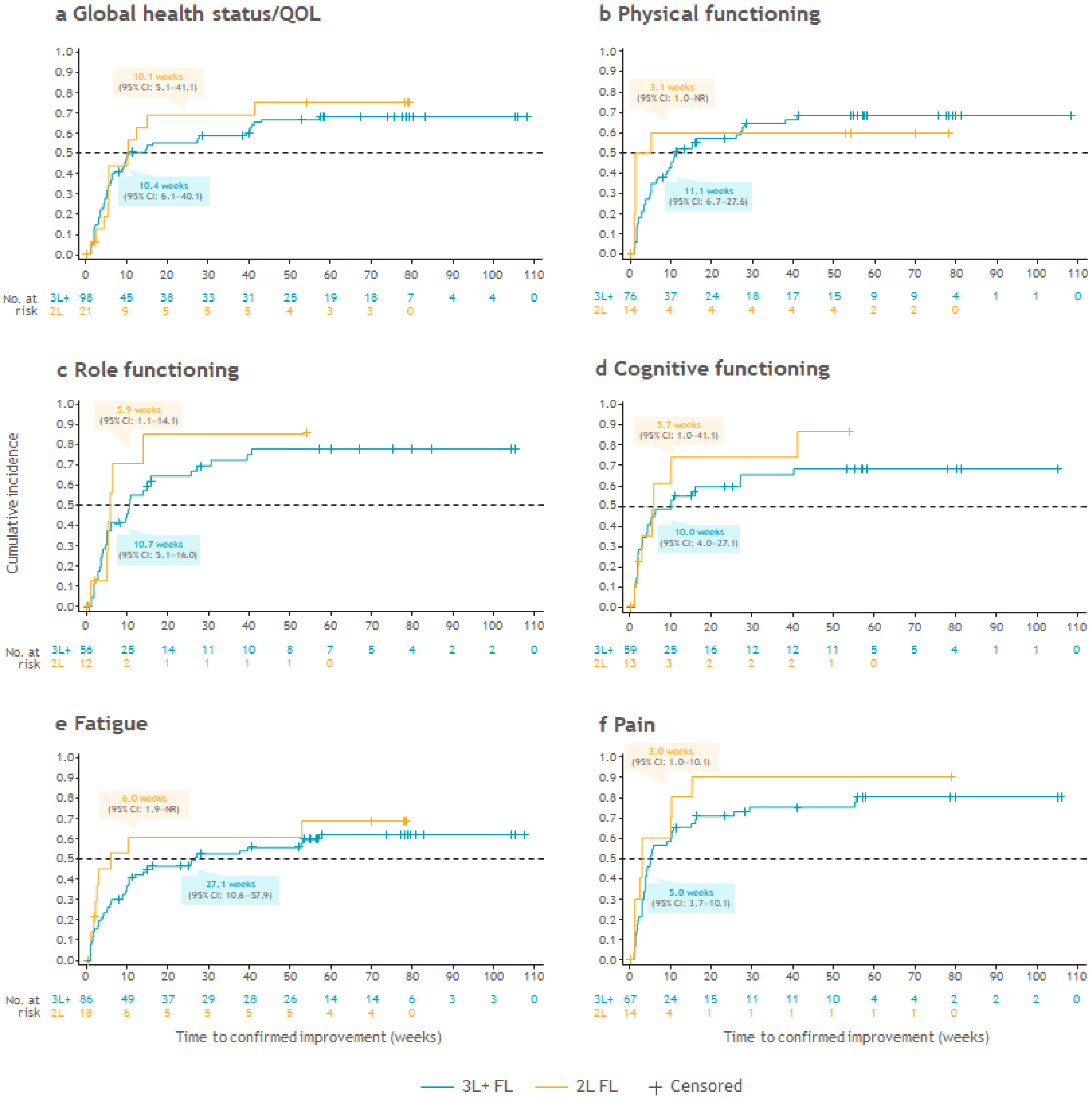

[a]Time to confirmed improvement was defined as time to the first of ≥2 consecutive assessments with a PRO score change greater than or equal to the responder definition for improvement. 2L, second line; 3L+, third line or later; CI, confidence interval; FL, follicular lymphoma; PRO, patient-reported outcome; QOL, quality of life; NR, not reached.

Supplementary Fig. 15 | Time to confirmed improvement in FACT-LymS (PRO analysis set).[a]

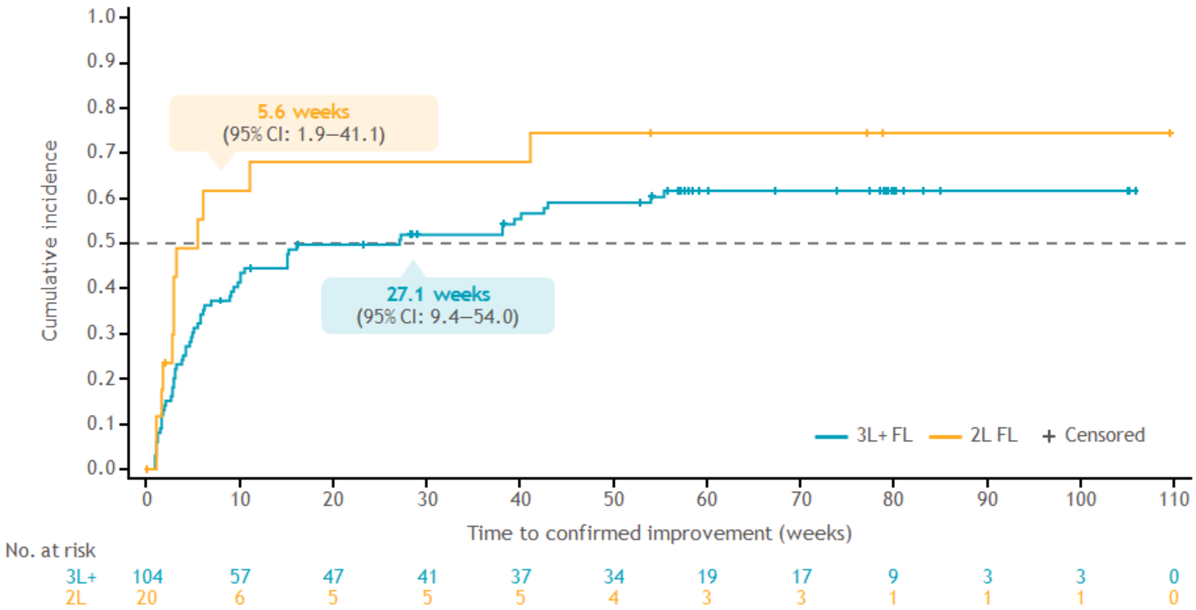

[a]Time to confirmed improvement was defined as time to the first of ≥2 consecutive assessments with a PRO score change greater than or equal to the responder definition for improvement. 2L, second line; 3L+, third line or later; CI, confidence interval; FACT-LymS, Functional Assessment of Cancer Therapy-Lymphoma “Additional Concerns” Scale; FL, follicular lymphoma; PRO, patient-reported outcome.

Supplementary Fig. 16 | Proportion of patients with clinically meaningful change in the EORTC QLQ-C30 primary domains of interest (PRO analysis set).[a]

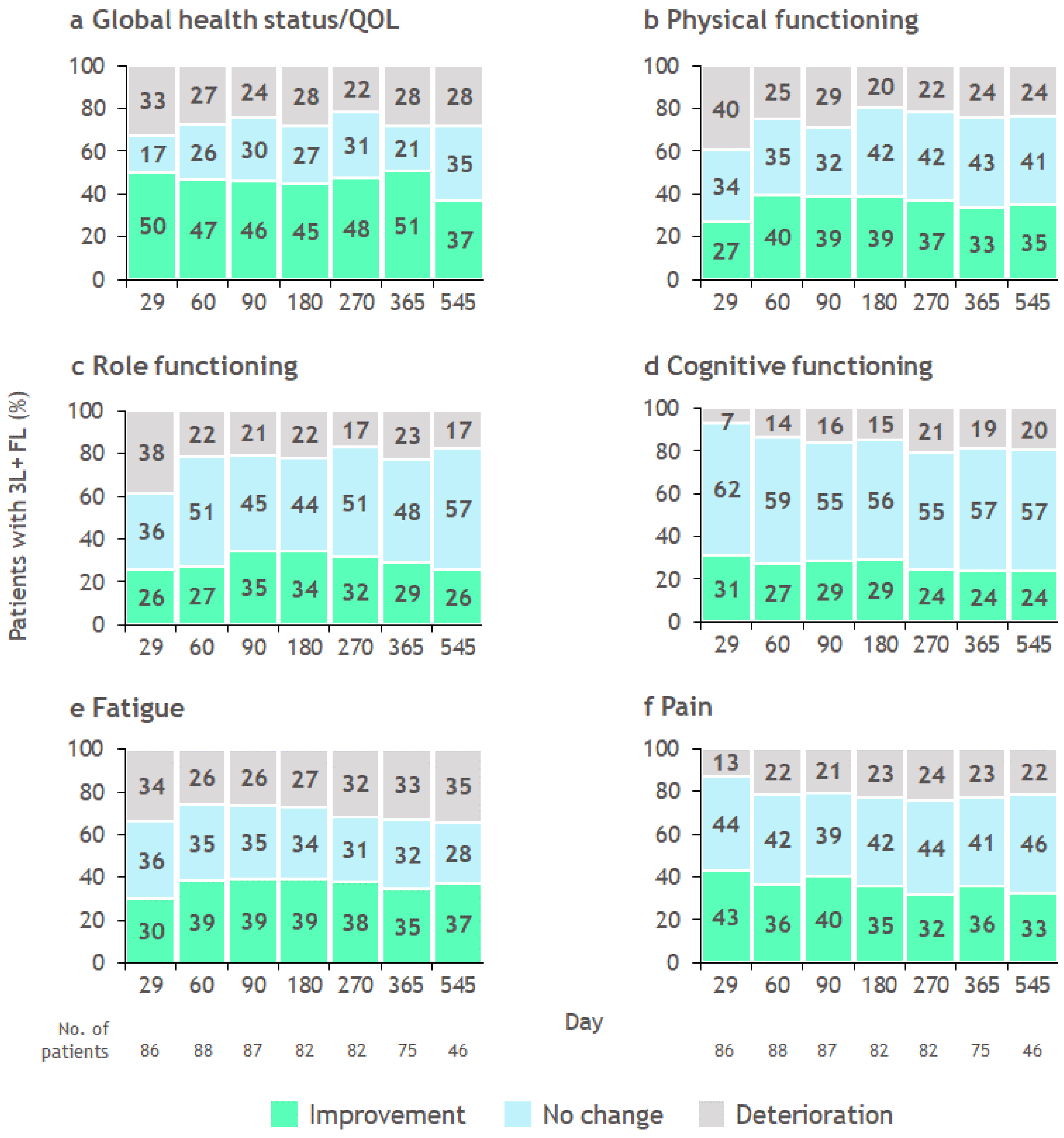

[a]Improvement, no change, and worsening were defined by score changes from baseline of ≥5, <5 to >−5, and ≤−5 for global health status/QOL and physical functioning; ≤−10, <10 to >−10, and ≥10 for fatigue; ≤−15, <15 to >−15, and ≥15 for pain; and ≥15, <15 to >−15, and ≤−15 for cognitive functioning and role functioning, respectively. 3L+, third line or later; EORTC QLQ-C30, European Organisation for Research and Treatment of Cancer Quality of life Questionnaire-Core 30 items; FL, follicular lymphoma; PRO, patient-reported outcome; QOL, quality of life.

Supplementary Fig. 17 | Proportion of patients with clinically meaningful change in FACT-LymS (PRO analysis set).[a]

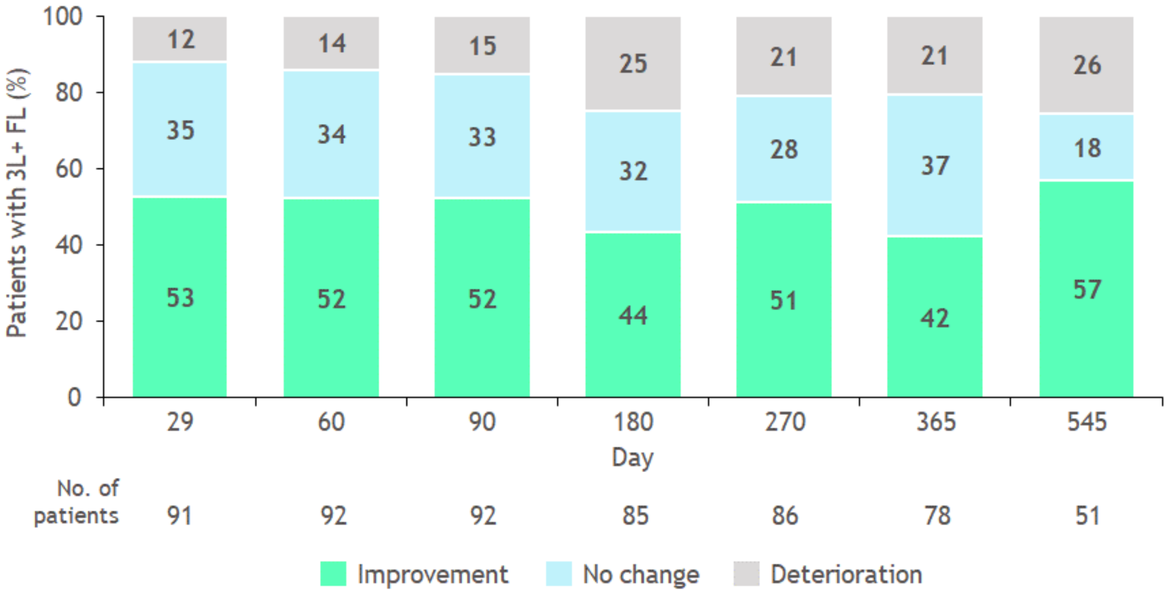

[a]Improvement, no change, and worsening were defined by score changes from baseline of ≥3, <3 to >−3, and ≤−3, respectively. The denominator for the percentage calculation in each category is based on the number of patients who answered ≥50% of the items for the domain. 3L+, third line or later; FACT-LymS, Functional Assessment of Cancer Therapy-Lymphoma “Additional Concerns” Scale; FL, follicular lymphoma; PRO, patient-reported outcome.

Supplementary Fig. 18 | Hierarchical hypothesis testing.[a]

a Sequence 1 **b Sequence 2**

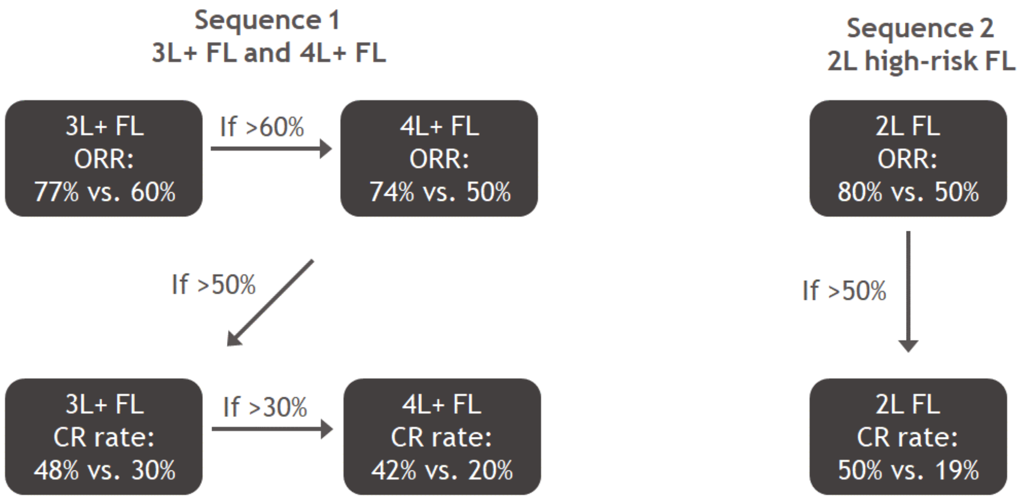

Statistically tested R/R FL analysis groups (name)	FL cohorts
Analysis group 1 (4L+ R/R FL)	4L+ FL
Analysis group 2 (3L+ R/R FL)	4L+ FL and 3L FL
Analysis group 3 (2L R/R FL)	2L FL

[a]Hypothesis testing was performed for the primary and key secondary endpoints in two sequences based on prespecified analysis groups comprising FL cohorts. Hierarchical testing, or gating, was used to control for type I error for multiple endpoints, with endpoints tested in the order of ORR followed by CR rate. Null hypotheses (noted parenthetically) were tested at one-sided $\alpha=0.025$ significance. Sequence 1 was tested in the following order: 3L+ FL (ORR ≤60%), 4L+ FL (ORR ≤50%), 3L+ FL (CR rate ≤30%), and 4L+ FL (CR rate ≤20%). Sequence 2 was tested in the following order: 2L (ORR ≤50%) and 2L (CR rate ≤19%). The primary analysis for 4L+ R/R FL, 3L+ R/R FL, and 2L R/R FL analysis groups was planned when approximately 50, 90, and 20 patients, respectively, had been followed for duration of response for approximately 12 months after first response per investigator's assessment or until death, PD, or study withdrawal. The study utilized the following sample size calculations for power analyses using one-sided 0.025 level testing: with a sample size of 50 treated patients in the 4L+ FL cohort, there would be 90% power to detect an ORR of 74% versus 50%, or a CR rate of 42% versus 20%; with a sample size of 90 patients with 3L+ FL (4L+ FL and 3L FL cohorts), there would be 90% power to detect an ORR of 77% versus 60%, or a CR rate of 48% versus 30%; with a sample size of 20 treated patients with 2L FL, there would be 80% power to detect an ORR of 80% versus 50%, or a CR rate of 50% versus 19%. 2L, second line; 3L, third line; 3L+, third line or later; 4L+, fourth line or later; CR, complete response; FL, follicular lymphoma; ORR, overall response rate; PD, progressive disease; R/R, relapsed/refractory.

References

1. Cocks, K., *et al.* Evidence-based guidelines for interpreting change scores for the European Organisation for the Research and Treatment of Cancer Quality of Life Questionnaire Core 30. *Eur. J. Cancer* **48**, 1713–1721 (2012).
2. Hlubocky, F.J., *et al.* A preliminary study of a health related quality of life assessment of priority symptoms in advanced lymphoma: the National Comprehensive Cancer Network-Functional Assessment of Cancer Therapy - Lymphoma Symptom Index. *Leuk. Lymphoma* **54**, 1942–1946 (2013).
