# Supplementary material for: Lisocabtagene maraleucel in follicular lymphoma: the phase 2 TRANSCEND FL study
Source: Nat Med. 2024 Jun 3;30(8):2199–207. doi: 10.1038/s41591-024-02986-9 (PMC11333271; doi:10.1038/s41591-024-02986-9)
Supplement: Supplementary file 2 — Reporting Summary [file 41591_2024_2986_MOESM2_ESM.pdf]

nature portfolio

Corresponding author(s): Franck Morschhauser

Last updated by author(s): April, 2024

Reporting Summary

Nature Portfolio wishes to improve the reproducibility of the work that we publish. This form provides structure for consistency and transparency in reporting. For further information on Nature Portfolio policies, see our Editorial Policies and the Editorial Policy Checklist.

Please do not complete any field with "not applicable" or n/a. Refer to the help text for what text to use if an item is not relevant to your study.
For final submission: please carefully check your responses for accuracy; you will not be able to make changes later.

Statistics

For all statistical analyses, confirm that the following items are present in the figure legend, table legend, main text, or Methods section.

n/a	Confirmed	
☐	☑	The exact sample size (*n*) for each experimental group/condition, given as a discrete number and unit of measurement
☒	☐	A statement on whether measurements were taken from distinct samples or whether the same sample was measured repeatedly
☐	☑	The statistical test(s) used AND whether they are one- or two-sided<br>*Only common tests should be described solely by name; describe more complex techniques in the Methods section.*
☒	☐	A description of all covariates tested
☒	☐	A description of any assumptions or corrections, such as tests of normality and adjustment for multiple comparisons
☐	☑	A full description of the statistical parameters including central tendency (e.g. means) or other basic estimates (e.g. regression coefficient) AND variation (e.g. standard deviation) or associated estimates of uncertainty (e.g. confidence intervals)
☐	☑	For null hypothesis testing, the test statistic (e.g. *F*, *t*, *r*) with confidence intervals, effect sizes, degrees of freedom and *P* value noted<br>*Give P values as exact values whenever suitable.*
☒	☐	For Bayesian analysis, information on the choice of priors and Markov chain Monte Carlo settings
☐	☑	For hierarchical and complex designs, identification of the appropriate level for tests and full reporting of outcomes
☒	☐	Estimates of effect sizes (e.g. Cohen's *d*, Pearson's *r*), indicating how they were calculated

Our web collection on statistics for biologists contains articles on many of the points above.

Software and code

Policy information about availability of computer code

Data collection	Data collection was performed using the Bristol Myers Squibb Rave Electronic Data Capture platform.
Data analysis	Please see Methods, sections Study endpoints and Statistical analysis in the manuscript, pages 36-39

For manuscripts utilizing custom algorithms or software that are central to the research but not yet described in published literature, software must be made available to editors and reviewers. We strongly encourage code deposition in a community repository (e.g. GitHub). See the Nature Portfolio guidelines for submitting code & software for further information.

Data

Policy information about availability of data

All manuscripts must include a data availability statement. This statement should provide the following information, where applicable:

- Accession codes, unique identifiers, or web links for publicly available datasets
- A description of any restrictions on data availability
- For clinical datasets or third party data, please ensure that the statement adheres to our policy

Bristol Myers Squibb policy on data sharing may be found at https://www.bms.com/researchers-and-partners/independent-research/data-sharing-request-process.html.

Research involving human participants, their data, or biological material

Policy information about studies with human participants or human data. See also policy information about sex, gender (identity/presentation), and sexual orientation and race, ethnicity and racism.

Reporting on sex and gender	Provided in Table 1
Reporting on race, ethnicity, or other socially relevant groupings	Provided in Table 1
Population characteristics	Provided in Table 1
Recruitment	This is a single-arm, open label study with no comparator arm where selection bias is less relevant.<br>All advertisements used for patient recruitment were reviewed by study sponsor and institutional review boards and ethics committees.
Ethics oversight	Provided in the Methods section, page 33, Study oversight

Note that full information on the approval of the study protocol must also be provided in the manuscript.

Field-specific reporting

Please select the one below that is the best fit for your research. If you are not sure, read the appropriate sections before making your selection.

☑ Life sciences ☐ Behavioural & social sciences ☐ Ecological, evolutionary & environmental sciences

For a reference copy of the document with all sections, see nature.com/documents/nr-reporting-summary-flat.pdf

Life sciences study design

All studies must disclose on these points even when the disclosure is negative.

Sample size	Provided in the Methods section, page 38, Statistical analysis<br>Assuming a 20% dropout rate, a planned sample size of approximately 138 patients with iNHL would ensure approximately 110 patients with FL (4L+, n=50; 3L, n=40; 2L, n=20) were treated with liso-cel. With this sample size, using exact binomial one-sample tests unadjusted for the hierarchical testing procedure, there would be 90% power in 4L+ FL and 3L+ FL and 80% power in 2L to detect improvements in ORR and CR rate endpoints versus defined thresholds (Supplementary Fig. 18). Specifically, for patients with 4L+ FL, the null hypotheses were ≤50% for ORR and ≤20% for CR rate, as assessed by PET/CT. With a sample size of 50 treated patients, using one-sided 0.025 level testing, there would be 90% power to detect an ORR of 74% versus 50%, or a CR rate of 42% versus 20%. For patients with 3L+ FL (i.e., 4L+ FL and 3L FL), the null hypotheses were ≤60% for ORR and ≤30% for CR rate. With a sample size of 90 patients, using one-sided 0.025 level testing, there would be 90% power to detect an ORR of 77% versus 60%, or a CR rate of 48% versus 30%. For patients with 2L FL, the null hypotheses were ≤50% for ORR and ≤19% for CR rate. The analysis of this cohort was for proof of concept. With a sample size of 20 treated patients, using one-sided 0.025 level testing, there would be 80% power to detect an ORR of 80% versus 50%, or a CR rate of 50% versus 19%. Efficacy and safety results in 2L+ FL were reported with descriptive statistics with no predefined testing hypothesis.
Data exclusions	Eligibility criteria for patients were pre-established. No data were excluded.
Replication	This is a clinical study with human patients. It cannot be replicated as fundamental biological experiments.
Randomization	n/a There is no established standard of care for patients with 2L FL with high-risk features. It is difficult to find a reasonable comparator. Enrollment in a randomized controlled trial (RCT) may be difficult considering ongoing RCTs with commercially-available CAR T/bispecfic products in R/R FL.A randomized control trial design would ideally/ethically include SOC cross-over to liso-cel/commercially-available CAR T product, and it would be difficult to interpret the specific effect of liso-cel on overall survival, which is an important long-term outcome. There are expansive safety data for liso-cel treatment in large B-cell lymphoma supporting a single-arm, open-label design.
Blinding	This is a single-arm, open-label study with no comparator. No blinding was performed.

Behavioural & social sciences study design

All studies must disclose on these points even when the disclosure is negative.

Study description	
Research sample	
Sampling strategy	
Data collection	
Timing	
Data exclusions	
Non-participation	
Randomization	

Ecological, evolutionary & environmental sciences study design

All studies must disclose on these points even when the disclosure is negative.

Study description	
Research sample	
Sampling strategy	
Data collection	
Timing and spatial scale	
Data exclusions	
Reproducibility	
Randomization	
Blinding	

Did the study involve field work? ☐ Yes ☐ No

Field work, collection and transport

Field conditions	
Location	
Access & import/export	
Disturbance	

Reporting for specific materials, systems and methods

We require information from authors about some types of materials, experimental systems and methods used in many studies. Here, indicate whether each material, system or method listed is relevant to your study. If you are not sure if a list item applies to your research, read the appropriate section before selecting a response.

Materials & experimental systems

n/a	Involved in the study	
x	☐	Antibodies
x	☐	Eukaryotic cell lines
x	☐	Palaeontology and archaeology
x	☐	Animals and other organisms
☐	✓	Clinical data
x	☐	Dual use research of concern
x	☐	Plants

Methods

n/a	Involved in the study	
x	☐	ChIP-seq
☐	☐	Flow cytometry
☐	☐	MRI-based neuroimaging

Antibodies

Antibodies used	
Validation	

Eukaryotic cell lines

Policy information about cell lines and Sex and Gender in Research

Cell line source(s)	
Authentication	
Mycoplasma contamination	
Commonly misidentified lines<br>(See ICLAC register)	

Palaeontology and Archaeology

Specimen provenance	
Specimen deposition	
Dating methods	

☐ Tick this box to confirm that the raw and calibrated dates are available in the paper or in Supplementary Information.

Ethics oversight	

Note that full information on the approval of the study protocol must also be provided in the manuscript.

Animals and other research organisms

Policy information about studies involving animals; ARRIVE guidelines recommended for reporting animal research, and Sex and Gender in Research

Laboratory animals	
Wild animals	
Reporting on sex	
Field-collected samples	
Ethics oversight	

Note that full information on the approval of the study protocol must also be provided in the manuscript.

Clinical data

Policy information about clinical studies

All manuscripts should comply with the ICMJE guidelines for publication of clinical research and a completed CONSORT checklist must be included with all submissions.

Clinical trial registration	ClinicalTrials.gov NCT04245839
Study protocol	Provided with submission
Data collection	At participating study sites, between July 14, 2020 and January 27, 2023
Outcomes	Provided in Methods section, pages 35-38, Study endpoints: The primary endpoint was ORR per IRC by PET/CT per Lugano 20142. Secondary efficacy endpoints were CR rate, DOR, DOR in patients with a BOR of CR, PFS, and OS. PET/CT assessments were performed at screening, at days 29 and 90, and months 6, 9, 12, 18, 24, 36, 48, and 60. Additional secondary endpoints were safety, cellular kinetics, and PROs. Efficacy subgroup analyses (conducted for subgroups with ≥5 patients) and peripheral B-cell aplasia were assessed as exploratory endpoints. TEAEs were defined as an AE that started from liso-cel administration through and including 90 days after liso-cel infusion. AEs of special interest included infusion-related reaction, CRS, NEs (defined as investigator-identified neurological AEs related to liso-cel), MAS/HLH, tumor lysis syndrome (TLS), grade ≥3 infection, prolonged cytopenias (defined as grade ≥3 laboratory abnormalities of neutropenia, anemia, or thrombocytopenia at day 29), hypogammaglobulinemia, and second primary malignancy. Hypogammaglobulinemia and second primary malignancy could have occurred within or beyond the 90-day TEAE period. Reporting of AEs was based on MedDRA and NCI CTCAE, version 5.0, with the exception of CRS, which was graded according to Lee 2014 criteria3, and TLS, which was graded according to Cairo and Bishop4. NEs were defined as investigator-identified neurological AEs related to liso-cel (captured on the AE domain of the electronic case report form (eCRF) using the preferred term, "neurotoxicity") and graded using NCI CTCAE, version 5.0, on the basis of the highest individual symptom grade. Symptoms of NEs were defined as investigator-identified events entered on the "Clinical Events— Neurotoxicity Details" record in the eCRF from verbatim terms in patients who received liso-cel and for which the question, "Is this event related to liso-cel?" had been answered with "suspected" on the neurotoxicity AE eCRF. Reporting of hypogammaglobulinemia was based on AEs that occurred on or after the liso-cel infusion date and were coded to the following MedDRA preferred terms: blood Ig A decreased, blood IgG decreased, blood IgM decreased, hypogammaglobulinemia, immunoglobulins decreased, selective IgA immunodeficiency, selective IgG subclass deficiency, and selective IgM immunodeficiency. Reporting of second primary malignancy was based on findings from Standardised MedDRA Queries (SMQ) searches for "premalignant disorders" and "malignancies" and subsequent medical review by an internal adjudication panel. The process consisted of a review of preferred terms detected during an SMQ search of all reported AEs and selecting those deemed appropriate for inclusion as malignancies. Cellular kinetic analyses of liso-cel in peripheral blood were performed in the cellular kinetic set, which included patients in the liso-cel–treated set with any available measurements of cellular kinetics by polymerase chain reaction. Concentration values after new anti-FL treatment were excluded from the summaries. B-cell aplasia was defined as CD19+ B cells representing <3% of peripheral blood lymphocytes as measured by flow cytometry.<br>PROs were evaluated in the PRO analysis set (i.e., patients in the liso-cel–treated set who completed a pre-LDC baseline visit and ≥1 postbaseline PRO measurement) using the EORTC QLQ-C30, FACT-LymS, and EQ-5D-5L (health utility index and visual analogue scale). We present results for primary domains of interest preselected for relevance to FL, which included six EORTC QLQ-C30 domains (global health status/quality of life, physical functioning, role functioning, cognitive functioning, fatigue, and pain) and the FACT-LymS subscale assessing lymphoma-specific symptoms. Results for the EORTC QLQ-C30 secondary domains are also presented. The completion rates, defined as the number of patients submitting a valid PRO assessment at a given time point over the number of patients who are still expected to submit a PRO assessment at that time point, were calculated for all PRO questionnaires. At the group level, PROs were analyzed based on the mean changes from baseline at each study visit. Per Supplementary Table 20, minimally clinically important differences were based on published thresholds5,6. Additional PRO assessments conducted as post-hoc analyses included a linear mixed-effects model for repeated measures to estimate least squares mean changes from baseline for primary and secondary domains, time to confirmed improvement for primary domains based on Kaplan-Meier methodology, and proportions of patients with clinically meaningful improvement or deterioration from baseline for primary domains based on individual-level descriptive analyses.

Dual use research of concern

Policy information about dual use research of concern

Hazards

Could the accidental, deliberate or reckless misuse of agents or technologies generated in the work, or the application of information presented in the manuscript, pose a threat to:

No | Yes
- [ ] [ ] Public health
- [ ] [ ] National security
- [ ] [ ] Crops and/or livestock
- [ ] [ ] Ecosystems
- [ ] [ ] Any other significant area

Experiments of concern

Does the work involve any of these experiments of concern:

No | Yes
- [ ] [ ] Demonstrate how to render a vaccine ineffective
- [ ] [ ] Confer resistance to therapeutically useful antibiotics or antiviral agents
- [ ] [ ] Enhance the virulence of a pathogen or render a nonpathogen virulent
- [ ] [ ] Increase transmissibility of a pathogen
- [ ] [ ] Alter the host range of a pathogen
- [ ] [ ] Enable evasion of diagnostic/detection modalities
- [ ] [ ] Enable the weaponization of a biological agent or toxin
- [ ] [ ] Any other potentially harmful combination of experiments and agents

Plants

Seed stocks

Novel plant genotypes

Authentication

ChIP-seq

Data deposition

- [ ] Confirm that both raw and final processed data have been deposited in a public database such as GEO.
- [ ] Confirm that you have deposited or provided access to graph files (e.g. BED files) for the called peaks.

Data access links
May remain private before publication.

Files in database submission

Genome browser session
(e.g. UCSC)

Methodology

Replicates

Sequencing depth

Antibodies

Peak calling parameters

Data quality

Software

Flow Cytometry

Plots

Confirm that:

- [ ] The axis labels state the marker and fluorochrome used (e.g. CD4-FITC).
- [ ] The axis scales are clearly visible. Include numbers along axes only for bottom left plot of group (a 'group' is an analysis of identical markers).
- [ ] All plots are contour plots with outliers or pseudocolor plots.
- [ ] A numerical value for number of cells or percentage (with statistics) is provided.

Methodology

Sample preparation

Instrument

Software

Cell population abundance

Gating strategy

- [ ] Tick this box to confirm that a figure exemplifying the gating strategy is provided in the Supplementary Information.

Magnetic resonance imaging

Experimental design

Design type

Design specifications

Behavioral performance measures

Imaging type(s)

Field strength

Sequence & imaging parameters

Area of acquisition

Diffusion MRI [ ] Used [ ] Not used

Preprocessing

Preprocessing software

Normalization

Normalization template

Noise and artifact removal

Volume censoring

Statistical modeling & inference

Model type and settings

Effect(s) tested

Specify type of analysis: [ ] Whole brain [ ] ROI-based [ ] Both

Statistic type for inference

(See Eklund et al. 2016)

Correction

Models & analysis

n/a	Involved in the study
☐	☐ Functional and/or effective connectivity
☐	☐ Graph analysis
☐	☐ Multivariate modeling or predictive analysis

Functional and/or effective connectivity

Graph analysis

Multivariate modeling and predictive analysis

This checklist template is licensed under a Creative Commons Attribution 4.0 International License, which permits use, sharing, adaptation, distribution and reproduction in any medium or format, as long as you give appropriate credit to the original author(s) and the source, provide a link to the Creative Commons license, and indicate if changes were made. The images or other third party material in this article are included in the article's Creative Commons license, unless indicated otherwise in a credit line to the material. If material is not included in the article's Creative Commons license and your intended use is not permitted by statutory regulation or exceeds the permitted use, you will need to obtain permission directly from the copyright holder. To view a copy of this license, visit http://creativecommons.org/licenses/by/4.0/
